# Using outbreak data to estimate the dynamic COVID-19 landscape in Eastern Africa

**DOI:** 10.1186/s12879-022-07510-3

**Published:** 2022-06-09

**Authors:** Mark Wamalwa, Henri E. Z. Tonnang

**Affiliations:** International Centre of Insect Physiology and Ecology (Icipe), P.O. Box 30772-00100, Nairobi, Kenya

**Keywords:** COVID-19, eSIR model, Runge–Kutta approximation, Basic reproduction number, Epidemic trend

## Abstract

**Background:**

The emergence of COVID-19 as a global pandemic presents a serious health threat to African countries and the livelihoods of its people. To mitigate the impact of this disease, intervention measures including self-isolation, schools and border closures were implemented to varying degrees of success. Moreover, there are a limited number of empirical studies on the effectiveness of non-pharmaceutical interventions (NPIs) to control COVID-19. In this study, we considered two models to inform policy decisions about pandemic planning and the implementation of NPIs based on case-death-recovery counts.

**Methods:**

We applied an extended susceptible-infected-removed (eSIR) model, incorporating quarantine, antibody and vaccination compartments, to time series data in order to assess the transmission dynamics of COVID-19. Additionally, we adopted the susceptible-exposed-infectious-recovered (SEIR) model to investigate the robustness of the eSIR model based on case-death-recovery counts and the reproductive number (R_0_). The prediction accuracy was assessed using the root mean square error and mean absolute error. Moreover, parameter sensitivity analysis was performed by fixing initial parameters in the SEIR model and then estimating R_0_, β and γ.

**Results:**

We observed an exponential trend of the number of active cases of COVID-19 since March 02 2020, with the pandemic peak occurring around August 2021. The estimated mean R_0_ values ranged from 1.32 (95% CI, 1.17–1.49) in Rwanda to 8.52 (95% CI: 3.73–14.10) in Kenya. The predicted case counts by January 16/2022 in Burundi, Ethiopia, Kenya, Rwanda, South Sudan, Tanzania and Uganda were 115,505; 7,072,584; 18,248,566; 410,599; 386,020; 107,265, and 3,145,602 respectively. We show that the low apparent morbidity and mortality observed in EACs, is likely biased by underestimation of the infected and mortality cases.

**Conclusion:**

The current NPIs can delay the pandemic pea and effectively reduce further spread of COVID-19 and should therefore be strengthened. The observed reduction in R_0_ is consistent with the interventions implemented in EACs, in particular, lockdowns and roll-out of vaccination programmes. Future work should account for the negative impact of the interventions on the economy and food systems.

**Supplementary Information:**

The online version contains supplementary material available at 10.1186/s12879-022-07510-3.

## Background

Coronavirus Disease 2019 (COVID-19) is a zoonotic disease caused by the Severe Acute Respiratory Syndrome Corona Virus 2 (SARS-CoV-2), a pathogen that was first discovered in Wuhan, China in 2019 [1–3]. Consequently, the disease has spread all over the world, leading to high morbidity and mortality in addition to negatively impacting the healthcare systems and the economy [4, 5]. Following the first case report in Egypt on the 14th February, 2020, a total of 6,543,882 cases and 166,234 deaths had been recorded in 54 African countries by 28th July 2021 [6]. Regionally, Eastern Africa countries (EACs) have not been spared the impact of the pandemic with the following reported cases and mortalities by the 28th July 2021: (Burundi 6573 cases, 8 deaths; Ethiopia 278,920 cases, 4374 deaths; Kenya 198,935 cases, 3882 deaths; Rwanda 66,967 cases, 771 deaths; South Sudan, 11,014 cases, 118 deaths; United Republic of Tanzania, 858 cases, deaths 29 deaths; Uganda, 92,795 cases, 2590 deaths) [6]. Initial reports indicate that majority of the early cases were likely imported from Asia and Europe through trade and tourism [7].

In this regard, policymakers made decisions to mitigate the pandemic future scenarios by implementing NPIs to varying degrees of success [8–10]. Similar intervention measures were successfully applied to mitigate the influenza virus transmission [11]. It is nevertheless noteworthy that the time point of implementation of these interventions is key to their success in reducing the peak of the pandemic [12]. Additionally, these measures should be appropriately justified to the population in terms of the optimal time when they could be eased [13].

As the pandemic evolved, mathematical models were developed to estimate the transmission dynamics over time, with the expectation that the pandemic will have a devastating impact across Africa [14–16]. For example, the University of Washington, Seattle, developed the Institute of Health Metrics (IHME) for fitting parametrized curves to COVID-19 data using extendable nonlinear mixed effects model [17]. The Imperial College London (ICL), developed a semi-mechanistic Bayesian model to estimate the rate of transmission, total number of cases and deaths at a given time point, and the impact of NPIs on the basic reproduction number (R_0_) as well as the time-varying reproduction number, R(t) [14]. R_0_ is a measure of contagiousness of infectious agents, and it refers to the number of new infections generated by each infected person [18]. If R_0_ < 1, the disease will decline spreading in the population, and if R_0_ > 1, the disease will spread faster [19]. Moreover, compartmental models have long been used to model the dynamics of infectious diseases including influenza [20–22]. These models use ordinary differential equations that mimic infectious disease trajectory, and a three- or four-state Markov chain to solve the equations [23].

**Fig. 1:**
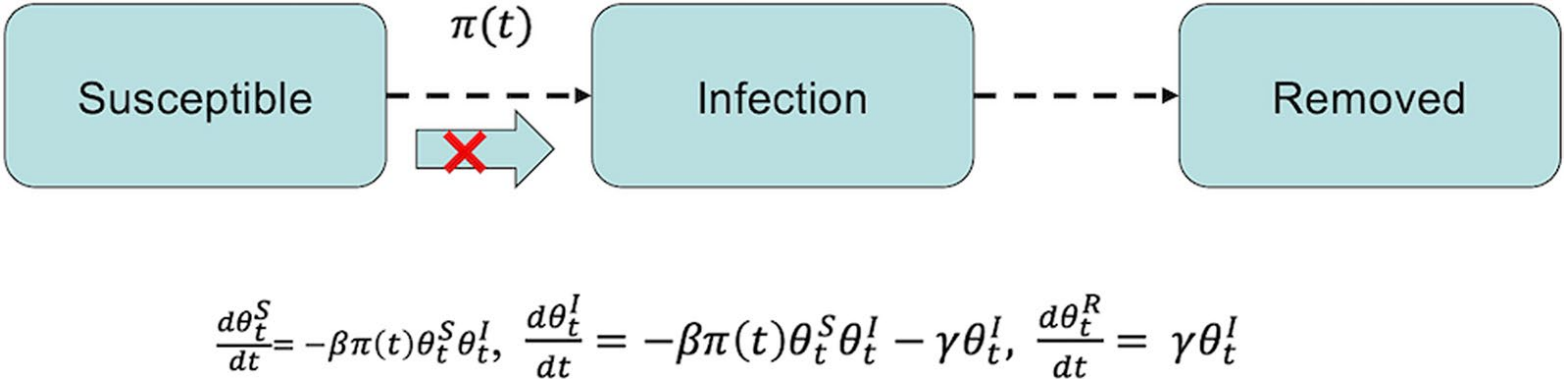
The extended Susceptible-Exposed-Removed (eSIR) basic model diagram. The transmission rate modifier, π(t), takes on values according to actual interventions in different countries [25]

Recently, the classical susceptible-infectious-recovered (SIR) model was extended to simulate NPIs such as quarantine, and national lockdowns using time-varying functions that modify the transmission rate of the disease [24–26]. The eSIR model uses three compartments—susceptible, infected, and removed (sum of recovered and dead) and a Bayesian hierarchical model to simulate future projections of the number of infected and removed population [25, 26]. The standard eSIR model assumes a constant transmission rate through the compartments. This rate can be altered to mimic NPIs by introducing a transmission modifier (π_(t)_) to allow a time-varying probability of the transmission rate (Fig. 1).

Additionally, the eSIR model also assumes that probabilities of the three compartments follow a Markov transition process with input as the proportions of infected and removed (sum of recovered and dead) cases. The observed proportions of infected and removed cases on day t are denoted by *Y*_t_^*I*^ and *Y*_t_^*R*^, respectively. The true underlying probabilities of the S, I, and R compartments on day t are denoted by θ_t_^*S*^, θ_t_^*I*^, and θ_t_^*R*^, respectively, and assume that for any t, θ_t_^*S*^ + θ_t_^*I*^ + θ_t_^*R*^ = 1, which can be solved through ordinary differential equations (Eqs. 1–3).

1 $$\frac{{d\theta }_{t}^{S}}{dt}= -\beta {\theta }_{t}^{S}{\theta }_{t}^{I}$$

2 $$\frac{{d\theta }_{t}^{I}}{dt}= -\beta {\theta }_{t}^{S}{\theta }_{t}^{I}-\gamma {\theta }_{t}^{I},$$

3 $$\frac{{d\theta }_{t}^{R}}{dt}= \gamma {\theta }_{t}^{I}$$

whereby, *β* > 0 is the disease transmission rate, and γ > 0 is the removal rate. R_0_ = β/γ is the basic reproduction number assuming the whole population is susceptible. The basic eSIR model applies a Beta-Dirichlet state-space consisting of three observations of infected (*Y*_t_^*I*^), removed (*Y*_t_^*R*^) and the latent process at time *t* [25–27].

4 $${Y}_{t}^{I}|{\theta }_{t},\tau \sim Beta\left({\lambda }^{I}{\theta }_{t}^{I}, {\lambda }^{I}\left(1-{\theta }_{t}^{I}\right)\right),$$

5 $${Y}_{t}^{R}|{\theta }_{t},\tau \sim Beta\left({\lambda }^{R}{\theta }_{t}^{R}, {\lambda }^{I}\left(1-{\theta }_{t}^{R}\right)\right),$$

The latent population prevalence is represented below as a Markov process (Eq. 6).

6 $${\theta }_{t}|{\theta }_{t-1,}\tau \sim Dirichlet(kf ({\theta }_{t-1,}\beta ,\gamma ))$$

where ${{\theta }_{t}=({\theta }_{t}^{S}, {\theta }_{t}^{I},{\theta }_{t}^{R})}^{\rm T}$ is the prevalence of susceptible population (${\theta }_{t}^{S}$), infectious (${\theta }_{t}^{I}$) and removed (${\theta }_{t}^{R}$) populations at time *t*, while $\tau =(\beta ,\gamma ,{\theta }_{0}^{T},\lambda ,k)$ denotes parameters ${\lambda }^{I}$, ${\lambda }^{R}$ and $k$ that control variances of the infected, removed and latent processes respectively [25, 26]. The function *f* is the solution to the standard SIR model using ordinary differential equations (Eqs. 1–3) and a fourth order Runge–Kutta (RK4) approximation [28, 29].

**Fig. 2:**
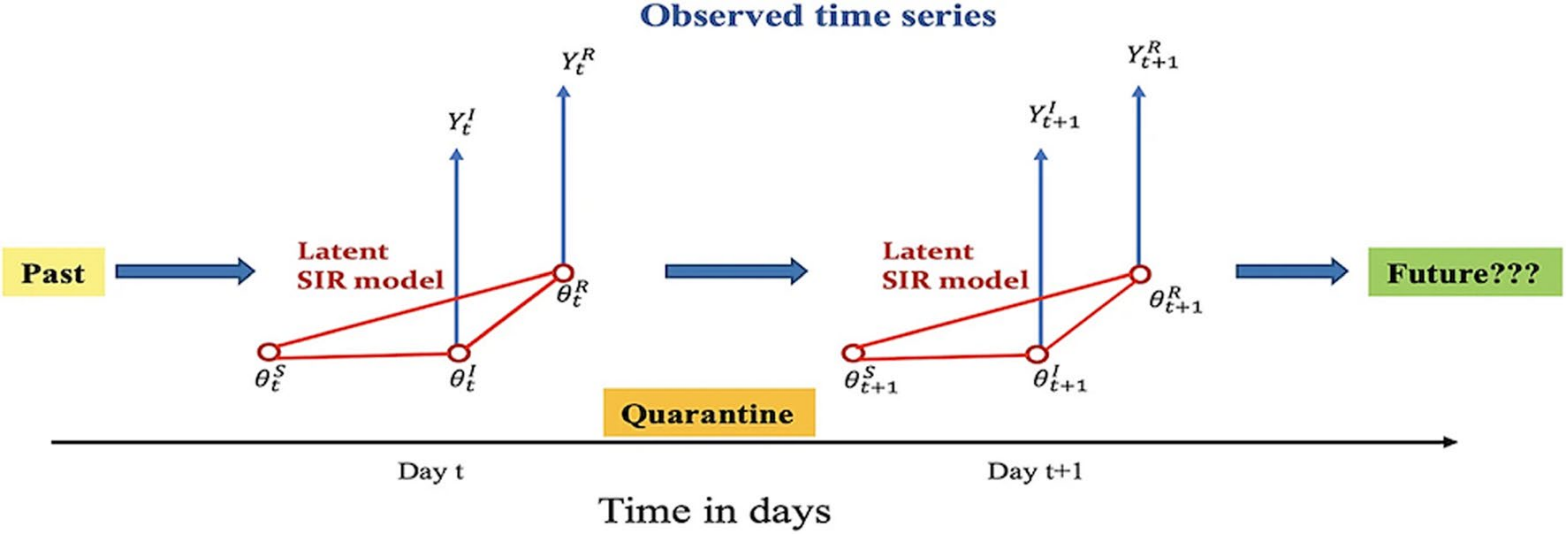
The eSIR model with a state-space latent SIR model. The latent Markov (${\theta }_{t}$) processes are sampled and forecasted by the MCMC sampler Reproduced from Wang et al. 2020 [25]

The Markov chain Monte Carlo (MCMC) algorithm was used to implement this model in order to provide the posterior estimates and credible intervals of the unknown parameters, R_0_, β, and γ [19, 25]. ﻿ Previously, Mkhatshwa et al. and Wangping et al. reported that MCMC prior distributions can be initialized according to the SARS data from Hong Kong [26, 30]. The MCMC algorithm samples the latent Markov processes and estimates the infection prevalence (${\theta }_{t}^{I}$) and the probability of removal (${\theta }_{t}^{R}$) from the underlying latent dynamics of COVID-19 infection (Fig. 2) [25, 26]. These estimates determine the epidemic turning points and R_0_ of the target population. Nevertheless, it is noteworthy that the details of the eSIR model formulation are described in detail in [25, 26].

Mathematical models leverage available data to predict transmission dynamics of the epidemic and the impact of different policy interventions. These models have been a critical tool for COVID-19 policy decisions [31]. However, foreseen risks include under-estimation of the disease extend due to asymptomatic cases that account for the majority of the transmission [32]. Models such as the classical SIR and eSIR do not account for the pre-symptomatic and asymptomatic cases. Indeed, similar studies have used SEIR extensions to account for the pre-symptomatic and asymptomatic infection [32–38].

Contemporary models have consistently predicted that the ongoing COVID-19 pandemic will have a devastating impact across Africa [14–16]. Beyond health risks, the socio-economic implications of the pandemic motivated the current research to exploit a data-driven approach for deducing the transmission dynamics of the pandemic, infection prevention and evaluating policy implementation. This study sought to predict COVID-19 epidemiological trends under current and future scenarios in seven EACs and to quantify the impact of the interventions in flattening the pandemic curve. However, we did not consider the impact of the interventions on the economy and food systems.

## Methods

In this work, we applied the extended Susceptible-Exposed-Removed (eSIR) compartmental model to project epidemiological trends of COVID-19 infections and the impact of government interventions in Burundi, Ethiopia, Kenya, Rwanda, South Sudan, Tanzania and Uganda [25].

### Data sources

We used publicly available COVID-19 daily recorded time-series data of the seven EACs collated from the WHO and the Johns Hopkins University Center for Systems Science and Engineering (JHU CCSE) to estimate the transmission of the epidemic and to present the trend of infections and fatalities [39, 40]. These datasets include daily counts of confirmed cases, recovered cases, and deaths from 22nd January 2020 to 30th July 2021.

### Epidemiological modelling

Modelling the impact of NPIs was implemented in R (version 4.0.4) using the eSIR model to simulate future projections of cases-deaths-recovery counts [26]. The resulting differential equations were solved by the fourth-order Runge–Kutta approximation [28, 29]. The input data was segmented into two starting from March 02/2020 to May 01/2020 and the same time period for the year 2021. The R_0_ and R(t) were estimated using MCMC algorithm implemented in RJAGS and presented using the resulting posterior mean and 95% credible interval (CI) [41].

**Fig. 3:**
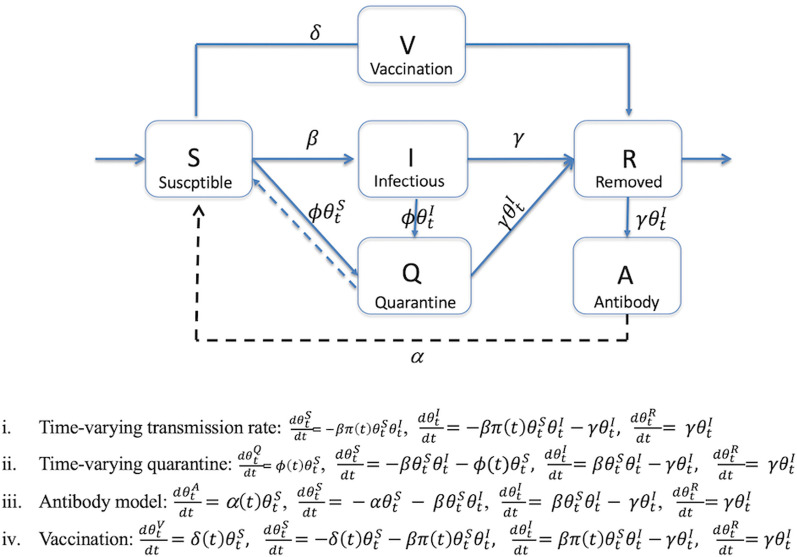
Flow diagram of the underlying states of COVID-19 eSIR model. The flow diagram was used to obtain transmission probabilities according to actual interventions in different countries

Model predictions were interpreted based on the turning points of the projected epidemiological trend of COVID-19. The first turning point refers to the mean predicted time when the daily proportion of infected cases becomes lower than the previous infected cases, while the second turning point refers to the mean predicted time when the daily proportion of removed cases (sum of recovered and dead) becomes higher than the infected cases. Similarly, an end point refers to the time point when the median proportion of current infected cases turns to zero [25, 26]. The basic SIR model does not consider NPIs in the estimation of the epidemic trajectory, hence we used the time-varying transmission (*tvt*) rate SIR model, SIR with time-varying quarantine, antibody (herd immunity) and vaccination compartments to project future scenarios (Fig. 3).

### SIR model with a time-varying transmission rate

A time-dependent rate parameter [π(t)] was introduced to vary the transmission rate (β) and the average removal rate (γ) according to the NPIs that were introduced in each country. The prior distribution of beta (β) was set to 0.2586, and gamma (γ) was set to 0.0821 [25, 26]. The likelihood of disease transmission under the t*vt* rate model when a susceptible individual comes into contact with an infected individual is presented below (Eq. 7).

7 $$\beta \left\{1-{q}^{S}(t)\right\}{\theta }_{t}^{S}\left\{1-{q}^{I}(t)\right\}{\theta }_{t}^{I}:=\beta \pi (t){\theta }_{t}^{S}{\theta }_{t}^{I}$$

where, $\pi \left(t\right) :=\left\{1-{q}^{S}\left(t\right)\right\}\left\{1-{q}^{I}\left(t\right)\right\}\in \left[\mathrm{0,1}\right]$. The parameter ${q}^{S}\left(t\right)\in \left[\mathrm{0,1}\right]$ is the likelihood of a susceptible individual being put under isolation. Similarly, ${q}^{I}\left(t\right)\in \left[\mathrm{0,1}\right]$ is the likelihood of an infected individual being put under isolation.

When applied as a step function, $\pi \left(t\right)$ assumes four values that correspond to the dates when NPIs were introduced in each country. For example, the first confirmed cases of COVID-19 in the seven EACs were reported in early March 2020 and thereafter, several intervention measures were introduced. For example, ${\pi }_{01}$ corresponds to the time period when no interventions had been initiated, ${\pi }_{02}$ to the time period when national/city lockdowns were initiated, ${\pi }_{03}$ corresponds to enhanced quarantine measures while ${\pi }_{04}$ corresponds to opening of new medical facilities for covid patients.

$$ \pi \left(t\right)=\left\{\begin{array}{l}{\pi }_{01}, if\ t \le Feb\ 23, no\ defined\ interevntion\ measures; \\ {\pi }_{02}, if\ t \le Mar\ 15, partial\ lockdowns,\ school\ closure; \hfill \\ {\pi }_{03}, if\ t \le Apr\ 06, enhanced\ quarantine, curfews; \\ {\pi }_{04}, if\ t \le Apr\ 27, new\ quarantine\ facilities, hospitals\ opened.\end{array}\right.$$

Alternatively, $\pi \left(t\right)$ can be applied as an exponential function to reflect gradual increase in awareness to the pandemic and response to government interventions, where $\pi \left(t\right)=\mathrm{exp}(-{\lambda }_{0}t)$. However, at any given time, $\pi \left(t\right)=1$ if no intervention measure has been imposed. The function *f* is the solution the eSIR model using Eq. 8 and RK4 approximation.

8 $$\frac{{d\theta_{t}^{S} }}{dt} = - \beta \pi \left( t \right)\theta_{t}^{S} \theta_{t}^{I} ,\frac{{d\theta_{t}^{I} }}{dt} = - \beta \pi \left( t \right)\theta_{t}^{S} \theta_{t}^{I} - \gamma \theta_{t}^{I} ,\frac{{d\theta_{t}^{R} }}{dt} = \gamma \theta_{t}^{I}$$

The following parameters were used to run the *tvt* rate model: the transition rate modifier, π(t), was allowed to vary between (1.0, 0.9, 0.5, and 0.1) according to actual governmental interventions. This was set at π(t) = 0.95 if t < Mar 10, for city lockdown; π(t) = 0.9 if t ∈ (Mar 10, Mar 22), country lockdown; π(t) = 0.5 if t ∈ (Mar 15, April 01), shutdown of schools and non-essential businesses; π(t) = 0.1 if t > Mar 31, which corresponds to more enhanced quarantine protocols [26]. The value of $\pi \left(t\right)$ was estimated from Eq. 7, where$\pi \left(t\right) :=\left\{1-{q}^{S}\left(t\right)\right\}\left\{1-{q}^{I}\left(t\right)\right\}\in \left[\mathrm{0,1}\right]$. The proportion of deaths within the removed compartment was estimated from a pre-selected ratio of 0.0184 and the initial infection fatality ratio was set to 0.01 [42]. MCMC simulation was performed using four parallel chains, with the number of draws in each chain, M = 5e5 and a burn-in period of *nburnin* = 2e3 under 2 × 10^5^ iteration number of adaptation in the MCMC (*nadapt* = 2 × 10^5^) [25]. The output of these runs provided estimates of posterior parameters and prevalence of the disease in the six compartments of the modified eSIR model and proportions of the infected and the removed individuals (Fig. 3).

### SIR with time-varying quarantine

We simulated the impact of quarantine measures by including a fourth compartment of the population under quarantine [25, 26]. A vector *phi* (ϕ) that assumes a Dirac delta function (a point mass prior at 0.1–0.4) was used to alter transition from susceptible to the quarantine compartment at time points corresponding to the days when quarantine measures were imposed in each country [20]. The time-varying quarantine model was simulated using parameters described in the tvt rate model (Eq. 9).

9 $$\frac{{d\theta_{t}^{Q} }}{dt} = \phi \left( t \right)\theta_{t}^{S} ,\frac{{d\theta_{t}^{S} }}{dt} = - \beta \theta_{t}^{S} \theta_{t}^{I} - \phi \left( t \right)\theta_{t}^{S} ,\frac{{d\theta_{t}^{I} }}{dt} = \beta \theta_{t}^{S} \theta_{t}^{I} - \gamma \theta_{t}^{I} ,\frac{{d\theta_{t}^{R} }}{dt} = \gamma \theta_{t}^{I}$$

The quarantine rate, $\phi \left(t\right)$, was specified according to the time points when NPIs were enforced in each country.

$$\phi \left(t\right)=\left\{\begin{array}{l}{\phi }_{01}, if \, t = Feb \, 23, no defined \, interevntion \, measures; \\ {\phi }_{02}, if \, t = Mar \, 15, partial \, lockdowns, \, school \, closure; \\ {\phi }_{03}, if \, t = Apr \, 6, enhanced \, quarantine, \, curfews; \\ 0, \, otherwise.\end{array}\right.$$

### Herd immunity

We introduced an antibody (A) compartment to simulate the presence of natural acquired immunity against COVID-19 within the population and thereby altering the eSIR to eSAIR model [26, 27]. The A compartment consists of infected (I) but recovered/self-immunized individuals, with rate constants determining transition between the four compartments of Susceptible, Antibody, Infected and Removed (SAIR). The model was run using time-varying transmission rate parameters described above with the assumption that about 20% (α = 0.2) of the susceptible population had acquired neutralizing antibodies against SARS-CoV-2 (Eq. 10).

10 $$\frac{{d\theta_{t}^{A} }}{dt} = \left( t \right)\theta_{t}^{S} ,\frac{{d\theta_{t}^{S} }}{dt} = - \alpha \theta_{t}^{S} - \beta \theta_{t}^{S} \theta_{t}^{I} ,\frac{{d\theta_{t}^{I} }}{dt} = \beta \theta_{t}^{S} \theta_{t}^{I} - \gamma \theta_{t}^{I} ,\frac{{d\theta_{t}^{R} }}{dt} = \gamma \theta_{t}^{I}$$

The probability of having neutralizing antibodies against COVID-19 was denoted by theta ($d{\theta }_{t}^{A}$) at time point *t*, where α$\left(t\right)$ is a function that determines the proportion of people moved into the antibody (A) compartment from the susceptible compartment.

### Vaccination

The vaccination (V) compartment was integrated into the basic SIR model and thereby transforming the eSIR into eSVIR model. The model was run using time-varying transmission rate parameters under the assumption that about 2% (α = 0.02) of the susceptible population was vaccinated (Eq. 11). However, Tanzania only began their vaccination campaign in July 2021, while Burundi was yet to receive vaccine doses.

11 $$\frac{{d\theta_{t}^{V} }}{dt} = \delta \left( t \right)\theta_{t}^{S} , \frac{{d\theta_{t}^{S} }}{dt} = - \delta \left( t \right)\theta_{t}^{S} - \beta \pi \left( t \right)\theta_{t}^{S} \theta_{t}^{I} , \frac{{d\theta_{t}^{I} }}{dt} = \beta \pi \left( t \right)\theta_{t}^{S} \theta_{t}^{I} - \gamma \theta_{t}^{I} , {\text{and}} \frac{{d\theta_{t}^{R} }}{dt} = \gamma \theta_{t}^{I}$$

### Validation of the model prediction accuracy

The reliability and usefulness of our approach, was evaluated by comparing model predictions of case-death-recovery counts against the observed data between 06/16/20 and 04/11/2021 in Ethiopia, Kenya, Rwanda and Uganda using two metrics, the Root Mean square error (RMSE), and Mean Absolute Error (MAE) [27]. RMSE is a measure of the differences between predicted and the observed values for a given variable in a regression analysis (Eq. 12a) while MAE measures the accuracy of the model fit in terms of performance in its predictions (Eq. 12b) [43]. The input data was split into two sets for training and validation of the model [44]. The training dataset ranged from 16th June 2020 to 12th January 2021 while the validation dataset was from 13th January 2021 to 2nd April 2021. Specifically, the model was calibrated using observed data of confirmed case-death-recovery counts (“training set”) starting from the date of implementation of the intervention up to 7–14 days prior to the peaks. Thereafter, model predictions (“testing set”) of case-counts after the training period were then compared with the observed trends to evaluate the prediction accuracy. A total of 291 datapoints were used to compute the RMSE and MAE values [43, 45].

12a $$RMSE= \sqrt{\frac{1}{n}\sum_{i=1}^{n}{{(Y}_{i}-{\widehat{Y}}_{i})}^{2}}$$

12b $$MAE= \frac{{\sum }_{i=1}^{n}|{\widehat{Y}}_{i}-{Y}_{i}|}{n}$$

where, n is the total number of observations, ${Y}_{i}$ the predicted value and ${\widehat{Y}}_{i}$ the observed value for the i^th^ observation.

### Comparison of the eSIR and SEIR models

The eSIR model used in this study does not account for the pre-symptomatic and asymptomatic cases. We applied a modified SEIR extension implemented in the SEIR-fansy (faLSE nEGATIVE rate and syMPTOM) package to account for the pre-symptomatic and asymptomatic infections [46]. Additionally, the modified SEIR model also takes into account the false negative rates of COVID-19 RT-PCR tests and the unreported/untested cases [46]. COVID-19 cases-deaths-recovery count data reported in seven EACs from April 01/2020 to June 31/2020 was used to train the model and generate predictions from August 01 to December 01/2020. Similarly, training data from April 01/2021 to June 31/2021 was used to train the model and generate predictions from August 01 to July 13/2022. The two models were compared with respect to the predicted R(t) values, active cases and deaths.

## Results

### Scenario analysis of COVID-19 epidemic development

Simulation of NPIs estimated the effectiveness of government intervention in curbing the spread of the disease. The predicted R_0_, time varying reproduction number, R(t) (also known as the effective reproduction number, R_e_) and cases-deaths-recovery counts provide an insight into the epidemiological trend of the disease for the year ending 2020/2021 (Table 1). Our results show an exponential increase of cases-deaths-recovery counts since March 02 2020/2021 and a steady decline during the 2021/2022 window. We found that the epidemic peak occurred between March–April and July–August 2021. The estimated posterior values of the time-varying reproduction number R(t) ranged between 2.70–3.10 and 1.32–8.52 under the exponential growth model for the time period of 2020/2021 and 2021/2022 respectively. The mean R(t) was lowest in Kenya in 2020/2021 (R_0_ = 2.70, 95% CI: 1.54—4.67) (Table 1). However, moving into 2021/2022 window, Rwanda had the lowest R(t) (R_0_ = 1.32, 95% CI: 1.17–1.49), (Table 2). The estimated count of infected and removed compartments by January 2022 is alarming, however it includes missed cases, pre-symptomatic and asymptomatic cases (Table 2).

**Table 1:** Estimated R_0_ and endpoint in EACs using the eSIR model for the year 2020/2021

Country	Median	R0		Endpoint		95%CI	
		Mean	95%CI	Mean	Date (range)	Infected^1^	Removed^2^
Burundi	2.58	2.71	1.48–4.58	05/02/20	04/05/20–07/31/20	998 (116–2884)	351 (41–1100)
Ethiopia	2.62	2.75	1.57–4.65	04/27/20	04/04/20–07/01/20	6566 (474–24,130)	5754 (665–20,408)
Kenya	2.57	2.70	1.54–4.67	04/26/20	04/05/20–06/23/20	2572 (455–6876)	2317 (263–7475)
Rwanda	2.96	3.10	3.10–5.22	05/07/20	04/08/20–07/27/20	964 (259–2121)	397 (41–1370)
South Sudan	2.60	2.71	2.71–4.59	05/21/20	04/16/20–09/17/20	2171 (130–10,107)	631 (89–1920)
Tanzania	2.69	2.82	2.82–4.90	05/01/20	04/05/20–07/16/20	4369 (614–14,483)	3353 (428–11,942)
Uganda	2.75	2.87	2.87–4.79	05/06/20	04/06/20–08/03/20	4219 (648–12,354)	2180 (211–8107)

^1^Means of predicted infected population at the endpoint followed by the confidence interval in brackets (α = 0.05)

^2^Means of predicted removed (recovered + deaths) population at the endpoint followed by the confidence interval in brackets (α = 0.05)

**Table 2:** Estimated R_0_ and endpoint in EACs using the eSIR model for the year 2021/2022

Country	R0			Endpoint	95%CI		
	Median	Mean	95%CI	Mean	Date	Infected^1^	Removed^2^
Burundi	2.74	2.84	1.83–4.45	01/16/22	01/16/22	115,505 (109,999–121,264)	153,638 (147,508–159,954)
Ethiopia	1.63	1.64	1.39–1.99	01/16/22	01/16/22	7,072,584 (6,945,505–7,203,084)	19,736,568 (19,521,417–19,952,888)
Kenya	8.39	8.52	3.73–14.10	01/16/22	01/16/22	330,562 (307,493–353,404)	18,248,566 (18,100,299–18,391,438)
Rwanda	1.31	1.32	1.17–1.49	01/16/22	01/16/22	410,599 (399,776–421,528)	1,913,262 (1,891,033–1,934,980)
South Sudan	1.51	1.54	1.19–2.03	01/16/22	01/16/22	386,020 (376,478–396,244)	751,872 (738,686–765,302)
Tanzania	2.46	2.57	1.45–4.31	01/15/22	01/16/22	107,265 (95,757–119,982)	70,197 (60,262–80,013)
Uganda	2.30	2.34	1.67–3.33	01/16/22	01/16/22	3,145,602 (3,089,070–3,205,017)	2,425,643 (2,375,840–2,477,153)

^1^Means of predicted infected population at the endpoint followed by the confidence interval in brackets (α = 0.05)

^2^Means of predicted removed (recovered + deaths) population at the endpoint followed by the confidence interval in brackets (α = 0.05)

### Time-varying changes caused by government interventions

**Fig. 4:**
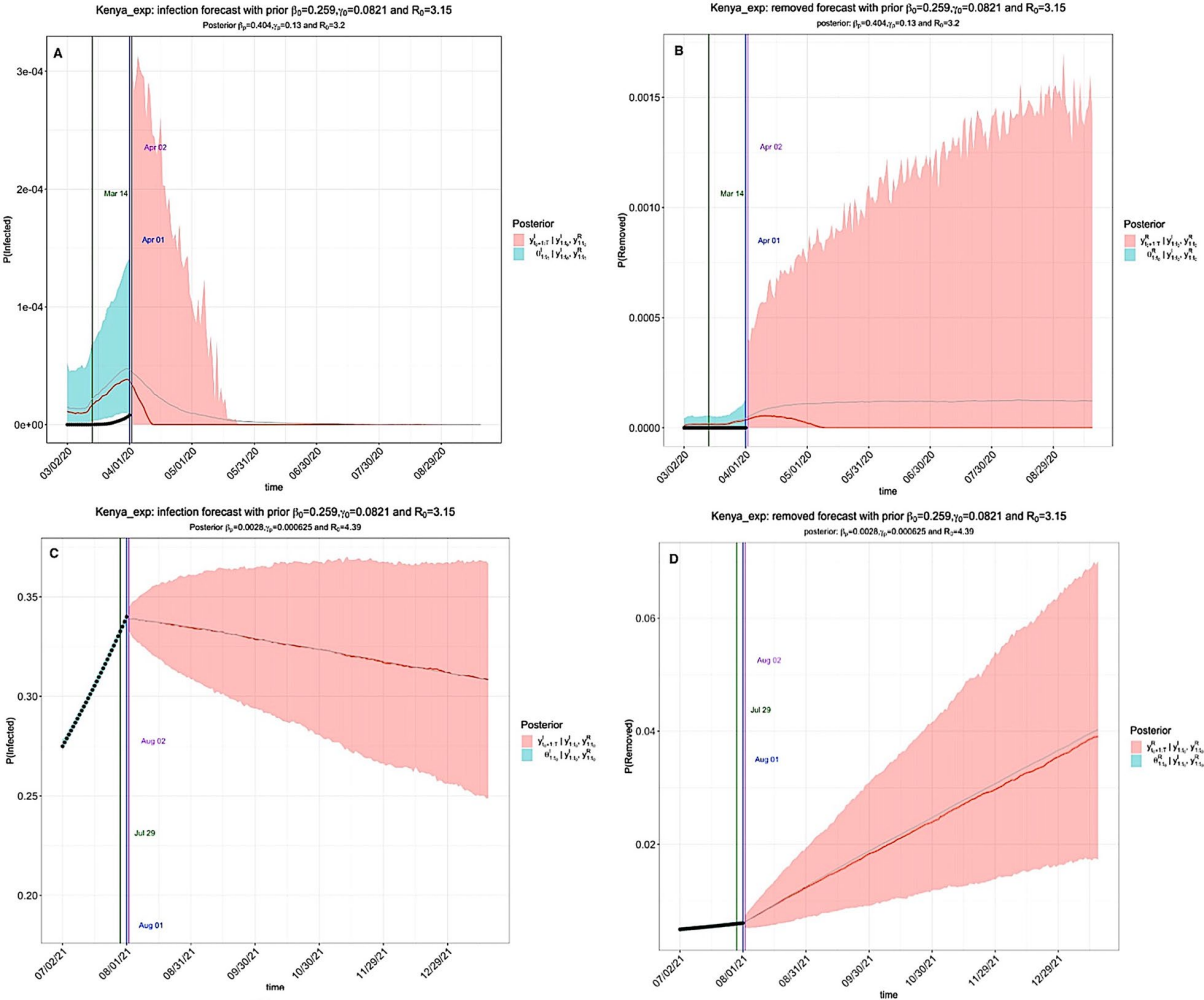
The exponential model of COVID-19 trends under existing interventions in Kenya. The pandemic peaked between March–April 2020 (**A**) and August 2021 (**C**). **A**, **B** Prediction of the infection and removed compartments during the 2020/2021 window. The first and second turning points occurred on Mar 14 and Apr 01 2020; **C**, **D** Prediction of the infection and removed proportions during 2021/2022 window. The first and second turning points occurred on Jul 29 and Aug 01 2021. In Figs. 4, 5, 6, 7 and 8: The black dots left of the blue vertical line denote the observed proportions of the infected and removed compartments. The blue vertical line denotes time *t(0)*. The green and purple vertical lines denote the first and second turning points, respectively. The cyan and salmon colour area denotes the 95% CI of the predicted proportions of the infected and removed cases before and after *t(0)*, respectively. The gray and red curves are the posterior mean and median curves [25, 26]

**Fig. 5:**
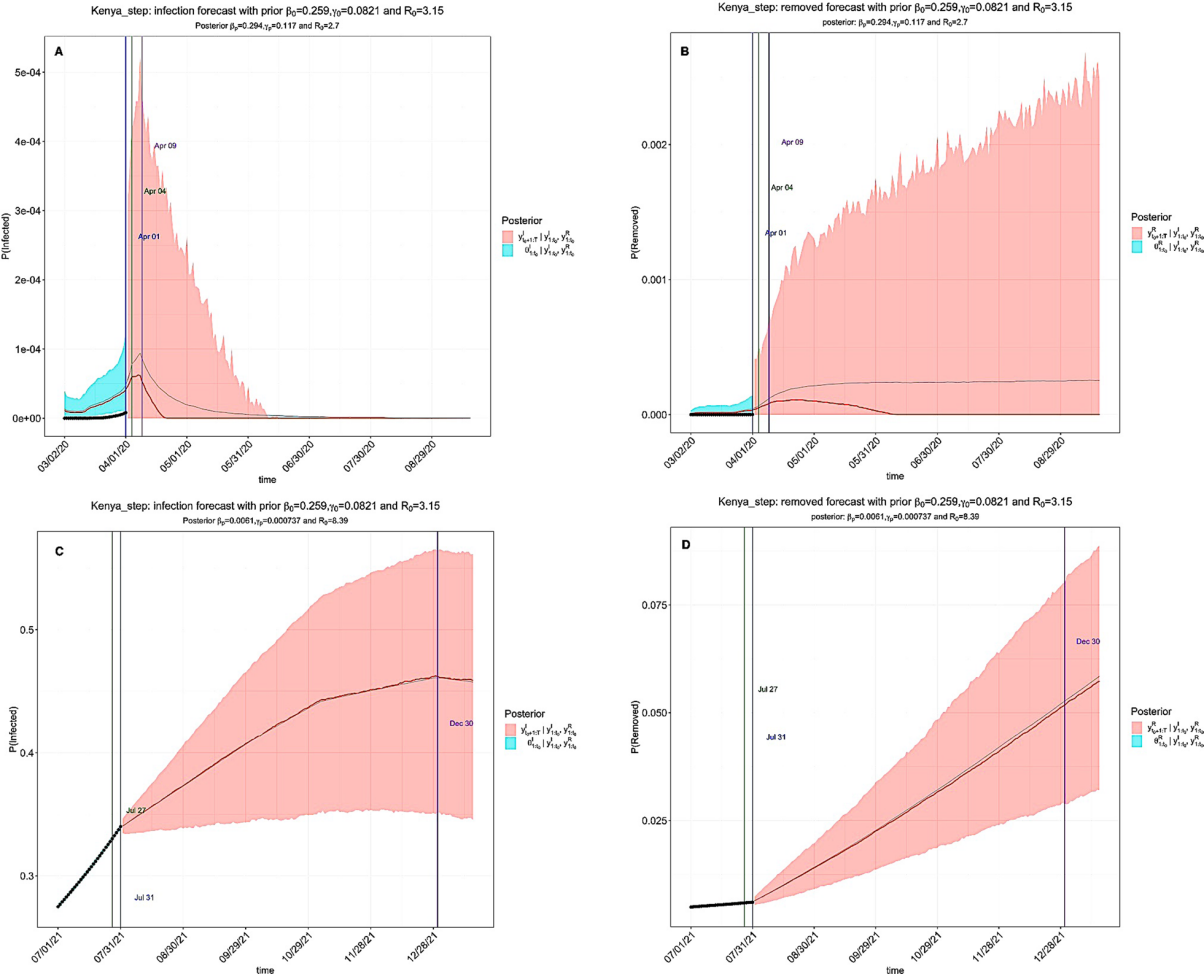
The stepwise model of COVID-19 trends under existing interventions in Kenya. The peak of the pandemic occurred in April 2020 and July 2021. **A**, **B** Prediction of the infection and removed (recovered and dead) proportions *of* COVID-19 during 2020/2021 time period. The first and second turning points occurred on April 01 and April 04. **C**, **D** Prediction of the infection and removed compartments of COVID-19 during the 2021/2022 window. The first and second turning points occurred on July 27 and July 31

COVID-19 pandemic has progressed across EACs with varying impacts. Hyperparameters introduced into the model allowed for inference of the impact of government interventions at specific time points to control the pandemic. For example, the exponential model simulated gradual community awareness of interventions by regional governments (Fig. 4) while the stepwise model simulated NPIs such as school closure, lockdowns and suspension of social gatherings for the year ending 2020/2021 and 2021/2022 (Fig. 5).

We observed an overlap of the epidemiological trend between the exponential and stepwise models. Under the existing preventions in Kenya, the exponential model indicates that the first and second turning points occurred on March 14 and April 01 during the 2020/2021 window (Fig. 4A, B) and Jul 29 and Aug 01 in the 2021/2022 window (Fig. 4C, D). Similarly, the stepwise model indicates that the first and second turning points appeared on April 01 and April 04 (Fig. 5A, B) during the 2020/2021 window and July 27 and July 31 in the 2021/2022 window (Fig. 5C, D). It is noteworthy that the first turning point refers to the mean predicted time when the daily proportion of infected cases becomes lower than the previous infected cases, while the second turning point refers to the mean predicted time when the daily proportion of removed cases (sum of recovered and dead) becomes higher than the infected cases [25, 26]. Additional exponential model projection results are available as additional information (Additional file 1: Figs. S1–S6), while the stepwise model outputs are shown for Burundi, Ethiopia, Rwanda, South Sudan, Tanzania, and Uganda respectively (Additional file 1: Figs. S7–S12).

**Fig. 6:**
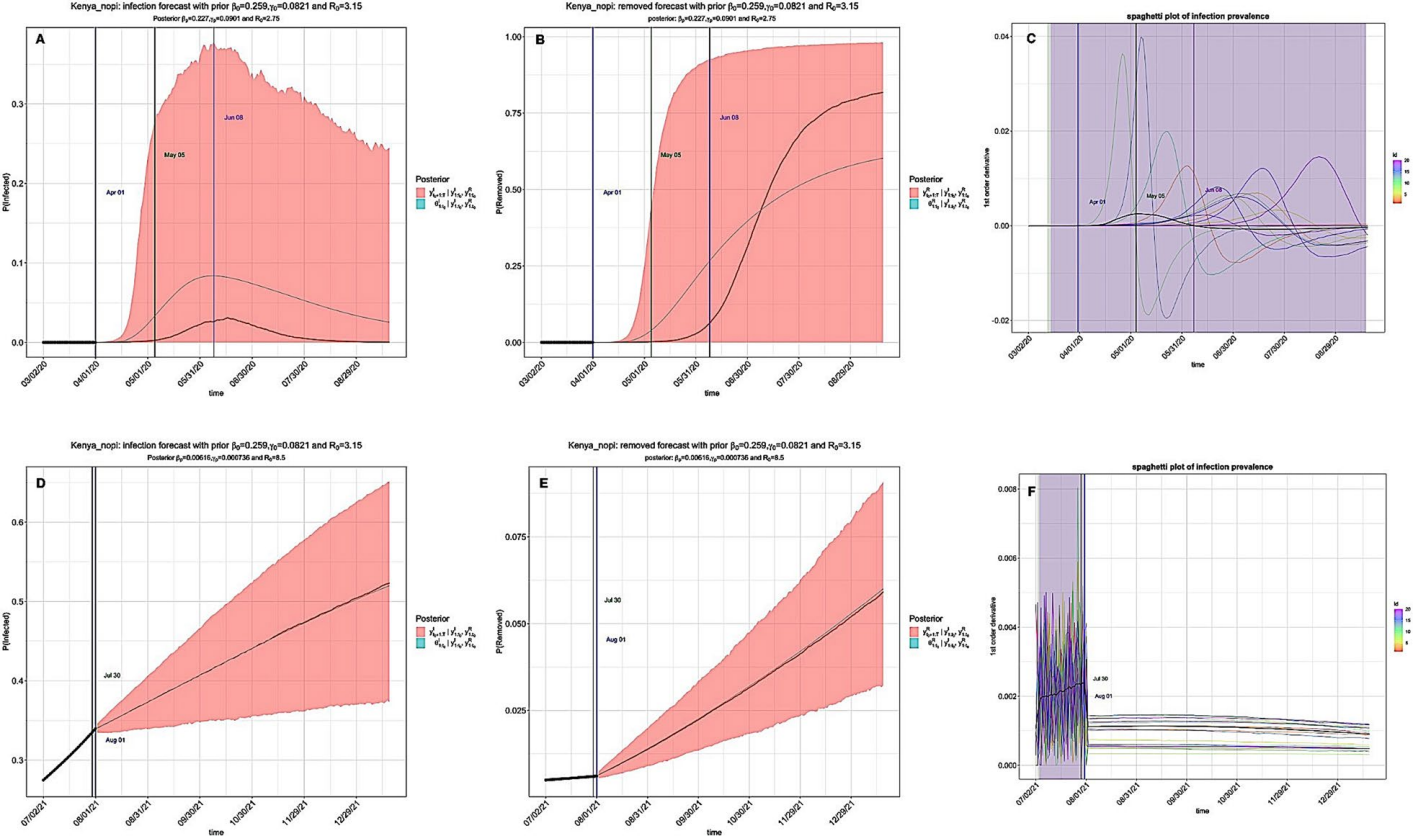
The standard SIR model without interventions in Kenya. The level of infection prevalence was high (R_0_ > 1) without intervention measures and, in particular, the endpoints were prolonged. **A** Prediction of the infection compartment during 2020/2021 window; The first and second turning points occurred on April 01 and May 05. **B** Prediction of the removed compartment during 2020/2021 window; **C** Plot of the first-order derivatives of the posterior prevalence of infection in 2020/2021. The black curve is the posterior mean of the derivative, and the vertical lines indicate the first and second turning points and the endpoint of the pandemic. The colored semi-transparent rectangles represent the 95% CI of these turning points. **D** Prediction of the infection compartment during 2021/2022 window; The first and second turning points occurred on July 30 and August 01. **E** Prediction of the removed compartment during 2021/2022 window; **F** Plot of the first-order derivatives of the posterior prevalence of infection for 2021/2022 time period

**Fig. 7:**
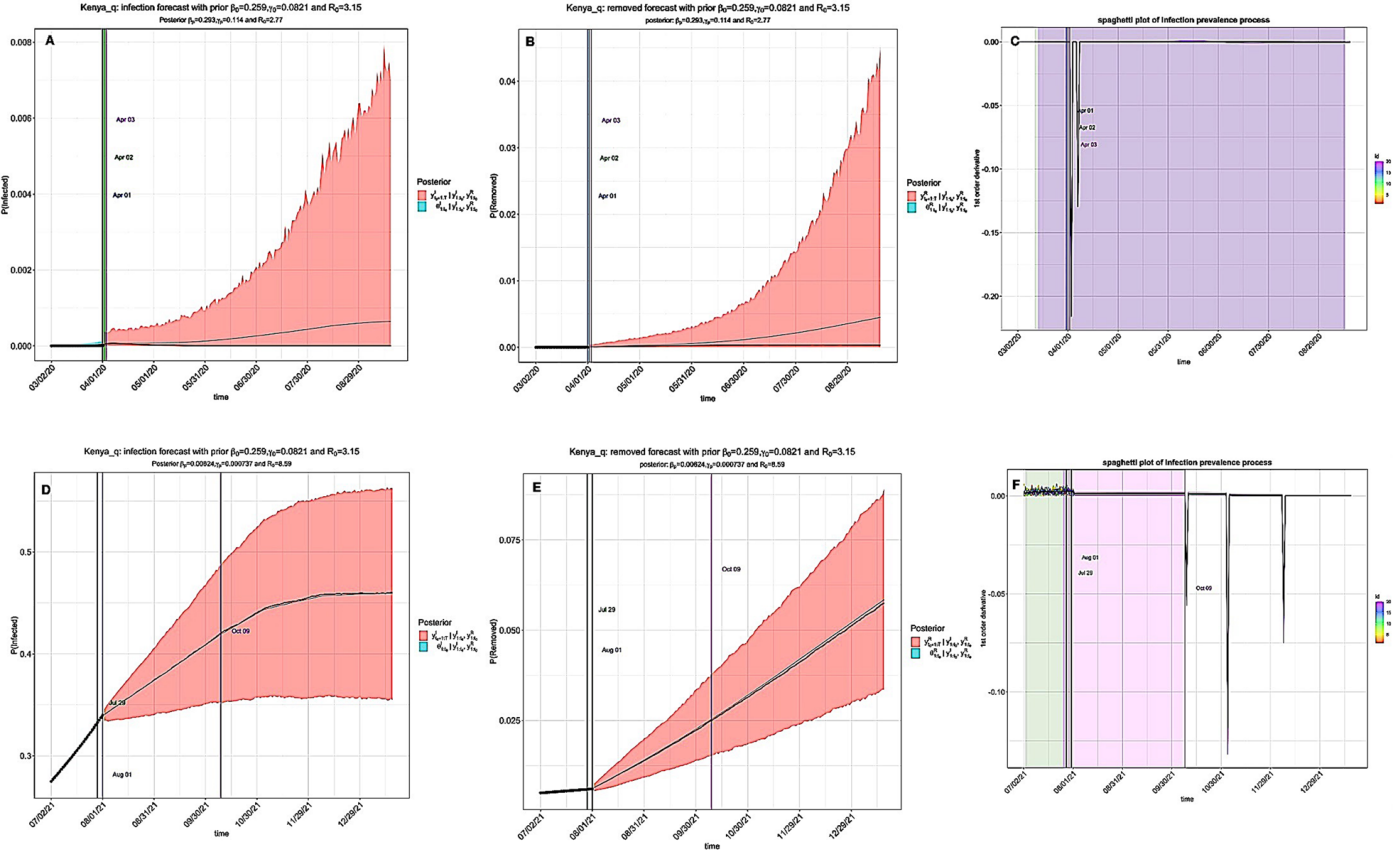
SIR model with time-varying quarantine. The level of infection prevalence remained high in Kenya (R_0_ = 8.59) during the 2020/2021 window. However, the end-point of the pandemic was projected to occur on October 09 2021. **A** Prediction of COVID-19 infection during 2020/2021 window. The first and second turning points occurred on April 01 and April 02 2020; **B** Prediction of the removed compartment during 2020/2021 window; **C** Plot of the first-order derivatives of the posterior prevalence of infection in 2020/2021. The colored semi-transparent rectangles represent the 95% CI of these turning points. **D** Prediction of the infection of COVID-19 for 2021/2022. The first and second turning points occurred on July 29 and August 01 2021; **E** Prediction of the removed compartment during 2021/2022 window; **F** Plot of the first-order derivatives of the posterior prevalence of infection

While NPIs had a substantial impact in mitigating the pandemic, simulation of the standard SIR model without interventions indicated rampant prevalence of the infection (R_0_ > 1) and the endpoints were prolonged (Fig. 6). On the contrary, simulation using SIR with time-varying quarantine produced a decline in time-varying reproduction number due to the introduction of quarantine measures (Tables 1 and 2). With time-varying R_0_ remaining above 1, most EACs are still under threat from the disease, with Kenya (R_0_ = 8.59) facing a higher risk (Fig. 7). However, the SEIR model estimated a lower R_0_ value (R_0_ ≤ 2.49) during the same time period in Kenya (Table 4). Further projections of cases-deaths-removed counts using the standard state-space SIR model without interventions are available as Additional file 2: Figs. S13–S18). Based on our results, we observed a decline in R_0_ and the infection prevalence during 2021/2022 time period in contrast with the 2020/2021 time period, in particular, when time varying quarantine measures were introduced into the model (Additional file 3: Figs. S19–S24).

### Epidemiological trends with a subset of the population having COVID-19 antibodies and the impact of vaccination campaigns

**Fig. 8:**
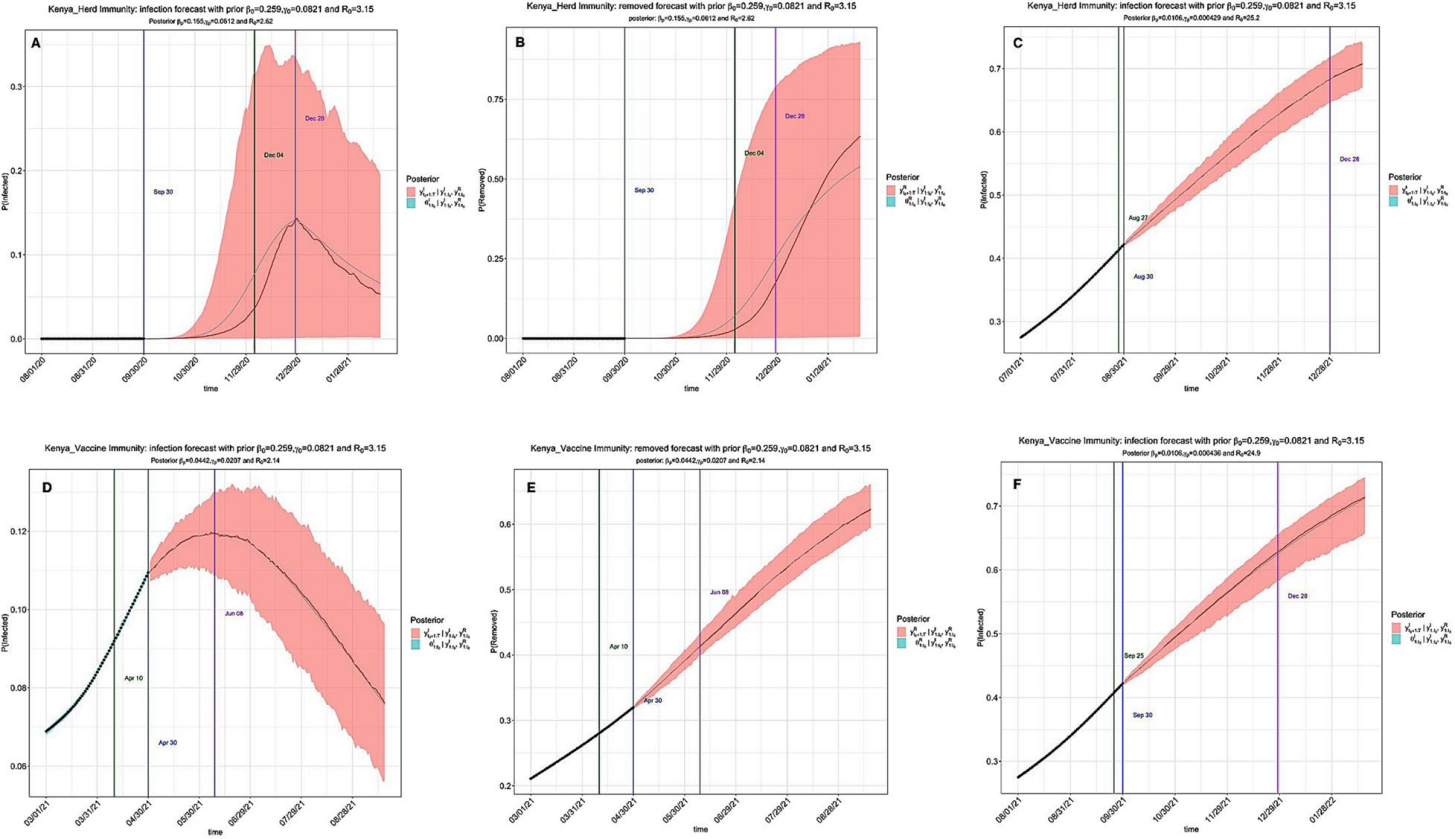
Estimation of herd immunity and vaccination campaign in Kenya. Simulation of herd and vaccine-derived immunity using time-varying SIR model with 20% of the population assumed to have acquired neutralizing antibodies and 2% of the population assumed to have been vaccinated against COVID-19. **A** Proportion of the infected compartment with antibodies against SARS-COV-2 during the 2020/2021 window. The first and second turning points occurred on September 30 and December 04 2020; **B** Proportion of the removed compartment with antibodies against SARS-COV-2 during 2020/2021 window. The first and second turning points occurred on September 30 and December 04 2020; **C** Prediction of the infection during 2021/2022 window assuming that 20% of the population has antibodies against SARS-COV-2. The first and second turning points occurred on August 27 and August 30 2021; **D** Prediction of infection during the 2020/2021 window assuming that 2% of the population is vaccinated. The first and second turning points occurred on April 10 and April 30 2021; **E** Prediction of the removed compartment assuming that 2% of the population is vaccinated. The first and second turning points occurred on April 10 and April 30 2021; **F** Prediction of infection in 2021/2022 assuming that 2% of the population is vaccinated. The first and second turning points occurred on September 25 and September 30 2021

Herd immunity was simulated using SIR model with a proportion of the population assumed to have neutralizing antibodies against COVID-19. We observed a decline in R_0_ under the assumption that 20% of the population in EACs had achieved herd immunity by 2021/2022. Furthermore, we also simulated the impact of vaccination on the dynamics of COVID-19. R_0_ declined from 8.52 to 2.14 under the assumption that 2% of the Kenyan population was vaccinated (Fig. 8). While vaccination eventually contributes to the achievement of herd immunity, our simulations showed that vaccination had a bigger impact than herd immunity in lowering the time varying reproduction number, R(t) (Additional file 3: Figs. S25–S30). Vaccination campaigns target to reduce the susceptible population and thereby lowering contacts between infectious and susceptible population [47].

### Validation of the model prediction accuracy

**Fig. 9:**
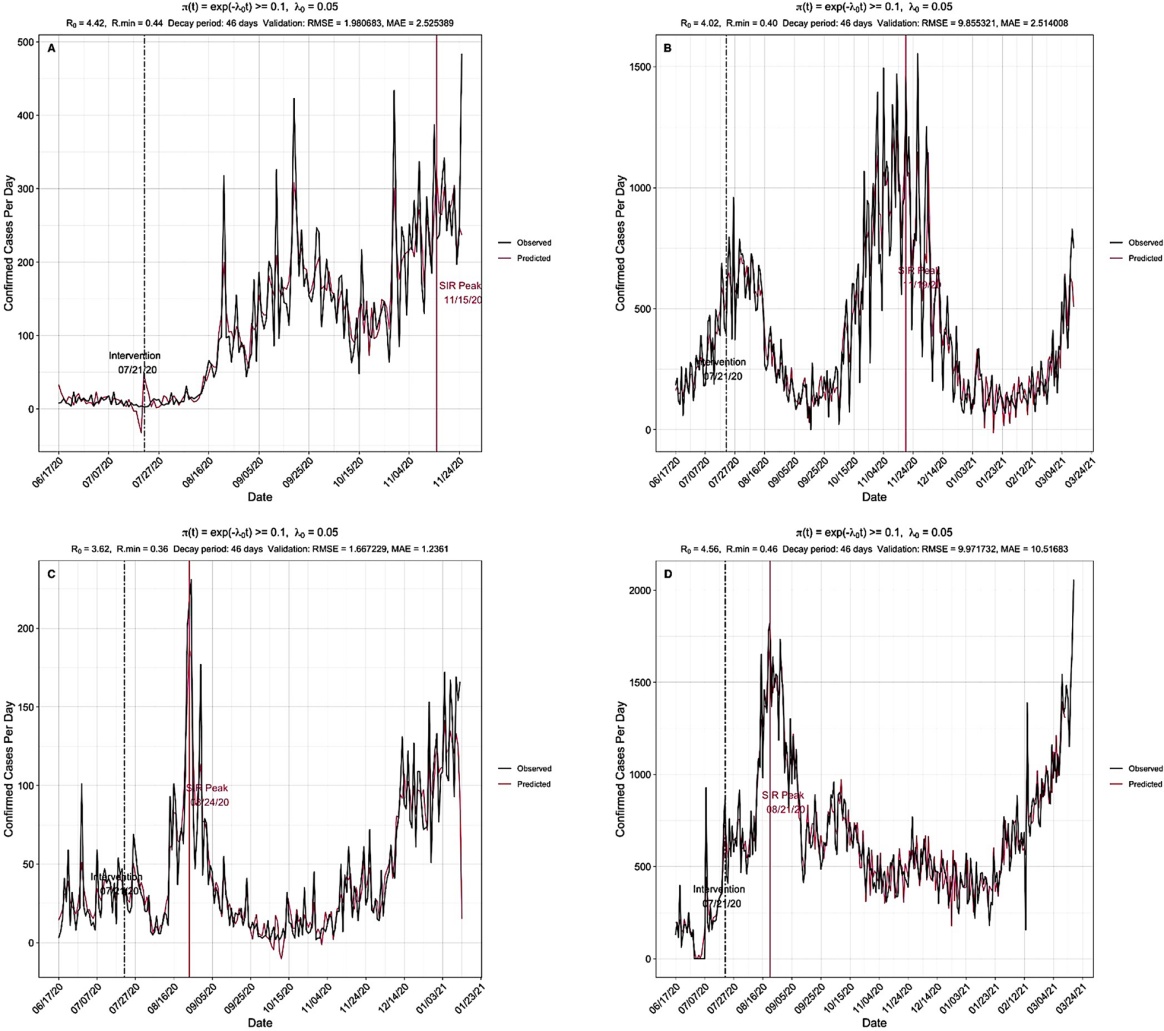
Validation of the robustness of the model for prediction COVID-19 case-death-recmoved counts. The predicted trends after the training period were compared to observed case-death-removed count and RMSE and MAE metrics computed. **A** Uganda; **B** Kenya; **C** Rwanda; **D** Ethiopia

A reliable model results in predicted values being close to the observed data values, which implies a good model fit [45]. We observed a good model fit between the forecasted cases and the actual observed cases of COVID-19 across four EACs (Table 3). Larger RMSE values indicate a wider difference between the predicted and observed values, which means poor regression model fit [43]. Similarly, the computed MAE values for the model ranged between 1.24 and 10.52 (Table 3). In general, lower RMSE and MAE values provide better support for the model fit (Fig. 9).

**Table 3:** Validation of the model prediction accuracy of the total number of COVID-19 cases

	Mean R_0_ (95% CI)	RMSE	MAE
Ethiopia	4.56 (2.90–6.45)	9.97	10.52
Kenya	4.02 (2.69–5.62)	9.86	2.51
Rwanda	3.62 (2.22–5.40)	1.67	1.24
Uganda	4.42 (2.47–7.13)	1.98	2.53

### Comparison of the eSIR and SEIR models

We generated estimates for the transmission of COVID-19 using a SEIR model extension implemented in the SEIR-fansy package [46]. The two models were compared with respect to the predicted R(t), active cases and deaths. SEIR model is considered superior to the standard SIR model because it takes into account the pre-symptomatic, asymptomatic and unreported cases as well as the high false negative rates of COVID-19 RT-PCR tests [46]. Moreover, the SIR model tends to overestimate the R_0_ because strict enforcement of NPIs causes the isolation of a large proportion of susceptible cases [48].

**Fig. 10:**
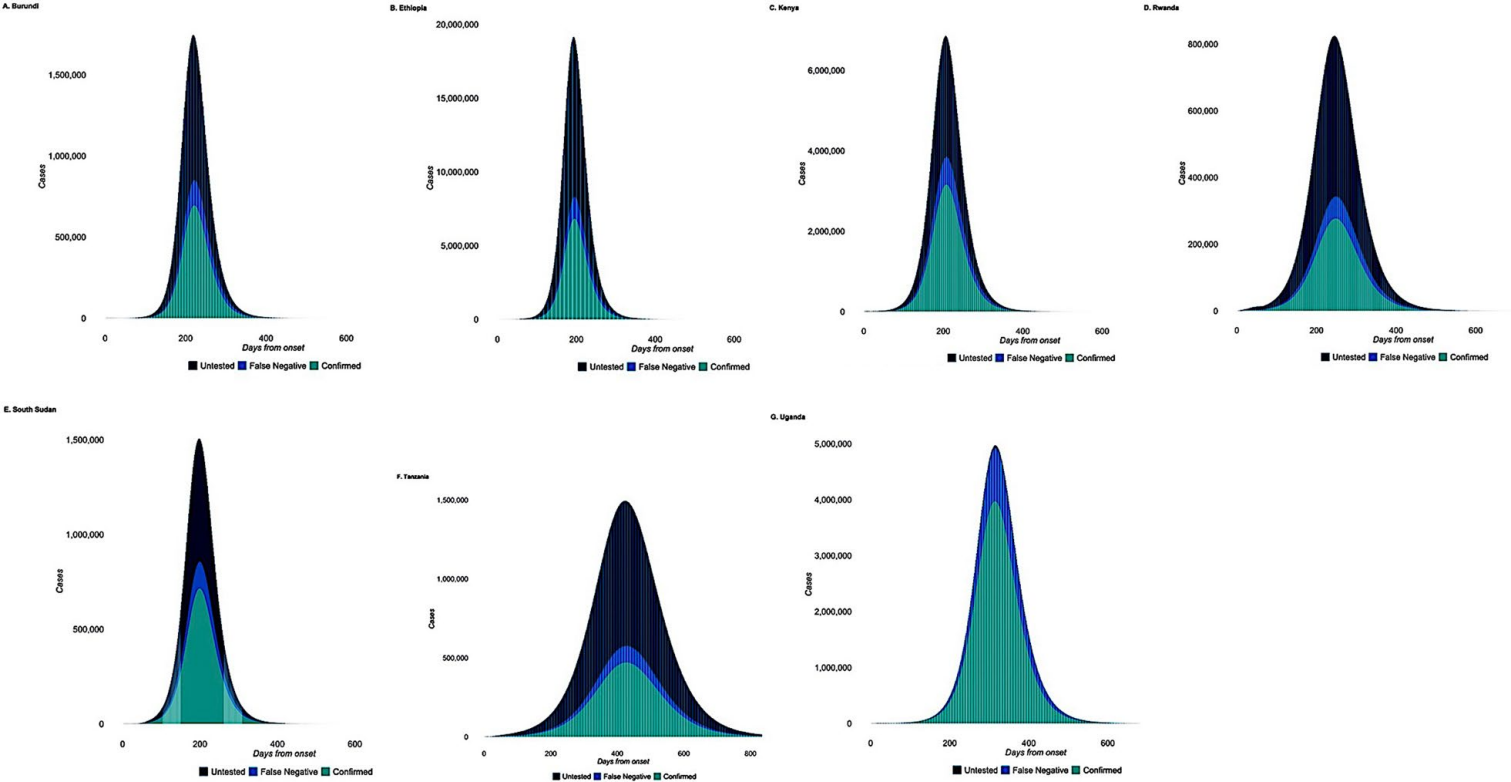
Effect of misclassification on predicted case counts. Estimated number of active cases including the false negative rates of tests, the unreported/untested case counts and confirmed cases across EACs

**Fig. 11:**
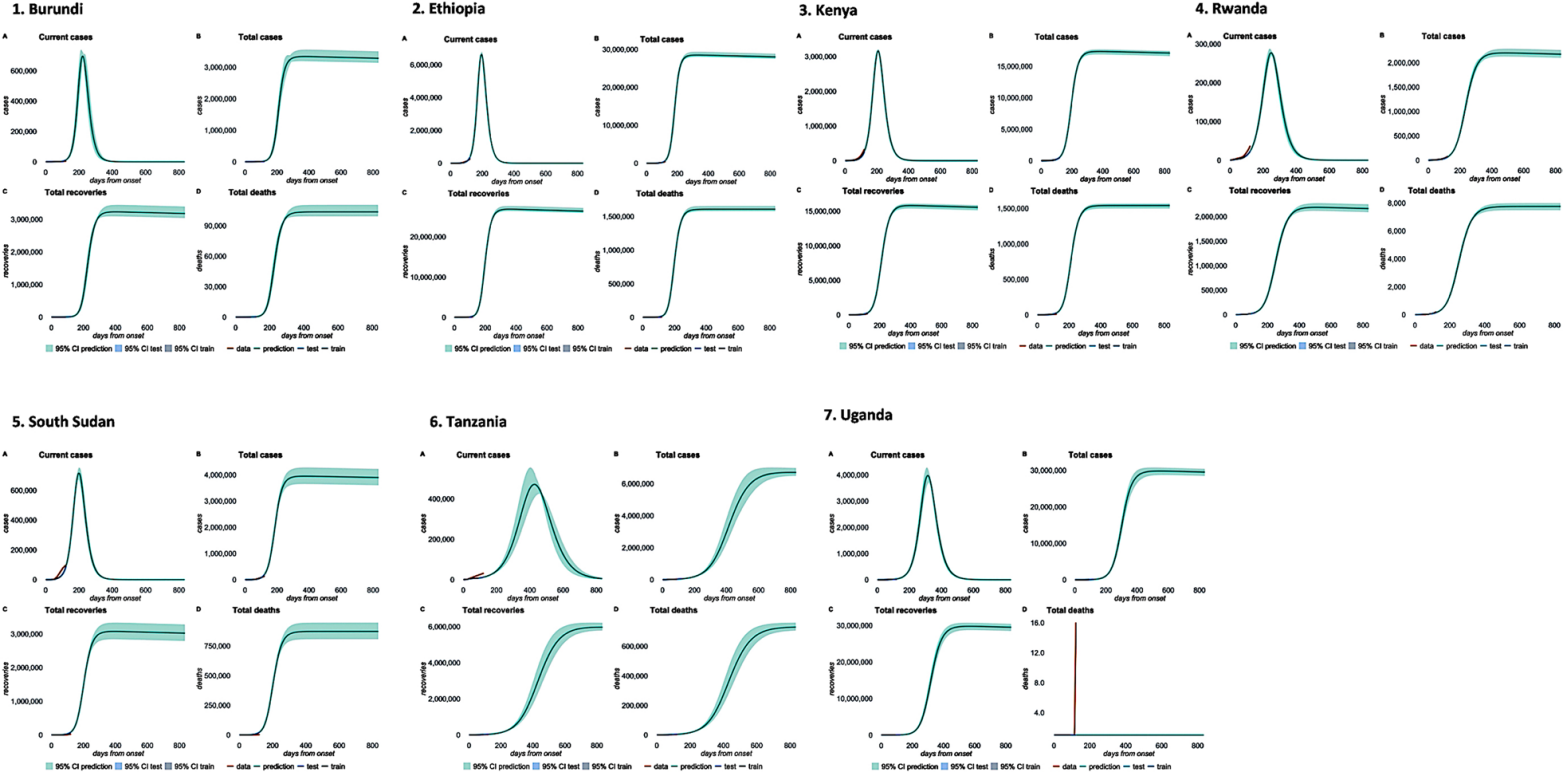
Predicted cases-deaths-recovery counts. There is a decline in active cases across EACs. The multinomial model of SEIR-fansy package estimated the peak of the pandemic to have occurred in July–August 2021. Predicted cases-deaths-recovery counts: 1. Burundi; 2. Ethiopia; 3. Kenya; 4. Rwanda; 5. South Sudan; 6. Tanzania; and 7. Uganda. In this figure: **A** = total number of current cases; **B** = cumulative number of confirmed cases; **C** = cumulative number of confirmed recoveries; **D** = cumulative number of confirmed deaths

Our findings show that the mean values returned by the SEIR model corroborate the eSIR model predictions except for wide margin of variability observed in R(t) estimates in Kenya, Rwanda and South Sudan (Table 4). Additionally, the SEIR model also considered the false positive/negative rates of tests, the unreported and untested case counts (Fig. 10). Uganda had the least number untested case counts and false negative rates in contrast to other EACs (Fig. 10G). We observed less variability in R(t) values projected by the SEIR model, in contrast to the eSIR model. For example, the eSIR model estimates of the R_0_ consistently remained above 1 across most EACs with Kenya (R_0_ = 8.59) facing a higher risk. However, the SEIR model projected a lower R_0_ value (R_0_ ≤ 2.49) during the same time period in Kenya (Additional file 3: Fig. S31 C). The two models projected the peak of the pandemic to have occurred between March–April and July–August 2021. Overall, we observed a decline in R(t) values. We anticipate that the COVID-19 curve has flattened for most EACs except in South Sudan and Tanzania where projected R(t) values remain high (Table 4 and Fig. 11).

**Table 4:** Comparison of estimated time-varying reproduction number (R_t_) obtained from eSIR and SEIR models for the 7 countries

Model Estimated mean reproduction number R_t_ (95% CI)				
Country	2020/2021		2021/2022	
	eSIR	SEIR-fansy	eSIR^1^	SEIR-fansy^1^
Burundi	2.71 (1.48–4.58)	2.57 (0.64–2.58)	2.84 (1.83–4.45)	2.54 (0.65–2.55)
Ethiopia	2.75 (1.57–4.65)	2.89 (1.65–2.90)	1.64 (1.39–1.99)	2.84 (1.62–2.85)
Kenya	2.70 (1.54–4.67)	2.51 (1.41–2.52)	8.52 (3.73–14.10)	2.49 (1.43–2.50)
Rwanda	3.10 (3.10–5.22)	2.03 (1.00–2.25)	1.32 (1.17–1.49)	2.08 (1.01–2.09)
South Sudan	2.71 (2.71–4.59)	4.41 (1.47–4.42)	1.54 (1.19–2.03)	4.60 (1.41–4.62)
Tanzania	2.82 (2.82–4.90)	3.25 (1.25–3.26)	2.57 (1.45–4.31)	3.27 (1.31–3.29)
Uganda	2.87 (2.87–4.79)	3.70 (0.58–3.71)	2.34 (1.67–3.33)	2.01 (0.78–2.03)

^1^Highlighted values are outliers due to overestimation by either model

### Parameter sensitivity analyses

We conducted parameter sensitivity analyses to evaluate the robustness of the model to prior settings. Parameter sensitivity analyses was performed using COVID-19 pandemic data between April 01 and June 30 2021. Initial parameters in the SEIR model were fixed and then followed by estimation of R_0_, β and γ parameters using the multinomial, Poisson and Binomial models (Table 5). We observed variations in R_0_ during the first phase (01–14 April) between the Multinomial, Poisson and Binomial models. However, the estimated R_0_ values were within the same range during later stages of the pandemic [27]. Our findings show that both models are robust enough to provide reliable predictions. However, the eSIR model tends to overestimate R_0_ values while the SEIR model has less variability in R_0_ estimates [27]. Previously, Ray et al. conducted an in-depth parameter sensitivity analyses using a range of scenarios in the context of the pandemic outbreak in India [49]. The reader is referred to Ray et al. for a detailed explanation of parameter sensitivity analyses [49].

**Table 5:** Comparison of posterior estimates of model parameters

Model	Posterior estimates														
	01–14 Apr			15 Apr–03 May			04–17 May			18–31 May			01–30 Jun		
	${R}_{0}$	$\beta$	$\gamma$	${R}_{0}$	$\beta$	$\gamma$	${R}_{0}$	$\beta$	$\gamma$	${R}_{0}$	$\beta$	$\gamma$	${R}_{0}$	$\beta$	$\gamma$
1. Multinomial															
Burundi	0.01	0.08	0.05	0.70	0.08	0.17	1.92	0.19	0.75	2.11	0.20	0.79	2.65	0.30	0.44
Ethiopia	3.00	0.34	0.29	1.72	0.19	0.32	2.59	0.30	0.26	2.95	0.34	0.23	2.55	0.29	0.35
Kenya	0.52	0.06	0.18	1.50	0.16	0.40	2.00	0.21	0.55	2.58	0.27	0.43	2.18	0.23	0.45
Rwanda	4.85	0.56	0.32	2.11	0.25	0.23	1.33	0.16	0.27	1.14	0.13	0.33	1.72	0.20	0.31
South Sudan	0.65	0.07	0.17	1.48	0.15	0.33	4.64	0.48	0.45	3.97	0.40	0.53	2.19	0.22	0.55
Tanzania	0.50	0.05	0.59	3.28	0.32	0.77	1.71	0.17	0.60	1.73	0.18	0.42	1.35	0.15	0.30
Uganda	2.29	0.29	0.08	0.87	0.11	0.16	1.13	0.13	0.35	1.54	0.16	0.61	2.00	0.18	0.99
2. Poisson															
Burundi	7.92	0.94	0.20	6.31	0.75	0.20	4.9	0.59	0.19	3.30	0.39	0.23	1.66	0.20	0.19
Ethiopia	8.90	0.95	0.19	6.56	0.70	0.21	5.10	0.54	0.21	3.67	0.39	0.25	1.82	0.19	0.20
Kenya	8.30	0.96	0.20	6.72	0.78	0.20	4.93	0.57	0.18	3.35	0.38	0.22	1.75	0.20	0.19
Rwanda	7.71	0.93	0.20	6.17	0.75	0.21	4.73	0.57	0.20	3.16	0.38	0.23	1.56	0.19	0.19
South Sudan	8.95	0.95	0.21	6.95	0.74	0.22	5.23	0.56	0.20	3.62	0.38	0.24	1.82	0.19	0.19
Tanzania	8.77	0.93	0.21	6.99	0.74	0.21	5.28	0.56	0.21	3.53	0.37	0.24	1.82	0.19	0.19
Uganda	9.01	0.96	0.21	6.96	0.74	0.21	5.17	0.55	0.20	3.65	0.39	0.23	1.83	0.19	0.19
3. Binomial															
Burundi	7.99	0.95	0.21	6.26	0.75	0.21	4.78	0.57	0.20	3.23	0.38	0.23	1.61	0.19	0.19
Ethiopia	8.91	0.95	0.21	7.01	0.74	0.20	5.28	0.56	0.20	3.54	0.37	0.25	1.81	0.19	0.19
Kenya	7.99	0.93	0.20	6.68	0.77	0.20	5.02	0.58	0.19	3.34	0.38	0.23	1.71	0.20	0.20
Rwanda	7.66	0.93	0.20	5.99	0.73	0.20	4.69	0.57	0.20	3.17	0.38	0.23	1.59	0.19	0.19
South Sudan	8.93	0.95	0.21	6.99	0.74	0.20	5.22	0.55	0.20	3.72	0.39	0.23	1.83	0.19	0.19
Tanzania	8.82	0.94	0.21	6.83	0.72	0.20	5.23	0.56	0.19	3.56	0.38	0.24	1.90	0.20	0.19
Uganda	8.63	0.92	0.21	6.85	0.73	0.20	5.22	0.55	0.20	3.77	0.40	0.22	1.78	0.19	0.19

$\beta$ = rate of transmission of infection by false negative individuals

$$\gamma$$ = recovery rate

## Discussion

Following the first reported case of COVID-19 in Egypt, the number of cases gradually increased across the continent causing human and economic losses. However, fatalities have remained low particularly during the initial phases of the pandemic. Several arguments to this observation have been put forward including experience with previous pandemics (Ebola virus disease, human immunodeficiency virus, polio, and tuberculosis), demographic factors, host genetics factors, climate and environmental factors [7]. Beyond health risks, the socio-economic implications of the pandemic motivated many countries to implement NPIs such as wearing masks, lockdown of cities, stop transports, school closure, social distancing, and hand washing [13].

In this study, we applied the eSIR compartmental model to project epidemiological trends of COVID-19 and the impact of NPIs in seven EACs [25, 26]. Publicly available data from JHU as at 30th July 2021 were used to estimate the transmission rate of the epidemic and to present the trend of infections and fatalities following government interventions [40]. Parameters such as the R_0_ and R(t) are of great importance for policy makers to adopt the most efficient and effective interventions in order to contain the pandemic and minimize human and economic damages [50].

Our findings show that the epidemic trend of COVID-19 differs among EACs with infections remaining high while fatalities are low [51]. The R_0_ posterior values and endpoints in EACs during the 2020/2021 and 2021/2022 window provided a snapshot of the trajectories of the disease. However, foreseen risks include under-estimation of the disease extend due to asymptomatic cases and unreported cases as well as the high false negative rates of COVID-19 RT-PCR tests [46]. To circumvent these risks, we applied a SEIR model implemented in the SEIR-fansy package to account for the presymptomatic and asymptomatic infection and transmission of COVID-19 [46]. We found that interventions that were implemented during the initial stages of the pandemic had a strong impact on reducing the transmission of the disease. For example, after calibrating the model using time-series data from March 02/2020 to May 01/2020, our predictions revealed a modest R_0_ value of 2.71 (95% CI: 1.48–4.58), 2.75 (95% CI: 1.57–4.65), 2.70 (95% CI: 1.54–4.67), 3.10 (95% CI: 3.10–5.22), 2.71 (95% CI: 2.71–4.59), 2.82 (95% CI: 2.82–4.90), 2.87 (95% CI: 2.87–4.79) for Burundi, Ethiopia, Kenya, Rwanda, South Sudan, Tanzania, and Uganda respectively. However, R_0_ marginally decreased under the same time period in 2021/2022 projections, except in Burundi and Kenya where the value increased to a mean of 2.84 and 8.52 respectively. Previous studies of the pandemic in Kenya, reported a range of R_0_ values between 1.78 (95% CI: 1.44–2.14) to 3.46 (95% CI: 2.81–4.17) [52–54,52–54]. Indeed, our findings (R_0_ = 2.70, CI: 1.54–4.67) lie within this range.

The exponential model mimicking increased community awareness of NPIs, had more impact in lowering the transmission rate of the disease than the stepwise model that mimics governmental interventions at specific timepoints. As the pandemic evolves, the public perceptions and attitudes towards the interventions change and strict adherence to public policies is practiced [13]. Moreover, the time point of implementation of NPIs is key to their success in reducing the peak of the epidemic [12]. Overall, the 2021/2022 epidemic trajectories indicate that EACs are facing challenges in their efforts to contain community transmission of COVID-19. Country-specific mean R_0_ values remain above 2 (R_0_ > 2) with the exception of Ethiopia, Rwanda and South Sudan. This is further compounded by the weak health systems, inadequate preparedness and capacity to respond to emerging epidemics. Based on these results, we suggest strict implementation of intervention policies, such as enforcement of lockdowns, face-mask wearing, long-term surveillance and COVID-19 vaccine roll-out to contain the pandemic. However, we recommend careful interpretation of the R_0_ values because of the unforeseen risks such as under-estimation of the disease extend due to asymptomatic cases and low testing rate which is not randomized.

Under the current intervention measures, the long-term projection of the eSIR exponential model indicates that about 0.97, 6.15, 33.94, 3.17, 3.45, 0.18, 6.88% of the population will be infected by 16th January 2022 in Burundi, Ethiopia, Kenya, Rwanda, South Sudan, Tanzania, and Uganda respectively. The high number of recorded cases of COVID-19 could be attributed to the weak health infrastructure, crowded social life and poor personal hygiene. Moreover, disease comorbidities like hypertension, obesity, type II diabetes, HIV, tuberculosis and malaria are highly prevalent in Africa and may contribute to the weak immune response to COVID-19 [7, 55, 56,7, 55, 56]. The comorbid individuals must be prioritized in terms of healthcare and vaccine roll-out.

Previous predictive models suggested that Africa could be the next hotspot of COVID-19, yet to-date, recorded cases and deaths have remained low. Multiple factors have been attributed to the low COVID-19 reported cases and fatalities in Africa including herd immunity due to antibodies against SARS-COV-2, climate, comorbidities, parasite exposure, and young population structure [51, 57, 58]. Indeed, a recent study by the WHO revealed that over two-thirds (65% or 800 million infections) of Africans were exposed to SARS-COV-2 virus by September 2021 against a backdrop of 8.2 million reported cases [59]. Seroprevalence varied between countries, being highest in Eastern, Western and Central African regions. Currently, the reported seroprevalence of antibodies against SARS-CoV-2 range from 0.4% in Cape Verde and 49% in antenatal care clinics in Kenya [60, 61]. Despite these reports, most of these factors attributed to low mortalities have not been studied conclusively to establish their interaction with COVID-19 [7]. Multiple studies have associated the low mortality rates of COVID-19 in Africa to host immunity [51]. For example, the “trained immunity” hypothesis suggests that the Bacillus Calmette-Guérin (BCG) vaccine against tuberculosis confers protection against COVID-19 [51]. Brewster et al. documented that Africans have genetic mutations in the SARS-CoV-2 receptor, angiotensin-converting enzyme-2 (ACE-2) gene, which confers low response to ACE inhibitors and therefore linked to low prevalence of COVID-19 [62]. Furthermore, previous exposure to *Plasmodium falciparum* and other pathogens is associated with protective immunity and has been linked to a lower prevalence of COVID-19 in malaria-endemic areas [57, 63]. Additionally, the demographic structure of Africa’s population that has a predominantly young population aged below 35 years, and with few comorbidities has been linked to low prevalence to COVID-19. However, such population can be super spreaders of the virus because they are largely asymptomatic [64].

We estimated the herd effect due to genetic factors and COVID-19 vaccination campaigns by incorporating assumptions (about the percentage of the population with anti-SARS-CoV-2 antibodies and the percentage of the population that had been vaccinated) into the simulation of infection dynamics. By assuming that about 20% of the population in each country had neutralizing antibodies against COVID-19, we observed a significant decline in R_0_ from 8.52 to 2.62 by January 16th 2021/2022 in all the EACs. Similarly, R_0_ declined from 8.52 to 2.14 under the assumption that 2% of the Kenyan population is vaccinated. While vaccination eventually contributes to the achievement of herd immunity, vaccination had a bigger impact than herd immunity in lowering R_0_ and hence the number of cases and deaths.

During the initial phases of pandemic, the entire African population had no immunity against COVID-19, hence the virus spread quickly across communities. However, as the disease evolved, gradual immunity developed aided by genetic factors, previous parasite exposure, and a young population structure with few underlying comorbidities. The COVID-19 vaccine has been rolled-out in Africa with 49 countries having administered at least one dose. However, the vaccination coverage required to establish herd immunity against COVID-19 is quite heterogeneous, ranging from 0, 2.0, 2.2, 3.5, 0.46, 0.18 and 2.5% of the population having received at least one dose of the vaccine in Burundi, Ethiopia, Kenya, Rwanda, South Sudan, Tanzania, and Uganda respectively as of 12th August 2021 [65, 66]. Flattening the curve requires a significant percentage of population to be immunized. In particular, we recommend that countries with high basic reproduction number (R_0_ > 1) such as Kenya (8.52), Burundi (2.84), Uganda (2.34) and Tanzania (2.57) should increase vaccine coverage required to establish herd immunity against COVID-19 and strictly enforce interventions. However, the current situation is further complicated by weak health systems in EACs, the inequitable vaccine distribution, vaccine hesitancy and negative perception of government interventions. Furthermore, the emergence of COVID-19 variants, such as B.1.617 (“Delta”) and BA.2 Omicron variants, has led to upsurge of cases due to declining protective immunity or the circulation of immune escape viral variants [7, 67–69].

Epidemiological models for projecting infectious disease spread have been used to inform public health policy [22, 70–72]. To evaluate the reliability and usefulness of our model, we compared model predictions of the case-counts against the observed data for COVID-19 in Ethiopia, Kenya, Rwanda and Uganda using the Root Mean square error (RMSE) and Mean Absolute Error (MAE). The metrics provided good support to the model fit to the observed COVID-19 cases with larger values indicative of a wider difference between the predicted and observed values, hence poor model fit. The modelling techniques that we used in this study to characterize the epidemic dynamics has been successfully applied to the data in India and Wuhan, China, separately [25–27]. A reliable model results in predicted values close to the observed data values [73, 74].

The original eSIR epidemiology model does not provide validation of the predictions [25]. One of the novel contributions to the model was to validate the predictions made by the model using subsequent data from Ethiopia, Kenya, Rwanda and Uganda. A second additional strength was the incorporation of a vaccination compartment into the model to account for vaccine-induced immunity over time. However, we acknowledge that some aspects of these analyses have limitations. For example, the model did not account for under estimation of the reported cases, asymptomatic cases, the population structure, health systems, climate and environmental factors that can affect predictions and forecasts [14, 17, 75, 76].

## Conclusions

The current intervention measures can efficaciously prevent the further spread of COVID-19 and should be strengthened. However, the impact of these interventions is highly heterogeneous across EACs. Close collaboration between regional governments, the scientific community, and health care providers is required to manage the pandemic. Moreover, comparison of the basic reproduction number (R_0_) between countries should take into consideration the under estimation of the reported cases, asymptomatic cases, demographic factors such as the population structure, health systems, host genetics factors, climate and environmental factors. The observed reduction in R_0_ is consistent with intervention measures implemented in EACs, in particular, lockdowns and roll-out of vaccination programmes. Future work should account for the negative impact of the interventions to the economy and food systems.

## Supplementary Information

**Additional file 1.** Supplementary figures. Scenario analysis of COVID-19 pandemic using the exponential model (Figure S1 - S6) and the stepwise model (Figure S7 - S12) in Burundi, Ethiopia, Rwanda, South Sudan, Tanzania, and Uganda respectively.

**Additional file 2.** Supplementary figures. Projections of COVID-19 epidemic trends in Burundi, Ethiopia, Rwanda, South Sudan, Tanzania, and Uganda using the standard state-space SIR model without interventions (Figure S13 – S18).

**Additional file 3.** Supplementary figures. Estimation of COVID-19 epidemic trends using a time-varying quarantine model (Figure S19 - S24) and scenario projection of herd immunity and vaccination campaign in Burundi, Ethiopia, Rwanda, South Sudan, Tanzania, and Uganda respectively (Figure S25 - S30). Further estimates of R0 values across using the multinomial-2-parameter SEIR model (Figure S31).

## Data Availability

The data of cumulative number of COVID-19 infected cases are available from COVID-19 Data Repository by the Johns Hopkins University Center for Systems Science and Engineering (JHU CCSE) at https://github.com/CSSEGISandData/COVID-19. The R packages used in tjis study are publicly available at https://github.com/lilywang1988/eSIR and https://github.com/umich-biostatistics/SEIRfansy.

## Abbreviations

CI: Confidence interval
Beta (β): The disease transmission rate
Gamma (γ): The disease removal rate
WHO: World Health Organization
RT-PCR: Reverse transcription polymerase chain reaction
COVID-19: Coronavirus disease 2019
SARS-CoV-2: Severe Acute Respiratory Syndrome Coronavirus 2
NPIs: Non-pharmaceutical intervention(s)
EACs: Eastern Africa countries
SIR: Susceptible, infected, recovered
eSIR: Extended susceptible, infected, removed
SAIR: Susceptible, antibody, infected and removed
SEIR: Susceptible, exposed, infected, recovered
R_0_: The basic reproduction number
R(t): Time-varying reproduction number
MCMC: Markov chain Monte Carlo
RMSE: Root Mean square error
MAE: Mean Absolute Error
JHU: Johns Hopkins University
IHME: Institute of Health Metrics, Washington, Seattle
ICL: Imperial College London

## References

[1] Gorbalenya AE, Baker SC, Baric RS (2020). The species Severe acute respiratory syndrome-related coronavirus: classifying 2019-nCoV and naming it SARS-CoV-2. Nat Microbiol.

[2] Hui DS, Azhar EI, Madani TA (2020). The continuing 2019-nCoV epidemic threat of novel coronaviruses to global health—the latest 2019 novel coronavirus outbreak in Wuhan, China. Int J Infect Dis.

[3] Lu H, Stratton CW, Tang YW (2020). Outbreak of pneumonia of unknown etiology in Wuhan, China: the mystery and the miracle. J Med Virol.

[4] Fauci AS, Lane HC, Redfield RR (2020). COVID-19—navigating the uncharted. N Engl J Med.

[5] Wang C, Horby PW, Hayden FG (2020). A novel coronavirus outbreak of global health concern. The Lancet.

[6] Africa CDC (2020). Coronavirus Disease 2019 (COVID-19)—Africa CDC. Africa CDC Dashboard.

[7] Tessema SK, Nkengasong JN (2021). Understanding COVID-19 in Africa. Nat Rev Immunol.

[8] Mboera LEG, Akipede GO, Banerjee A (2020). Mitigating lockdown challenges in response to COVID-19 in Sub-Saharan Africa. Int J Infect Dis.

[9] Gilbert M, Pullano G, Pinotti F (2020). Preparedness and vulnerability of African countries against importations of COVID-19: a modelling study. Lancet.

[10] Hagan JE, Ahinkorah BO, Seidu AA (2020). Africa’s COVID-19 situation in focus and recent happenings: a mini review. Front Public Health.

[11] Wu D, Lu J, Liu Y (2020). Positive effects of COVID-19 control measures on influenza prevention. Int J Infect Dis.

[12] Imai N, Gaythorpe KAM, Abbott S (2020). Adoption and impact of non-pharmaceutical interventions for COVID-19. Wellcome Open Res.

[13] Doogan C, Buntine W, Linger H (2020). Public perceptions and attitudes toward COVID-19 nonpharmaceutical interventions across six countries: a topic modeling analysis of twitter data. J Med Internet Res.

[14] Flaxman S, Mishra S, Gandy A, et al. Estimating the effects of non-pharmaceutical interventions on COVID-19 in Europe. Nature. 2020; 1–8. 10.1038/s41586-020-2405-7 32512579

[15] Chaudhry R, Dranitsaris G, Mubashir T (2020). A country level analysis measuring the impact of government actions, country preparedness and socioeconomic factors on COVID-19 mortality and related health outcomes. EClinicalMedicine.

[16] Jacobi L, Joshi M, Zhu D (2018). Automated sensitivity analysis for Bayesian inference via Markov Chain Monte Carlo: applications to Gibbs sampling. SSRN Electron J.

[17] Murray CJ (2020). Forecasting COVID-19 impact on hospital bed-days, ICU-days, ventilator-days and deaths by US state in the next 4 months. MedRxiv.

[18] Delamater PL, Street EJ, Leslie TF (2019). Complexity of the basic reproduction number (R0). Emerg Infect Dis.

[19] Shetty RM, Achaiah NC, Subbarajasetty SB (2020). R0 and re of COVID-19: can we predict when the pandemic outbreak will be contained?. Indian J Crit Care Med.

[20] Osthus D, Hickmann KS, Caragea PC (2017). Forecasting seasonal influenza with a state-space SIR model. Ann Appl Stat.

[21] Baroyan OV, Rvachev LA, Basilevsky UV (1971). Computer modelling of influenza epidemics for the whole country (USSR). Adv Appl Probab.

[22] Coburn BJ, Wagner BG, Blower S (2009). Modeling influenza epidemics and pandemics: insights into the future of swine flu (H1N1). BMC Med.

[23] Mbuvha R, Marwala T (2020). Bayesian inference of COVID-19 spreading rates in South Africa. PLoS ONE.

[24] Kermack WO, McKendrick AG (1991). Contributions to the mathematical theory of epidemics-I. Bull Math Biol.

[25] Wang L, Zhou Y, He J (2021). An epidemiological forecast model and software assessing interventions on the COVID-19 epidemic in China. J Data Sci.

[26] Wangping J, Ke H, Yang S (2020). Extended SIR prediction of the epidemics trend of COVID-19 in Italy and compared With Hunan, China. Front Med.

[27] Purkayastha S, Bhattacharyya R, Bhaduri R (2021). A comparison of five epidemiological models for transmission of SARS-CoV-2 in India. BMC Infect Dis.

[28] Butcher JC. Runge–Kutta Methods. In: Numerical Methods for Ordinary Differential Equations. John Wiley & Sons, Ltd, 2008, pp. 137–316.

[29] Yu X, Dai Q (2006). The Runge-Kutta DG finite element method and the KFVS scheme for compressible flow simulations. Numer Methods Partial Differ Equ.

[30] Mkhatshwa T, Mummert A (2011). Modeling super-spreading events for infectious diseases: case study SARS. IAENG Int J Appl Math.

[31] Choi Y, Chan AP (2015). PROVEAN web server: a tool to predict the functional effect of amino acid substitutions and indels. Bioinformatics.

[32] Johansson MA, Quandelacy TM, Kada S (2021). SARS-CoV-2 transmission from people without COVID-19 symptoms. JAMA Netw Open.

[33] Lourenço J, Paton R, Ghafari M, et al. Fundamental principles of epidemic spread highlight the immediate need for large-scale serological surveys to assess the stage of the SARS-CoV-2 epidemic. medRxiv. 2020; 2020.03.24.20042291.

[34] Li R, Pei S, Chen B (2020). Substantial undocumented infection facilitates the rapid dissemination of novel coronavirus (SARS-CoV-2). Science.

[35] Yuan HY, Han G, Yuan H, et al. The importance of the timing of quarantine measures before symptom onset to prevent COVID-19 outbreaks—illustrated by Hong Kong’s intervention model. medRxiv. 2020; 2020.05.03.20089482.

[36] Mizumoto K, Kagaya K, Zarebski A (2020). Estimating the asymptomatic proportion of coronavirus disease 2019 (COVID-19) cases on board the Diamond Princess cruise ship, Yokohama, Japan, 2020. Eurosurveillance.

[37] Subramanian R, He Q, Pascual M (2021). Quantifying asymptomatic infection and transmission of COVID-19 in New York City using observed cases, serology, and testing capacity. Proc Natl Acad Sci U S A.

[38] Elizabeth Halloran M, Levin BR (1993). Infectious diseases of humans: dynamics and control (pbk edn). Trends Microbiol.

[39] World Health Organization. WHO Coronavirus (COVID-19) Dashboard. Who. 2021; 1–5.

[40] Johns Hopkins University. COVID-19 Map—Johns Hopkins Coronavirus Resource Center. Johns Hopkins Coronavirus Resource Center. 2020; 1.

[41] Plummer M, Stukalov A, Denwood M. Bayesian Graphical Models using MCMC—package ‘rjags’. Comprehensive R Archive Network (CRAN), 2019.

[42] Verity R, Okell LC, Dorigatti I (2020). Estimates of the severity of coronavirus disease 2019: a model-based analysis. Lancet Infect Dis.

[43] Grace-Martin K. Assessing the fit of regression models. The Analysis Factor. 2016; 1–13.

[44] Gholamy A, Kreinovich V, Kosheleva O. Why 70/30 or 80/20 relation between training and testing sets: a pedagogical explanation. Dep Tech Reports. 2018; 1–6.

[45] Batool H, Tian L (2021). Correlation determination between COVID-19 and weather parameters using time series forecasting: a case study in Pakistan. Math Probl Eng.

[46] Bhaduri R, Kundu R, Purkayastha S, et al. Extending the Susceptible-Exposed-Infected-Removed(SEIR) Model to handle the high false negative rate and symptom-based administration of COVID-19 diagnostic tests: SEIR-fansy. medRxiv Prepr Serv Heal Sci 2020; 2020.09.24.20200238. 10.1002/sim.9357 PMC9035093 35224743

[47] Achaiah NC, Subbarajasetty SB, Shetty RM (2020). R0 and re of COVID-19: can we predict when the pandemic outbreak will be contained?. Indian J Crit Care Med.

[48] You C, Deng Y, Hu W (2020). Estimation of the time-varying reproduction number of COVID-19 outbreak in China. Int J Hyg Environ Health.

[49] Ray D, Salvatore M, Bhattacharyya R (2020). Predictions, role of interventions and effects of a historic national lockdown in India’s response to the COVID-19 Pandemic: data science call to arms. Harvard Data Sci Rev..

[50] Taghizadeh L, Karimi A, Heitzinger C (2020). Uncertainty quantification in epidemiological models for the COVID-19 pandemic. Comput Biol Med.

[51] Ghosh D, Jonathan A, Mersha TB (2020). COVID-19 pandemic: the African paradox. J Glob Health.

[52] Quaife M, Van Zandvoort K, Gimma A (2020). The impact of COVID-19 control measures on social contacts and transmission in Kenyan informal settlements. BMC Med.

[53] Brand SPC, Aziza R, Kombe IK, et al. Forecasting the scale of the COVID-19 epidemic in Kenya. medRxiv. 2020; 2020.04.09.20059865.

[54] Mwalili S, Kimathi M, Ojiambo V, et al. Age-structured impact of mitigation strategies on COVID-19 severity and deaths in Kenya. ResearchSquare 2020; 1–14.

[55] Das S, Anu KR, Birangal SR (2020). Role of comorbidities like diabetes on severe acute respiratory syndrome coronavirus-2: a review. Life Sci.

[56] Ejaz H, Alsrhani A, Zafar A (2020). COVID-19 and comorbidities: deleterious impact on infected patients. J Infect Public Health.

[57] Iesa MAM, Osman MEM, Hassan MA (2020). SARS-CoV-2 and *Plasmodium falciparum *common immunodominant regions may explain low COVID-19 incidence in the malaria-endemic belt. New Microbes New Infect.

[58] Kong JD, Tekwa EW, Gignoux-Wolfsohn SA (2021). Social, economic, and environmental factors influencing the basic reproduction number of COVID-19 across countries. PLoS ONE.

[59] WHO Africa. Over two-thirds of Africans exposed to virus which causes COVID-19: WHO study | WHO | Regional Office for Africa, https://www.afro.who.int/news/over-two-thirds-africans-exposed-virus-which-causes-COVID-19-who-study (accessed 8 April 2022).

[60] Gomez LF, Mendes C, Silva JS (2021). Sero-epidemiological survey and profile of SARS-CoV-2 infection in cape verde. SSRN Electron J.

[61] Lucinde R, Mugo D, Bottomley C, et al. Sero-surveillance for IgG to SARS-CoV-2 at antenatal care clinics in two Kenyan referral hospitals Corresponding author + Contributed equally KEMRI-Wellcome Trust Research Programme. *medRxiv* 2021; 2021.02.05.21250735.

[62] Brewster LM, Seedat YK (2013). Why do hypertensive patients of African ancestry respond better to calcium blockers and diuretics than to ACE inhibitors and β-adrenergic blockers? A systematic review. BMC Med.

[63] Anjorin AA, Abioye AI, Asowata OE (2021). Comorbidities and the COVID-19 pandemic dynamics in Africa. Trop Med Int Heal.

[64] Kronbichler A, Kresse D, Yoon S (2020). Asymptomatic patients as a source of COVID-19 infections: a systematic review and meta-analysis. Int J Infect Dis.

[65] Africa CDC. COVID-19—Africa CDC. Africa CDC, 2020. https://africacdc.org/COVID-19/.

[66] Mathieu E, Ritchie H, Ortiz-Ospina E (2021). A global database of COVID-19 vaccinations. Nat Hum Behav.

[67] Zhan XY, Zhang Y, Zhou X (2020). Molecular evolution of SARS-CoV-2 structural genes: Evidence of positive selection in spike glycoprotein. bioRxiv..

[68] Volz E, Hill V, McCrone JT (2021). Evaluating the effects of SARS-CoV-2 spike mutation D614G on transmissibility and pathogenicity. Cell.

[69] Harvey WT, Carabelli AM, Jackson B (2021). SARS-CoV-2 variants, spike mutations and immune escape. Nat Rev Microbiol.

[70] Alonso WJ, Viboud C, Simonsen L (2007). Seasonality of influenza in Brazil: a traveling wave from the amazon to the subtropics. Am J Epidemiol.

[71] Martins LD, da Silva I, Batista WV (2020). How socio-economic and atmospheric variables impact COVID-19 and influenza outbreaks in tropical and subtropical regions of Brazil. Environ Res.

[72] Unwin JT, Mishra S, Bradley VC, et al. Report 23: State-level tracking of COVID-19 in the United States. 2020. 10.25561/79231. 10.1038/s41467-020-19652-6 PMC7712910 33273462

[73] Karen Grace-Martin. Assessing the Fit of Regression Models, 2016. https://www.theanalysisfactor.com/Assessing-the-Fit-of-Regression-Models/.

[74] Scarpone C, Brinkmann ST, Große T (2020). A multimethod approach for county-scale geospatial analysis of emerging infectious diseases: a cross-sectional case study of COVID-19 incidence in Germany. Int J Health Geogr.

[75] Kimathi M, Mwalili S, Ojiambo V (2021). Age-structured model for COVID-19: effectiveness of social distancing and contact reduction in Kenya. Infect Dis Model.

[76] Mwalili S, Kimathi M, Ojiambo V (2020). SEIR model for COVID-19 dynamics incorporating the environment and social distancing. BMC Res Notes.

