# Supplementary material for: Using outbreak data to estimate the dynamic COVID-19 landscape in Eastern Africa
Source: BMC Infect Dis. 2022 Jun 9;22:531. doi: 10.1186/s12879-022-07510-3 (PMC9178551; doi:10.1186/s12879-022-07510-3)
Supplement: Supplementary file 1 — Additional file 1. Supplementary figures. Scenario analysis of COVID-19 pandemic using the exponential model (Figure S1 - S6) and the stepwise model (Figure S7 - S12) in Burundi, Ethiopia, Rwanda, South Sudan, Tanzania, and Uganda respectively. [file 12879_2022_7510_MOESM1_ESM.docx]

**Additional file 1: Supporting Figures**

**Using outbreak data to estimate the dynamic COVID-19 landscape in Eastern Africa**

Mark Wamalwa*^1^, Henri E.Z. Tonnang^1^

^1^International Centre of Insect Physiology and Ecology (*icipe*), P.O. Box 30772-00100, Nairobi, Kenya,

*Correspondence to: [mwamalwa@icipe.org](mailto:mwamalwa@icipe.org)


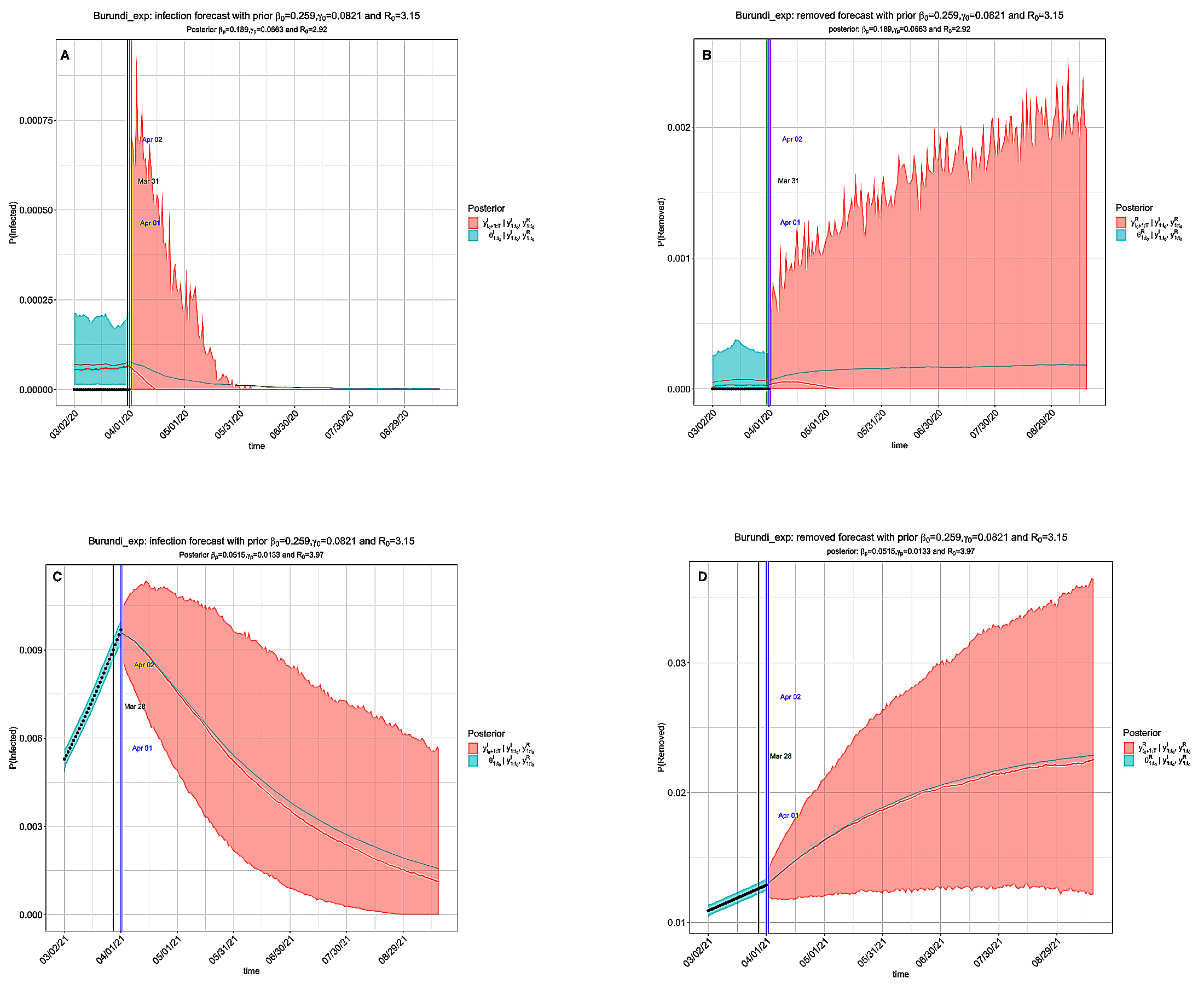


**Figure S1. Estimation of cases-deaths-removed counts using the exponential model under existing interventions in Burundi**. The pandemic peak occurred between March 31 and April 01 2020 (Figure S1 A) and March 28 to April 01 2021 (Figure S1 C). (A, B) The infection and removed (recovered and dead) proportions during 2020/2021 window. The first and second turning points occurred on March 31 and April 01 2020; (C, D) The infection and removed proportions during 2021/2022 window. The first and second turning points occurred on March 28 and April 01 2021. In Figure S1- S12 (Additional file 1): the black dots left of the blue vertical line denote the observed proportions of the infected and removed compartments. The blue vertical line denotes time *t(0)*. The green and purple vertical lines denote the first and second turning points, respectively. The cyan and salmon colour area denotes the 95% credible intervals (CI). The gray and red curves are the posterior mean and median curves [25, 26].


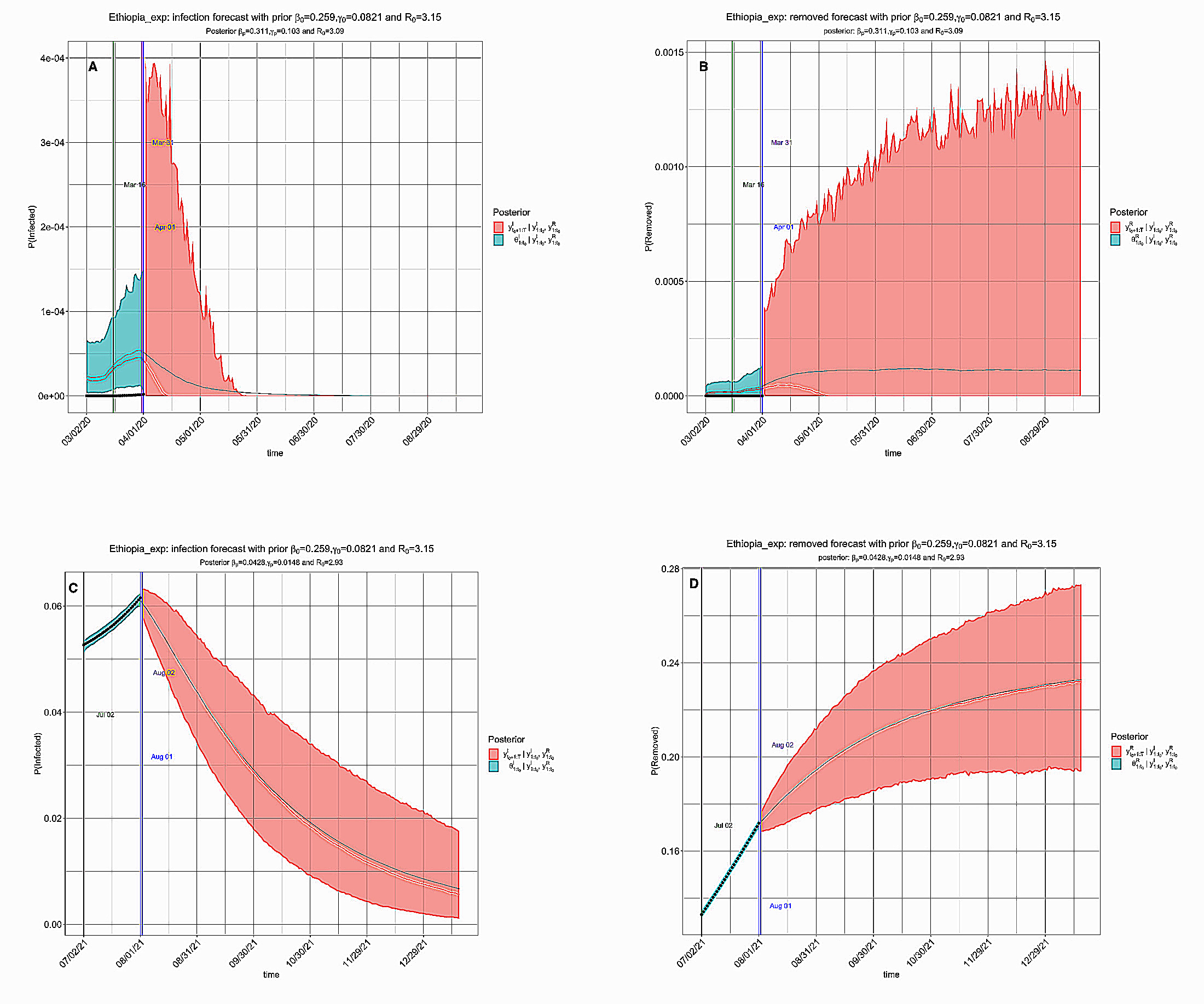


**Figure S2. The exponential model of COVID-19 trends under existing interventions in Ethiopia**. COVID-19 peaked in March 2020 (Figure S2 A) and July 2021 (Figure S2 C). R_0_ decreased from 3.09 in 2020 to 2.93 in 2021. (A, B) Prediction of the infection and removed (recovered and dead) proportions during 2020/2021 window. The first and second turning points occurred on March 16 and March 31 2020; (C, D) Prediction of the infection and removed proportions during 2021/2022 window. The first and second turning points occurred on July 02 and August 02 2021.


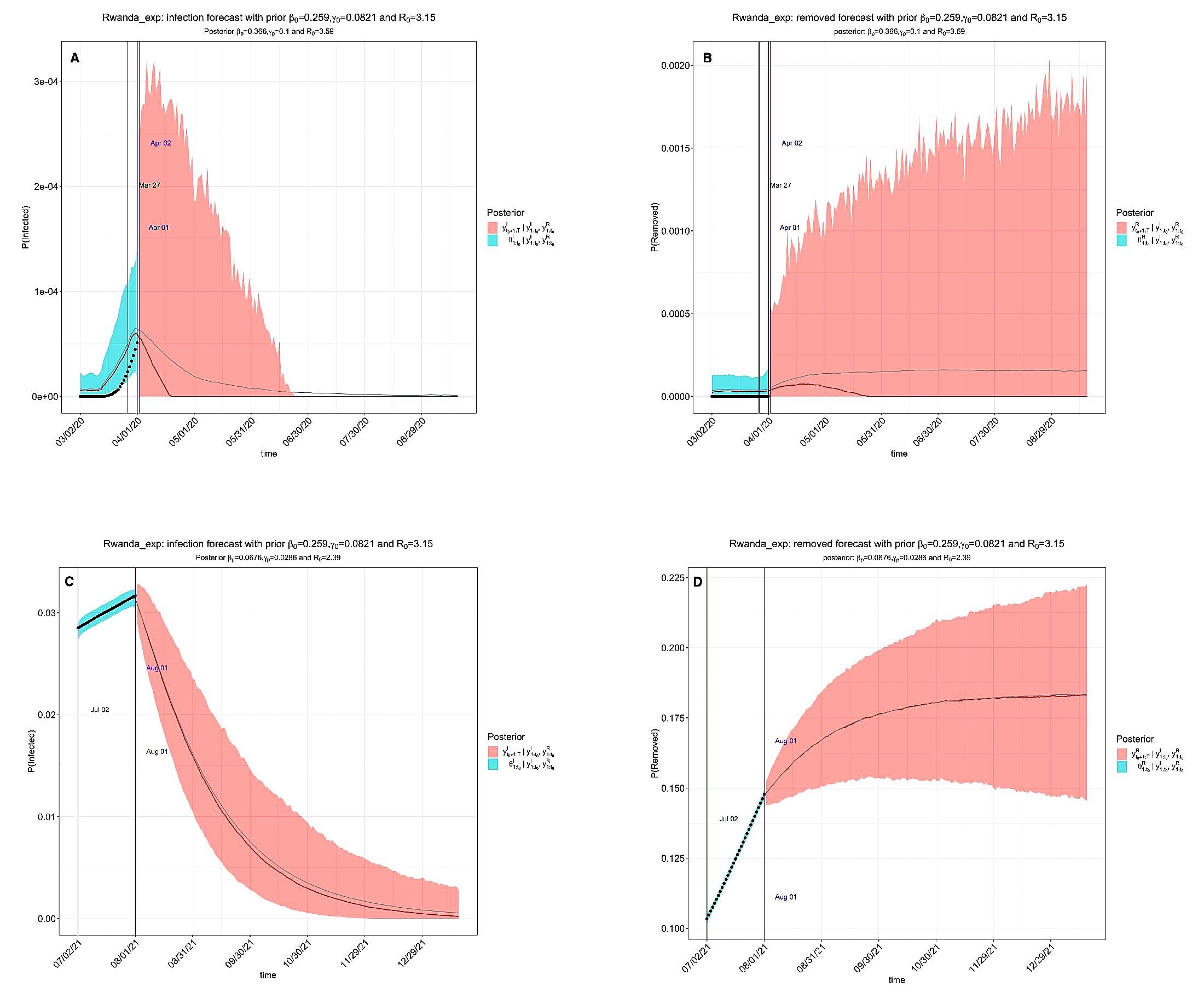


**Figure S3. The exponential model COVID-19 trends under existing interventions in Rwanda**. The peak of COVID-19 occurred in late March 2020 (Figure S3 A) and early August 2021 (Figure S3 C). R_0_ decreased from 3.59 in 2020 to 2.39 in 2021. (A, B) Prediction of the infection and removed (recovered and dead) proportions during 2020/2021 window. The first and second turning points occurred on March 27 and April 01 2020; (C, D) Prediction of the infection and removed proportions during 2021/2022 window. The first and second turning points occurred on July 01 and August 01 2021.


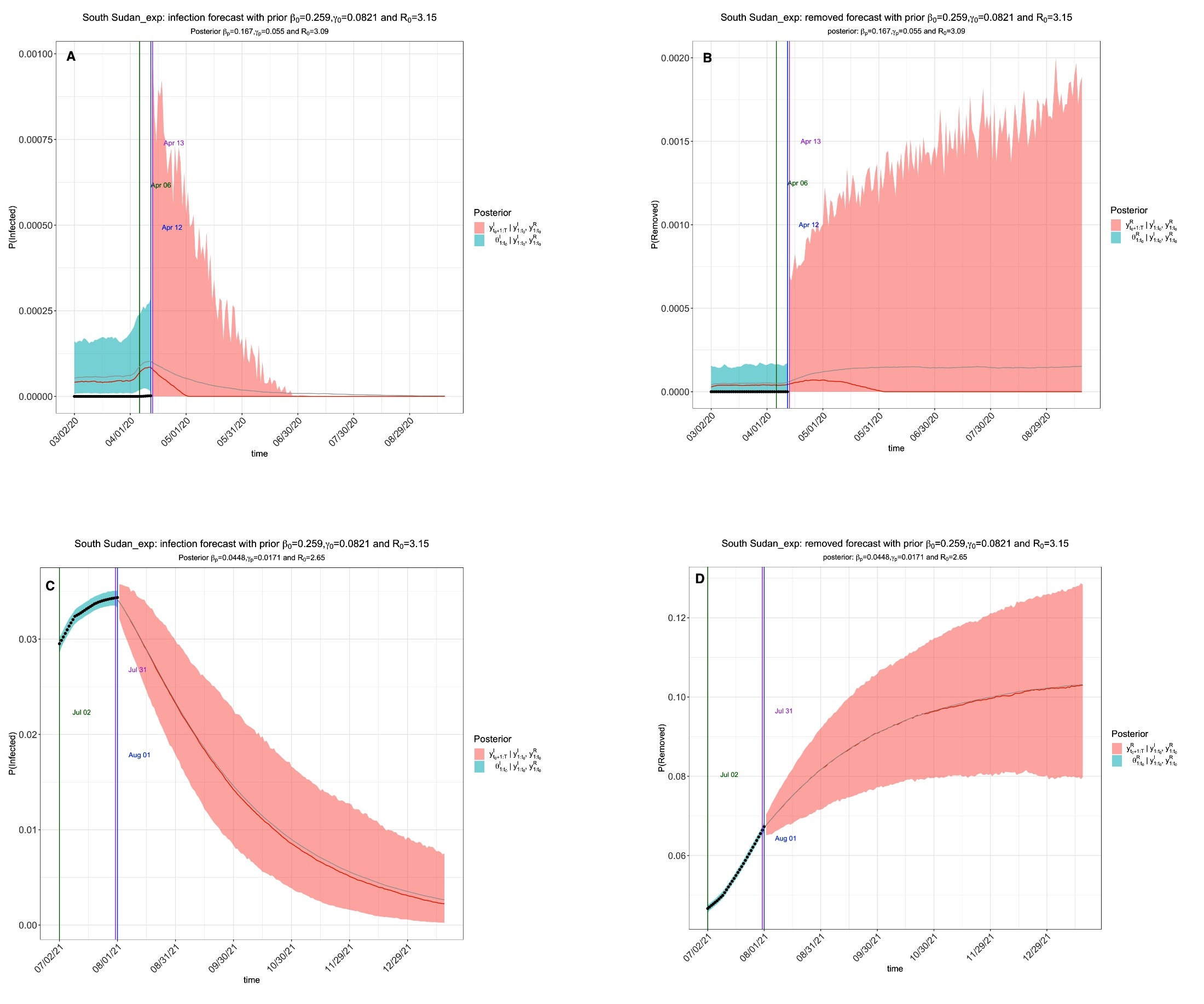
 **Figure S4. The exponential model of COVID-19 trends under existing interventions in South Sudan**. The peak of the pandemic occurred in mid-April 2020 (Figure S4 A) and early August 2021 (Figure S4 C). R_0_ decreased from 3.09 in 2020 to 2.65 in 2021. (A, B) Prediction of the infection and removed (recovered and dead) proportions during 2020/2021 window. The first and second turning points occurred on April 06 and April 12 2020; (C, D) Prediction of the infection and removed proportions during 2021/2022 window. The first and second turning points occurred on July 02 and July 31 2021.


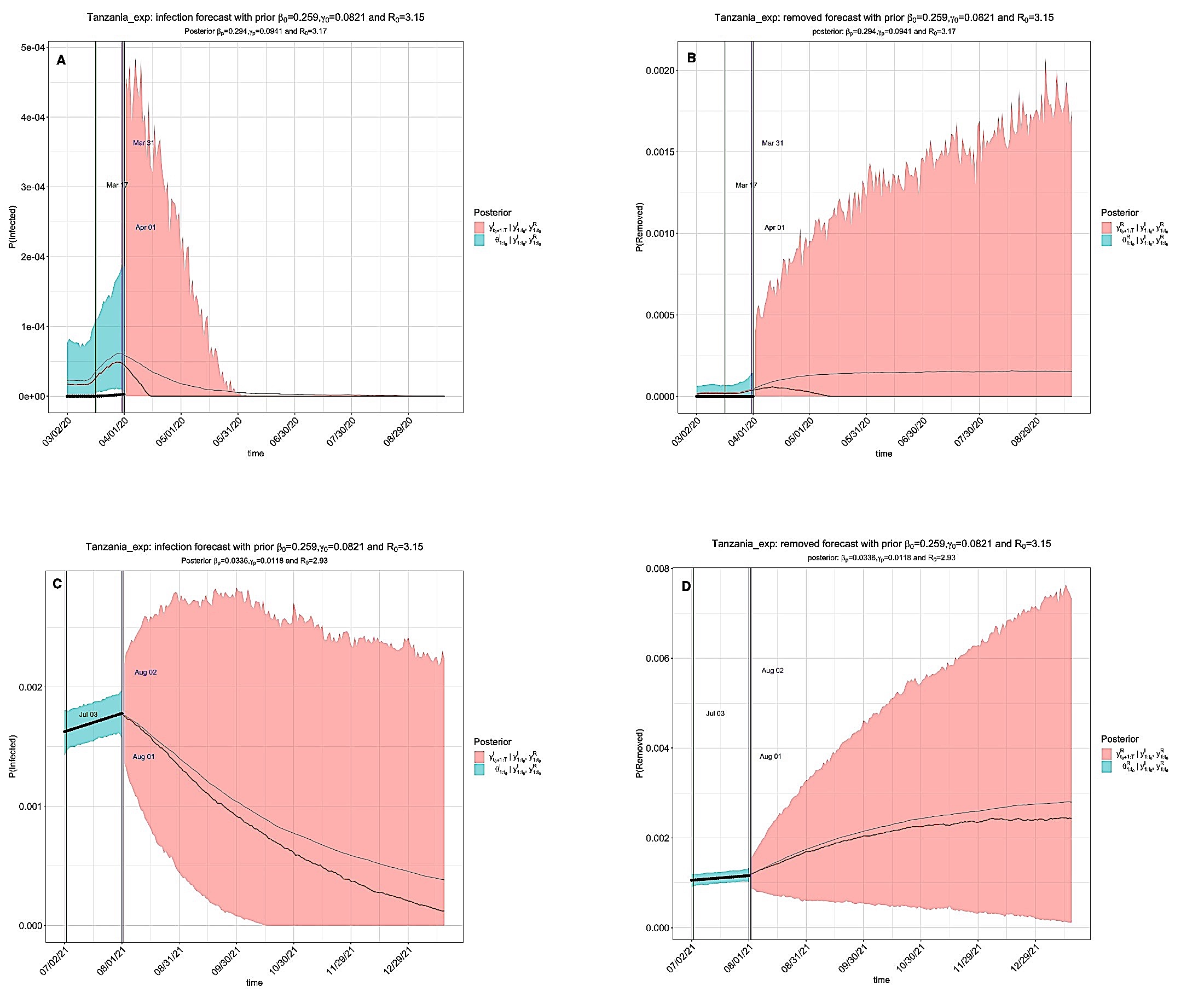
 **Figure S5. The exponential model of COVID-19 trends under existing interventions in Tanzania**. The simulated number of cases peaked in mid-March 2020 (Figure S5 A) and early August 2021 (Figure S5 C). R_0_ decreased from 3.17 in 2020 to 2.93 in 2021. (A, B) Prediction of the infection and removed (recovered and dead) proportions during the 2020/2021 window. The first and second turning points occurred on March 17 and March 31 2020; (C, D) Prediction of the infection and removed proportions of COVID-19 in 2021/2022 window. The first and second turning points occurred on July 03 and August 01 2021.


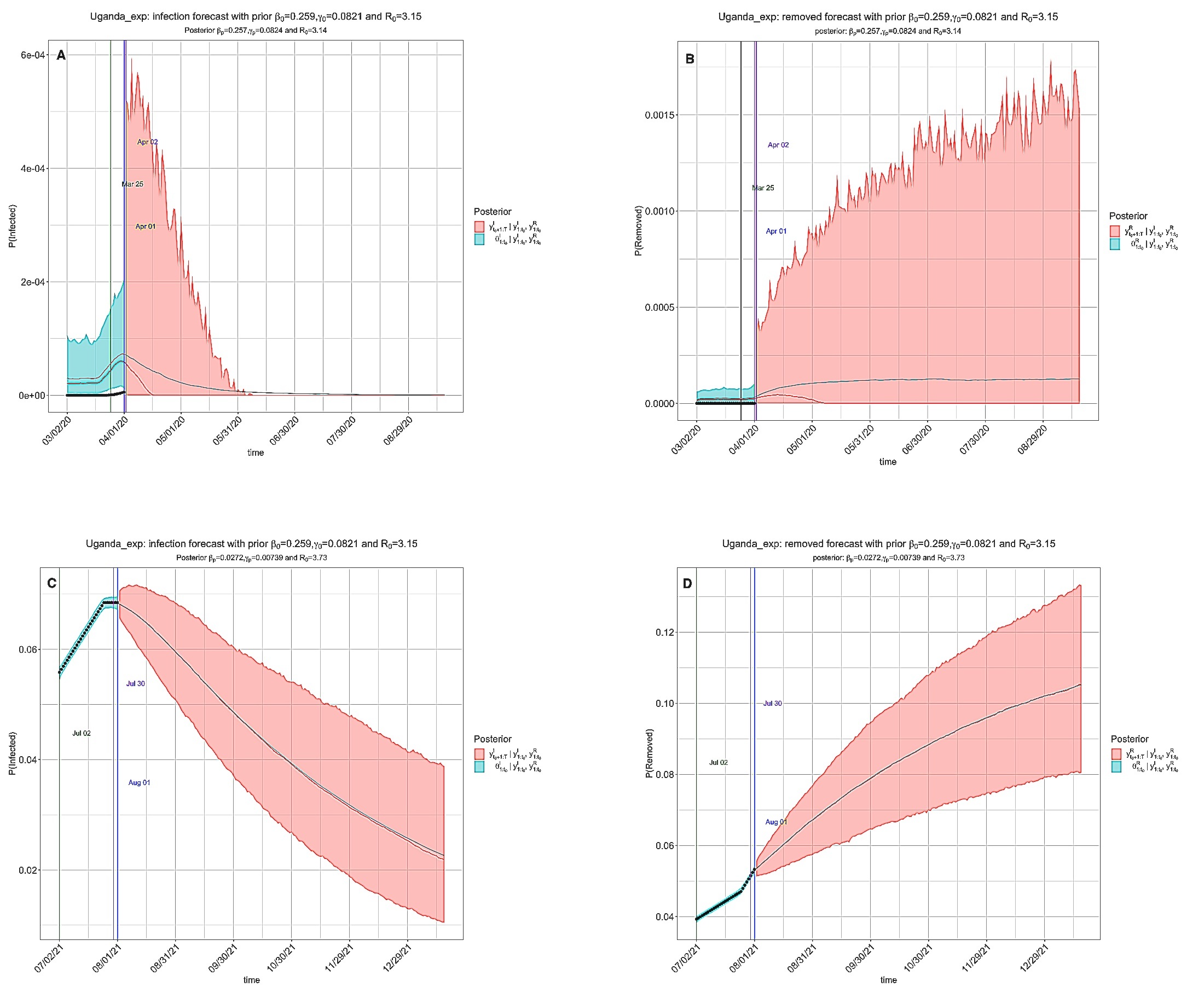
 **Figure S6. The exponential model of COVID-19 trends under existing interventions in Uganda**. The simulated number of cases peaked in late-March 2020 (Figure S6 A) and early August 2021 (Figure S6 C). R_0_ increased from 3.14 in 2020 to 3.73 in 2021. (A, B) Prediction of the infection and removed (recovered and dead) proportions in 2020/2021 window. The first and second turning points occurred on March 25 and April 01 2020; (C, D) Prediction of the infection and removed proportions in 2021/2022 window. The first and second turning points occurred on July 02 and July 30 2021.


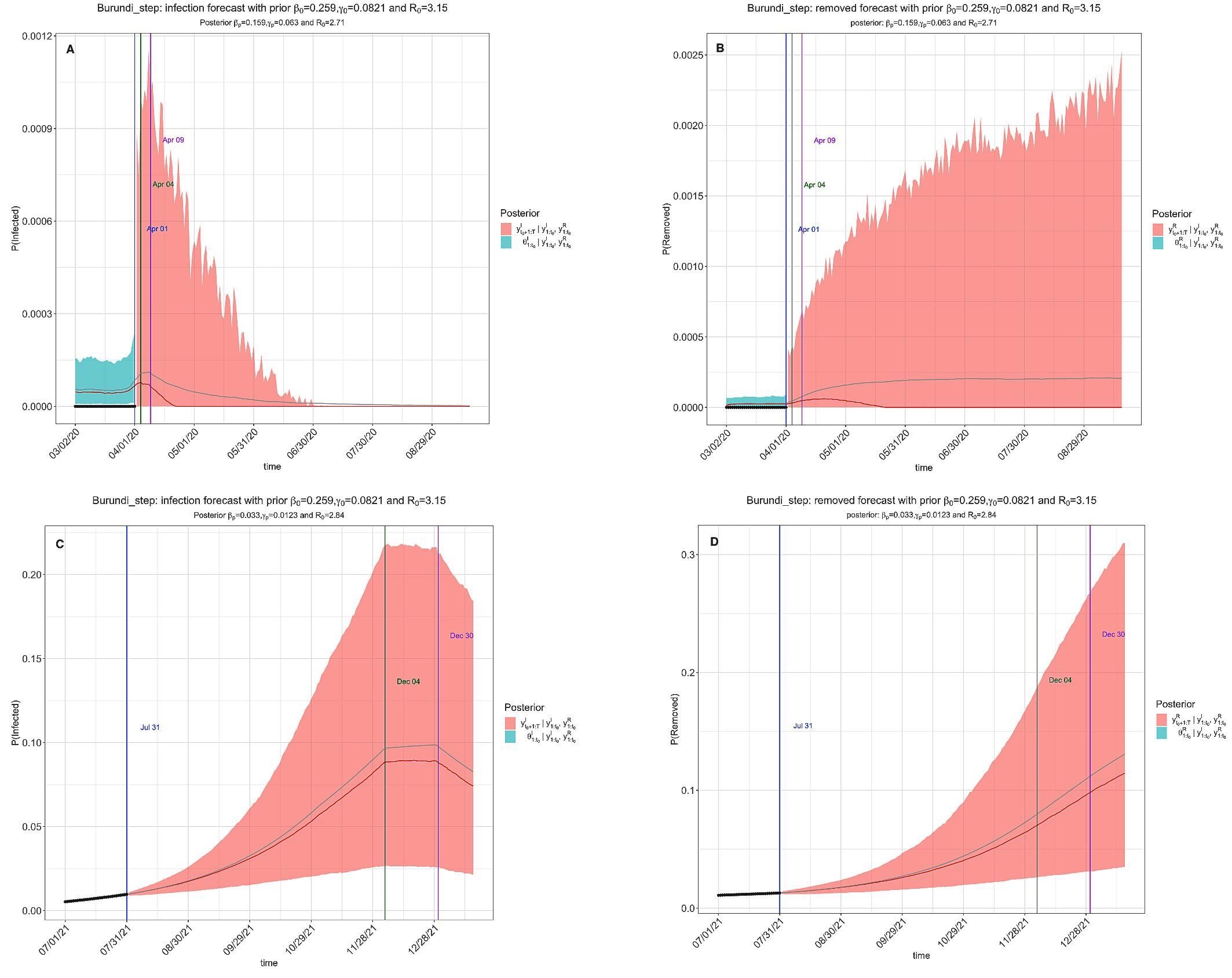
 **Figure S7. The stepwise model of COVID-19 trends under existing interventions in Burundi**. The simulated number of cases peaked in early April 2020 (Figure S7 A) and early August 2021 (Figure S7 C). R_0_ increased from 2.71 in 2020 to 2.84 in 2021. (A, B) Prediction of the infection and removed (recovered and dead) proportions during 2020/2021 window. The first and second turning points occurred on April 01 and April 04 2020; (C, D) Prediction of the infection and removed proportions during 2021/2022 window. The first and second turning points occurred on July 31 and December 04 2021.


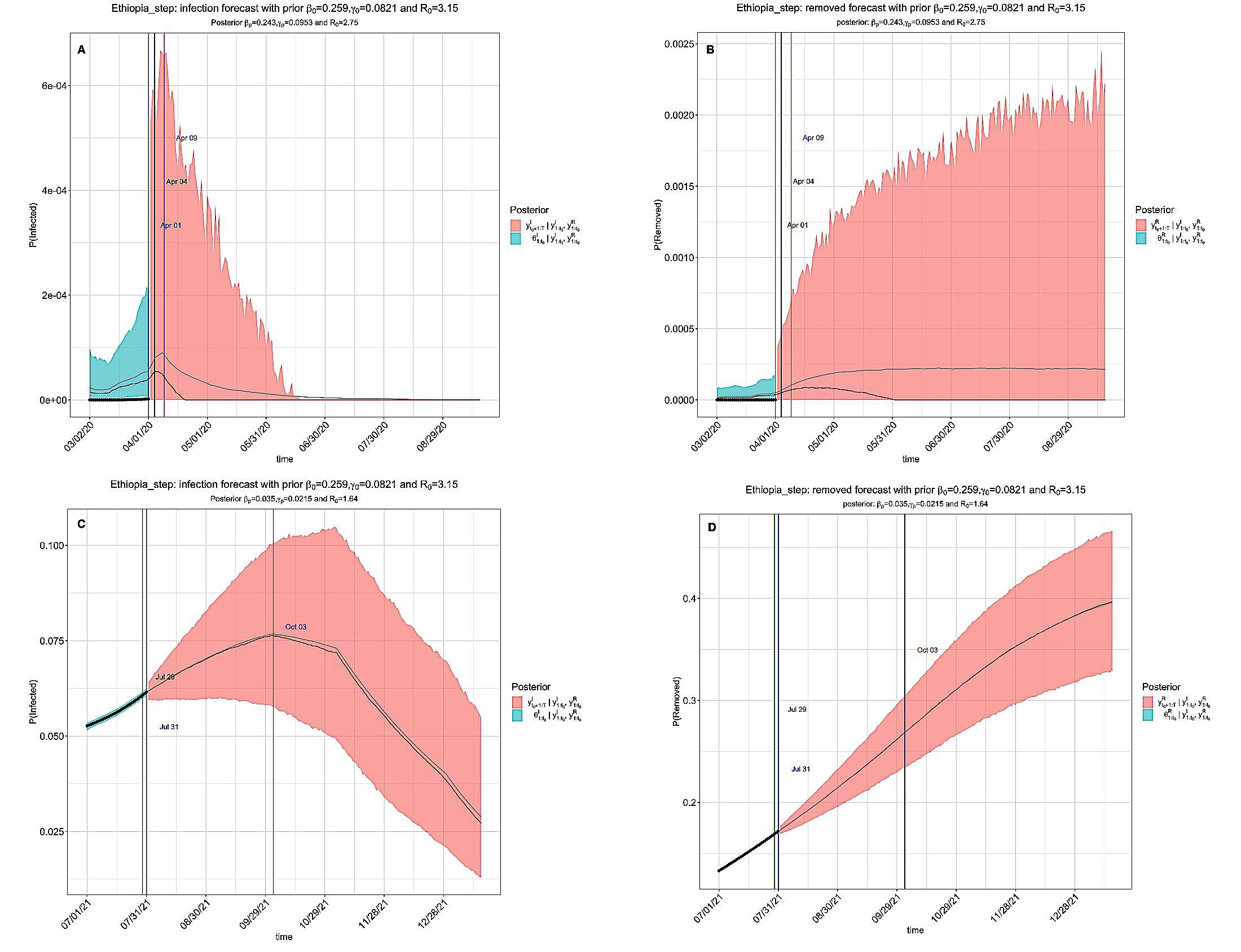
 **Figure S8. The stepwise model of COVID-19 trends under existing interventions in Ethiopia**. The simulated pandemic peak occurred in early April 2020 (Figure S8 A) and late August 2021 (Figure S8 C). R_0_ decreased from 2.75 in 2020 to 1.64 in 2021. (A, B) Prediction of the infection and removed (recovered and dead) proportions during 2020/2021 window. The first and second turning points occurred on April 01 and April 04 2020; (C, D) Prediction of the infection and removed proportions during 2021/2022 window. The first and second turning points occurred on July 29 and July 31 2021.


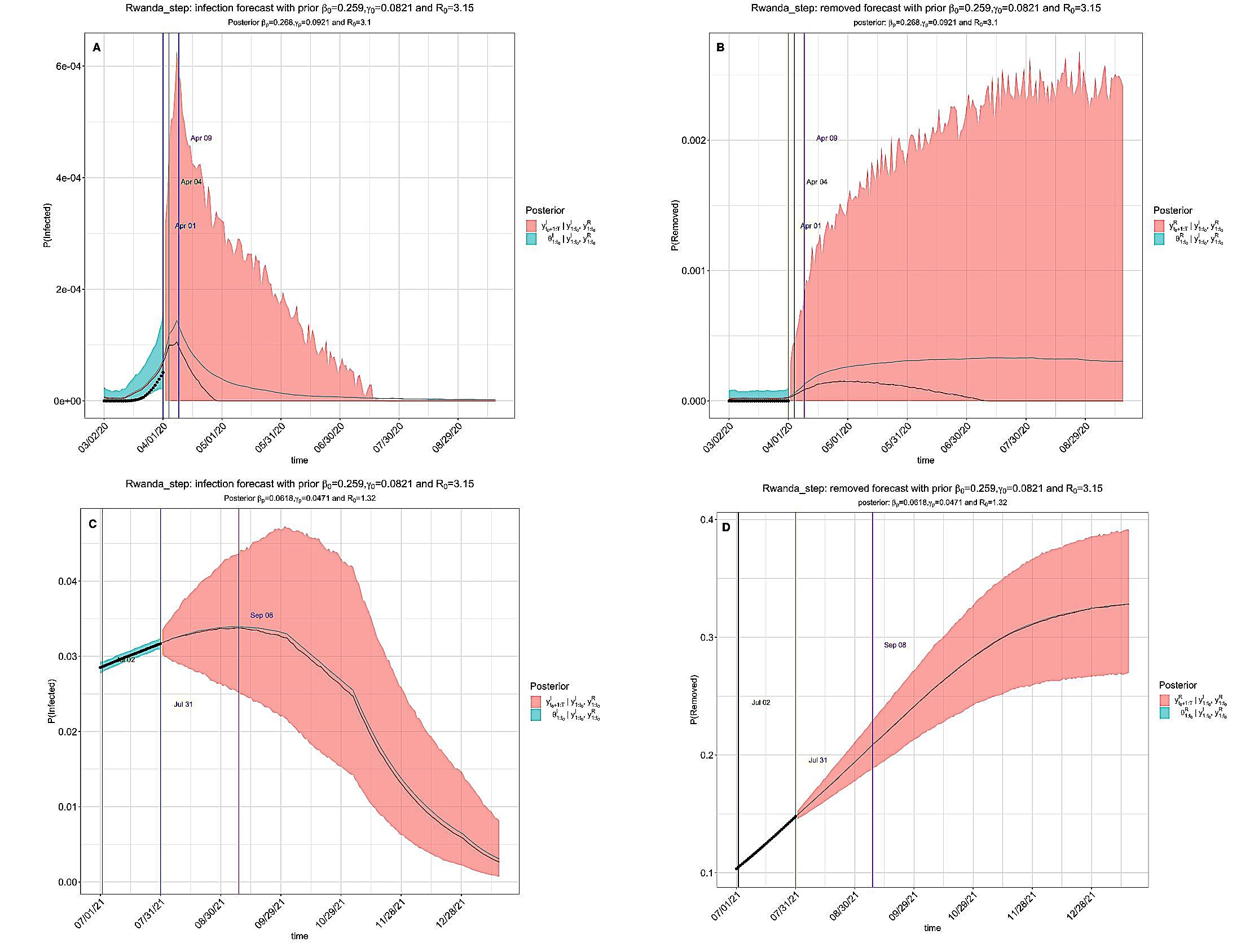
 **Figure S9. The stepwise model of COVID-19 trends under existing interventions in Rwanda**. The simulated pandemic peak occurred in early April 2020 (Figure S9 A) and July 2021 (Figure S9 C). R_0_ decreased from 3.10 in 2020 to 1.32 in 2021. (A, B) Prediction of the infection and removed (recovered and dead) proportions during 2020/2021 window. The first and second turning points occurred on April 01 and April 04 2020; (C, D) Prediction of the infection and removed proportions during 2021/2022 window. The first and second turning points occurred on July 02 and July 31 2021.


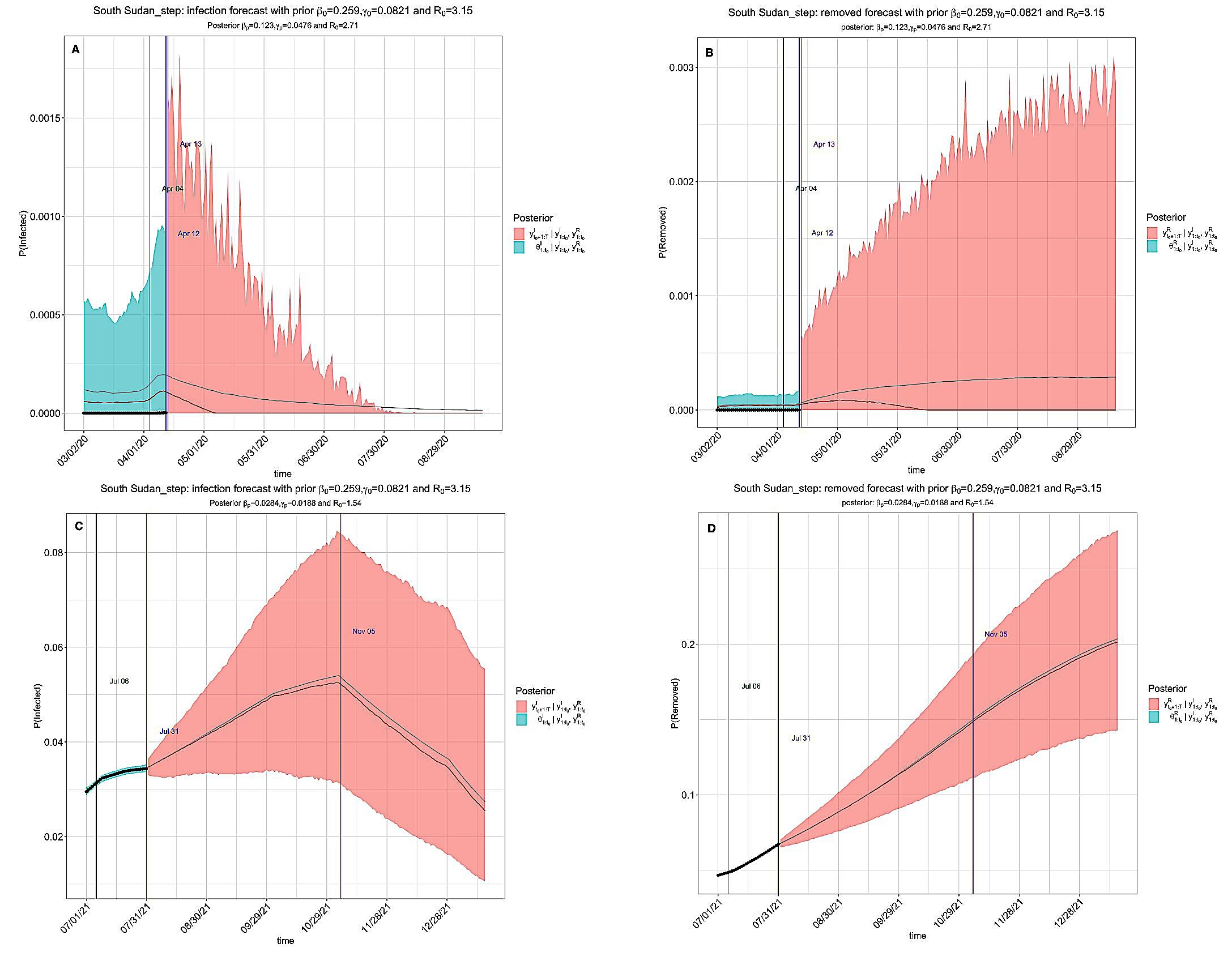
 **Figure S10. The stepwise model of COVID-19 trends under existing interventions in South Sudan**. The simulated number of cases peaked in mid-April 2020 (Figure S10 A) and early August 2021 (Figure S10 C). R_0_ decreased from 2.71 in 2020 to 1.54 in 2021. (A, B) Prediction of the infection and removed (recovered and dead) proportions during 2020/2021 window. The first and second turning points occurred on April 04 and April 12 2020; (C, D) Prediction of the infection and removed proportions during 2021/2022 window. The first and second turning points occurred on July 06 and July 31 2021.


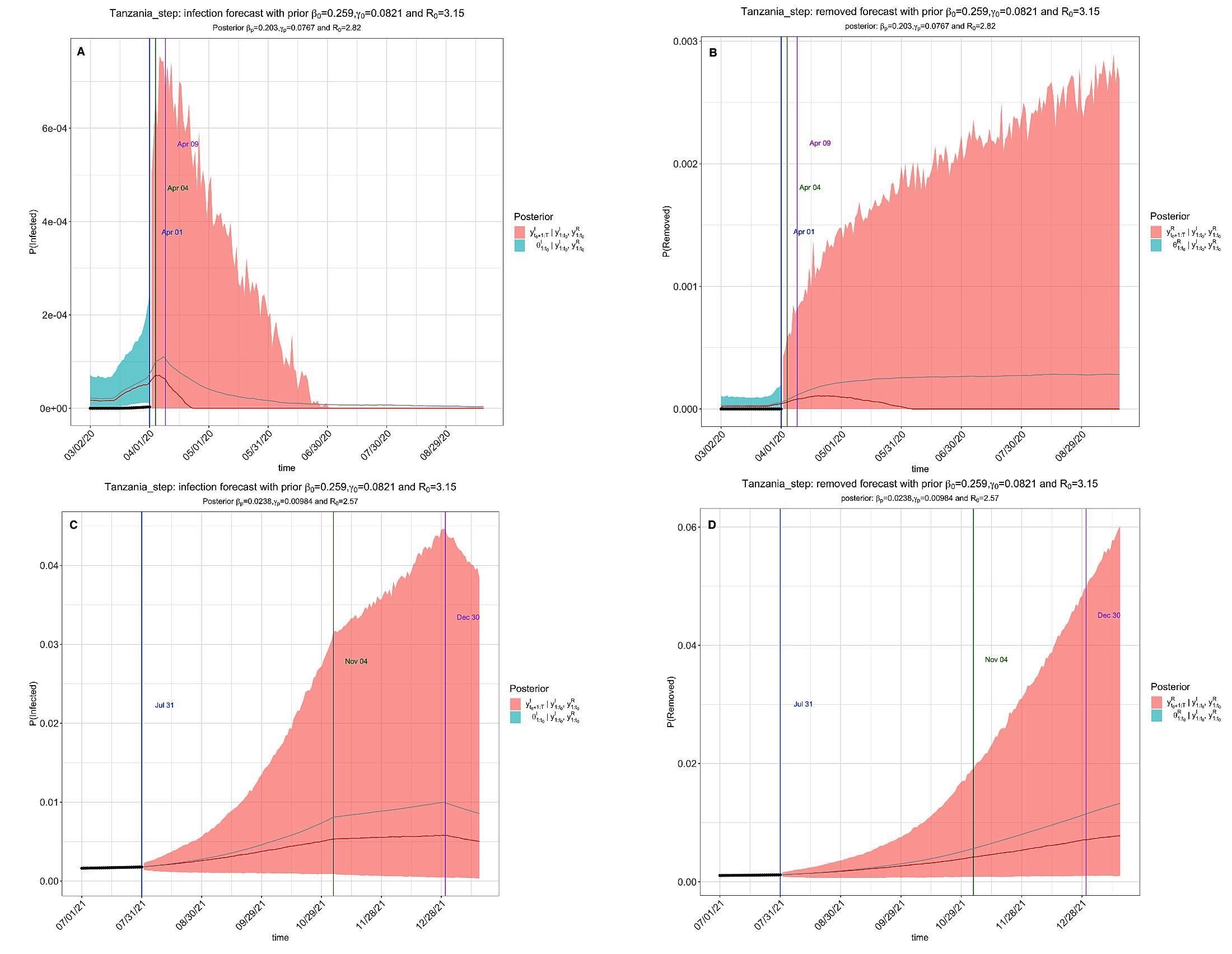
 **Figure S11. The stepwise model of COVID-19 trends under existing interventions in Tanzania**. The simulated number of cases peaked in early April 2020 (Figure S11 A) and August 2021 (Figure S11 C). There was a slight decrease in R_0_ from 2.82 in 2020 to 2.57 in 2021. (A, B) Prediction of the infection and removed (recovered and dead) proportions during 2020/2021 window. The first and second turning points occurred on April 01 and April 04 2020; (C, D) Prediction of the infection and removed proportions during 2021/2022 window. The first and second turning points occurred on July 31 and November 04 2021.


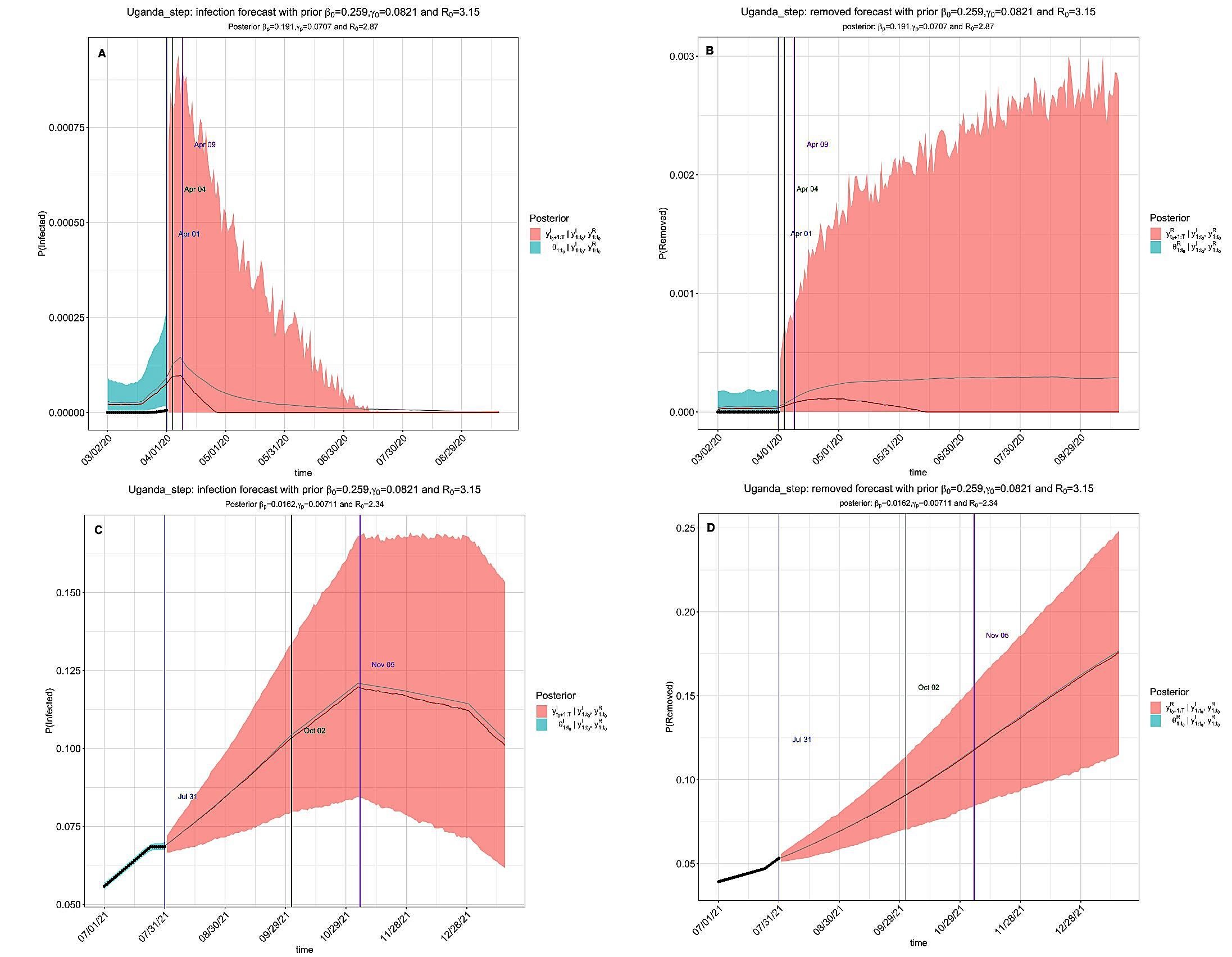
 **Figure S12. The stepwise model of COVID-19 trends under existing interventions in Uganda**. The simulated pandemic peak occurred in early April 2020 (Figure S12 A) and October 2021 (Figure S12 C). There was a slight decrease in R_0_ from 2.87 in 2020 to 2.34 in 2021. (A, B) Prediction of the infection and removed (recovered and dead) proportions during 2020/2021 window. The first and second turning points occurred on April 01 and April 04 2020; (C, D) Prediction of the infection and removed proportions during 2021/2022 window. The first and second turning points occurred on July 31 and October 02 2021.
