# Supplementary material for: Using outbreak data to estimate the dynamic COVID-19 landscape in Eastern Africa
Source: BMC Infect Dis. 2022 Jun 9;22:531. doi: 10.1186/s12879-022-07510-3 (PMC9178551; doi:10.1186/s12879-022-07510-3)
Supplement: Supplementary file 2 — Additional file 2. Supplementary figures. Projections of COVID-19 epidemic trends in Burundi, Ethiopia, Rwanda, South Sudan, Tanzania, and Uganda using the standard state-space SIR model without interventions (Figure S13 – S18). [file 12879_2022_7510_MOESM2_ESM.docx]

**Additional file 2: Supporting Figures**

**Using outbreak data to estimate the dynamic COVID-19 landscape in Eastern Africa**

Mark Wamalwa*^1^, Henri E.Z. Tonnang^1^

^1^International Centre of Insect Physiology and Ecology (*icipe*), P.O. Box 30772-00100, Nairobi, Kenya,

*Correspondence to: [mwamalwa@icipe.org](mailto:mwamalwa@icipe.org)


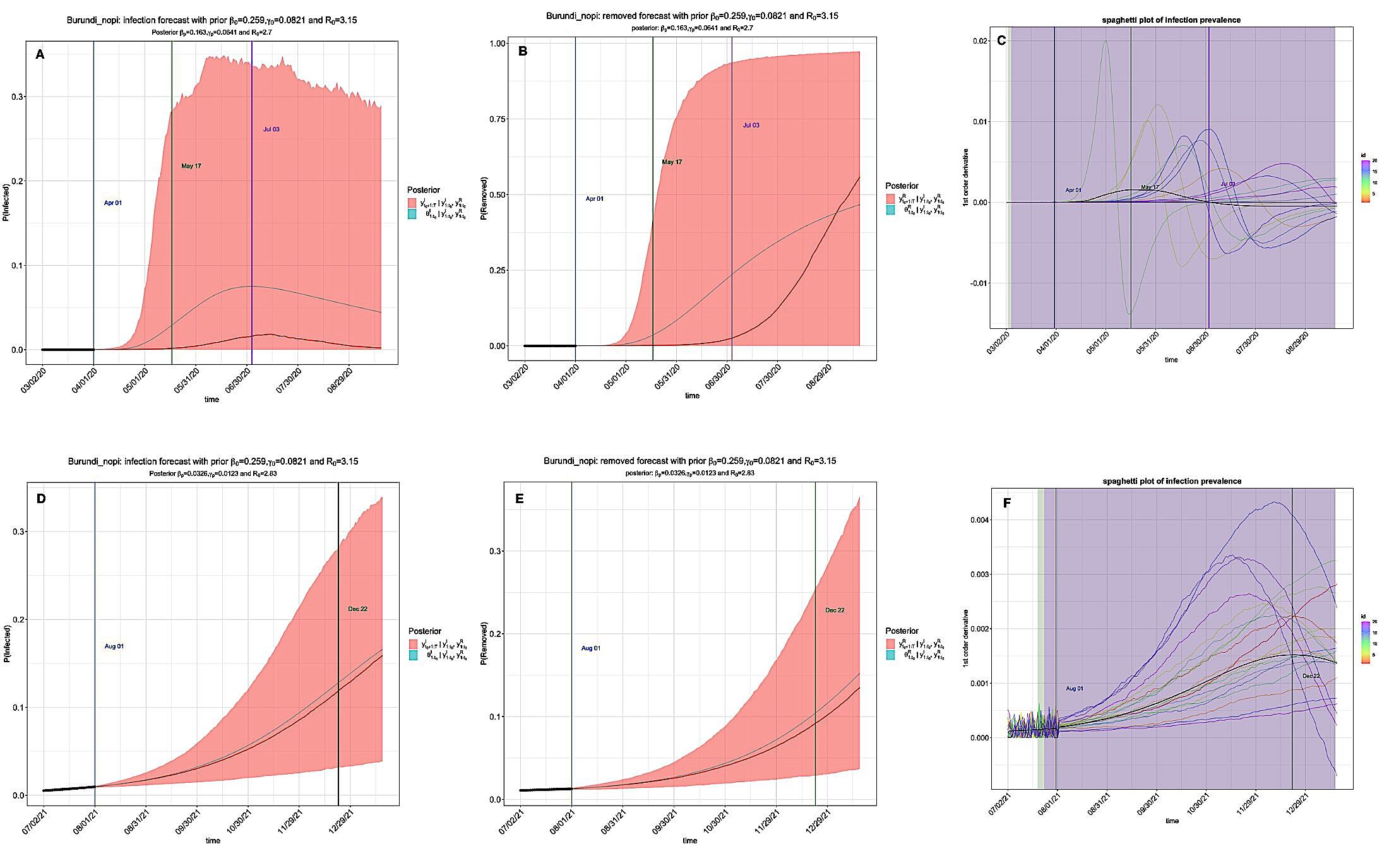
 **Figure S13. Standard state-space SIR model of COVID-19 trend in Burundi without interventions**. The simulated pandemic peak occurred in April to May 2020 (Figure S13 A) and August-December 2021 (Figure S13 D). There was an increase in R_0_ from 2.70 in 2020 to 2.83 in 2021. (A, B) Prediction of the infection and removed (recovered and dead) proportions during 2020/2021 window. The first and second turning points occurred on April 01 and May 17 2020; (C) Plot of the first-order derivatives of the posterior prevalence of infection in 2020/2021. (D, E) Prediction of the infection and removed proportions during 2021/2022 window. The first and second turning points occurred on August 01 and December 22 2021; (F) Plot of the first-order derivatives of the posterior prevalence of infection for 2021/2022 time period. In Figure S13 – S24, plots of the first-order derivatives: the colored semi-transparent rectangles represent the 95% CI of these turning points. The black curve is the posterior mean of the derivative, and the vertical lines mark times of turning points corresponding respectively to those shown (A, B) and (D, E).


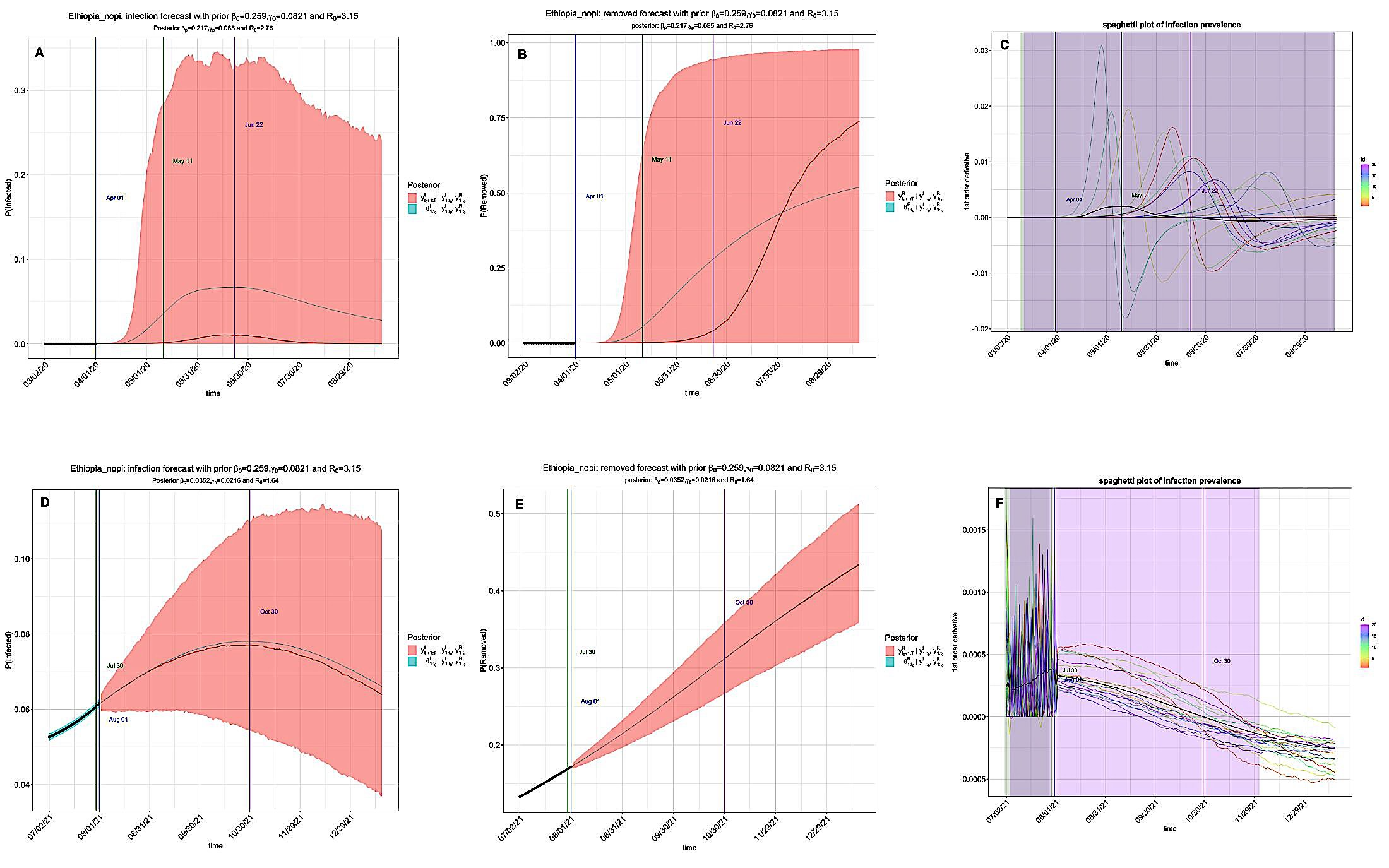
 **Figure S14. Standard state-space SIR model of COVID-19 trend in Ethiopia without interventions**. The simulated pandemic peak occurred in April to May 2020 (Figure S14 A) and August to September 2021 (Figure S14 D). There was a decrease in R_0_ from 2.76 in 2020 to 1.64 in 2021. (A, B) Prediction of the infection and removed (recovered and dead) proportions during 2020/2021 window. The first and second turning points occurred on April 01 and May 11 2020; (C) Plot of the first-order derivatives of the posterior prevalence of infection in 2020/2021. (D, E) Prediction of the infection and removed proportions during 2021/2022 window. The first and second turning points occurred on July 30 and August 01 2021; (F) Plot of the first-order derivatives of the posterior prevalence of infection for 2021/2022 time period.


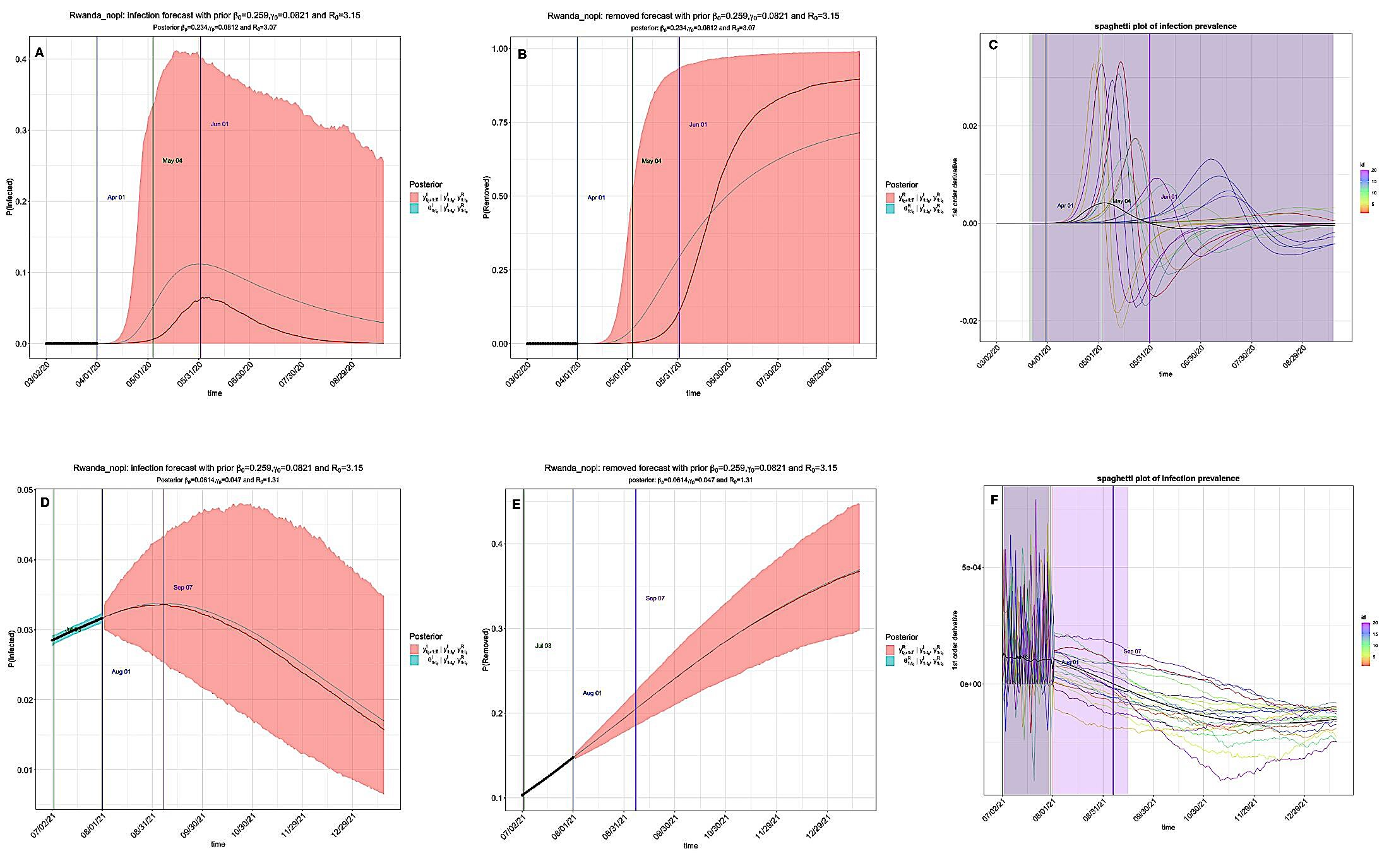
 **Figure S15. COVID-19 trend in Rwanda using the standard state-space SIR model without interventions**. The simulated pandemic peak occurred in May 2020 (Figure S15 A) and August 2021 (Figure S15 D). There was a decrease in R_0_ from 3.07 in 2020 to 1.31 in 2021. (A, B) Prediction of the infection and removed (recovered and dead) proportions during 2020/2021 window. The first and second turning points occurred on April 01 and May 04 2020; (C) Plot of the first-order derivatives of the posterior prevalence of infection in 2020/2021. (D, E) Prediction of the infection and removed proportions during 2021/2022 window. The first and second turning points occurred on July 03 and August 01 2021; (F) Plot of the first-order derivatives of the posterior prevalence of infection for 2021/2022 time period.


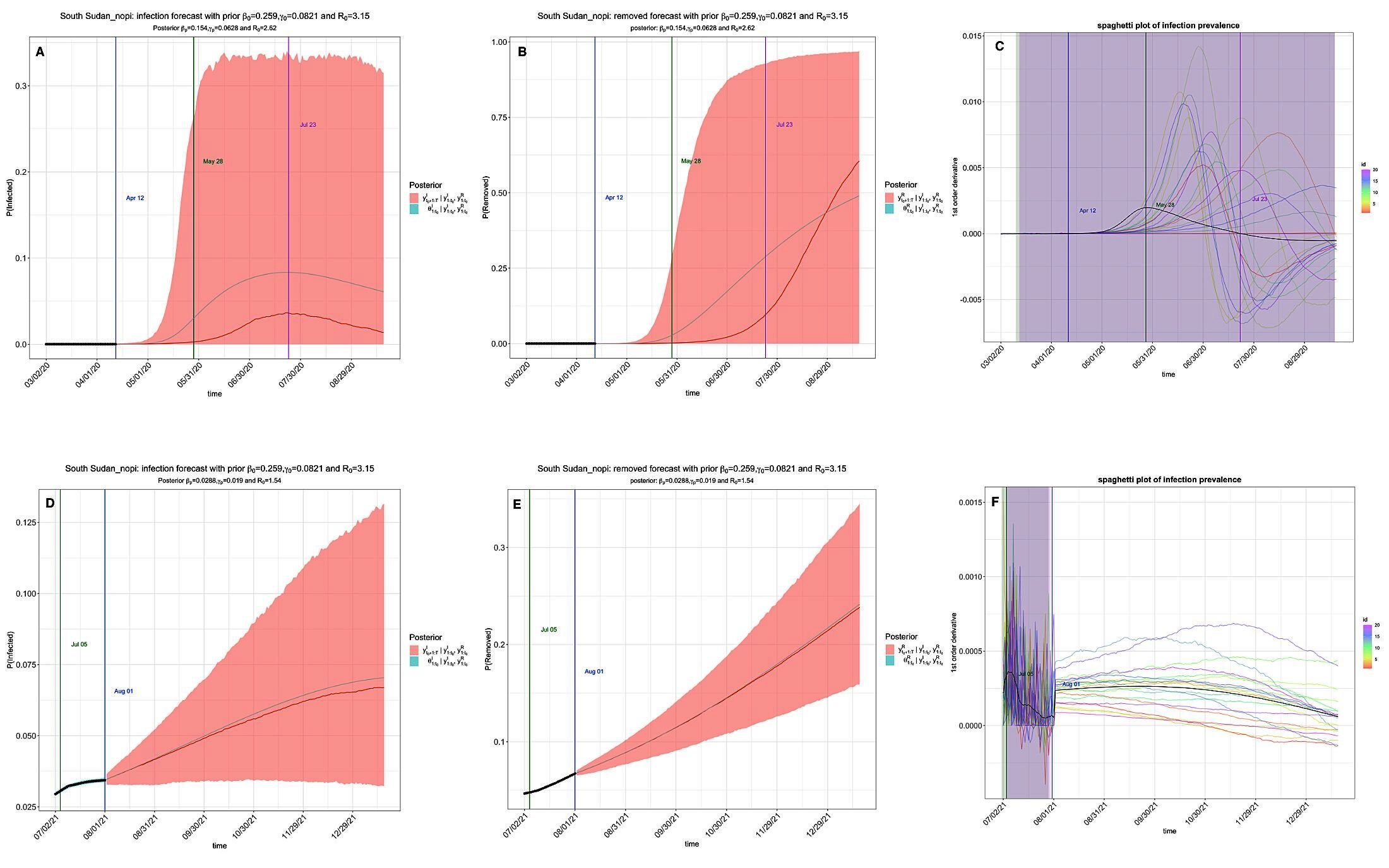
 **Figure S16. COVID-19 trend in South Sudan using the standard SIR model without interventions**. The simulated number of cases peaked in April to May 2020 (Figure S16 A) and August 2021 (Figure S16 D). R_0_ decreased from 2.62 in 2020 to 1.54 in 2021. (A, B) Prediction of the infection and removed (recovered and dead) proportions during 2020/2021 window. The first and second turning points occurred on April 12 and May 28 2020; (C) Plot of the first-order derivatives of the posterior prevalence of infection in 2020/2021. (D, E) Prediction of the infection and removed proportions during 2021/2022 window. The first and second turning points occurred on July 05 and August 01 2021; (F) Plot of the first-order derivatives of the posterior prevalence of infection for 2021/2022 time period.


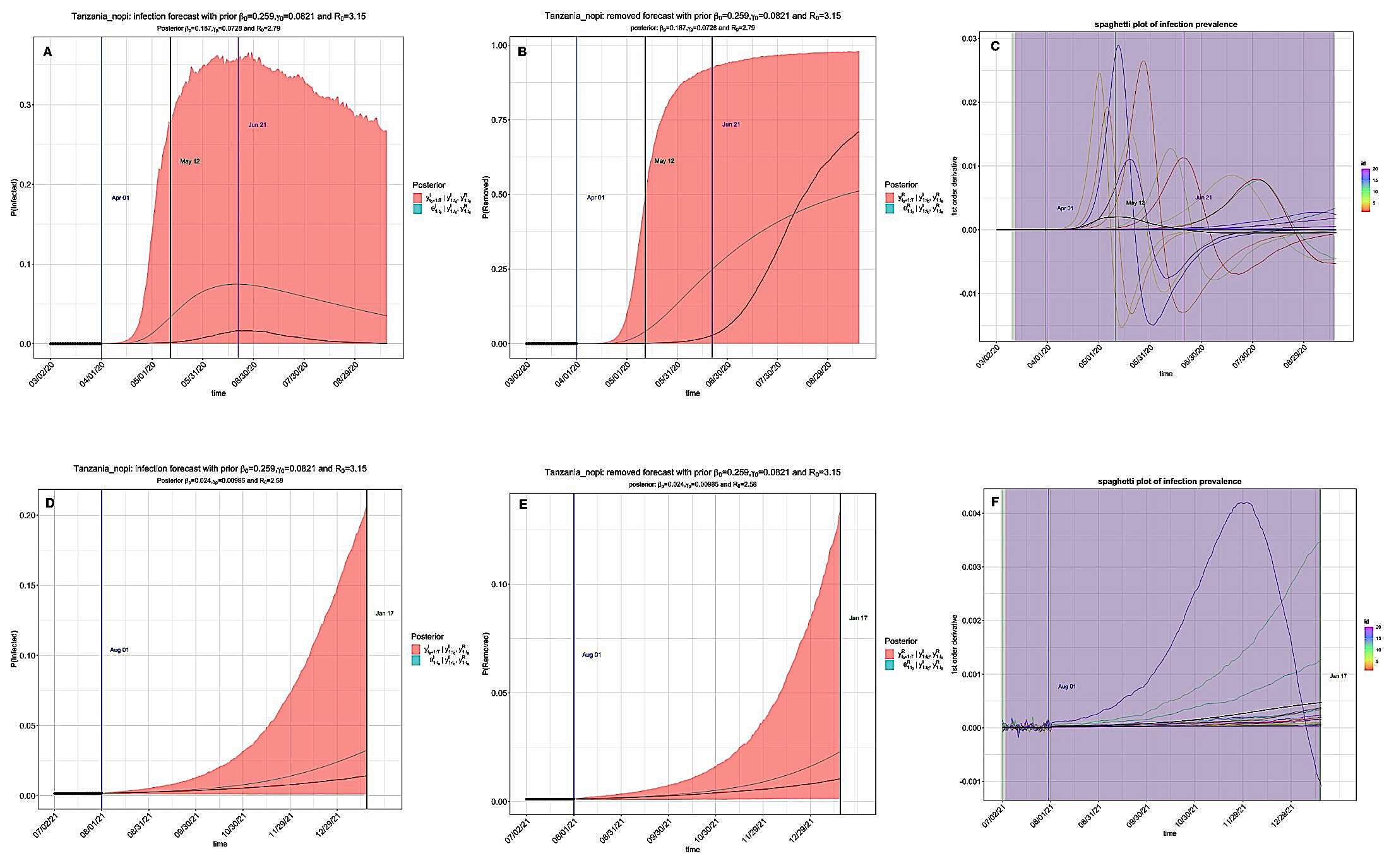
 **Figure S17. Estimation of COVID-19 trend in Tanzania using the standard SIR model without interventions**. The number of cases peaked in April to May 2020 (Figure S17 A) and August to December 2021 (Figure S17 D). R_0_ decreased from 2.79 in 2020 to 2.58 in 2021. (A, B) Prediction of the infection and removed (recovered and dead) proportions during 2020/2021 window. The first and second turning points occurred on April 01 and May 12 2020; (C) Plot of the first-order derivatives of the posterior prevalence of infection in 2020/2021. (D, E) Prediction of the infection and removed proportions during 2021/2022 window. The first and second turning points occurred on August 01 and January 17 2021; (F) Plot of the first-order derivatives of the posterior prevalence of infection for 2021/2022 time period.


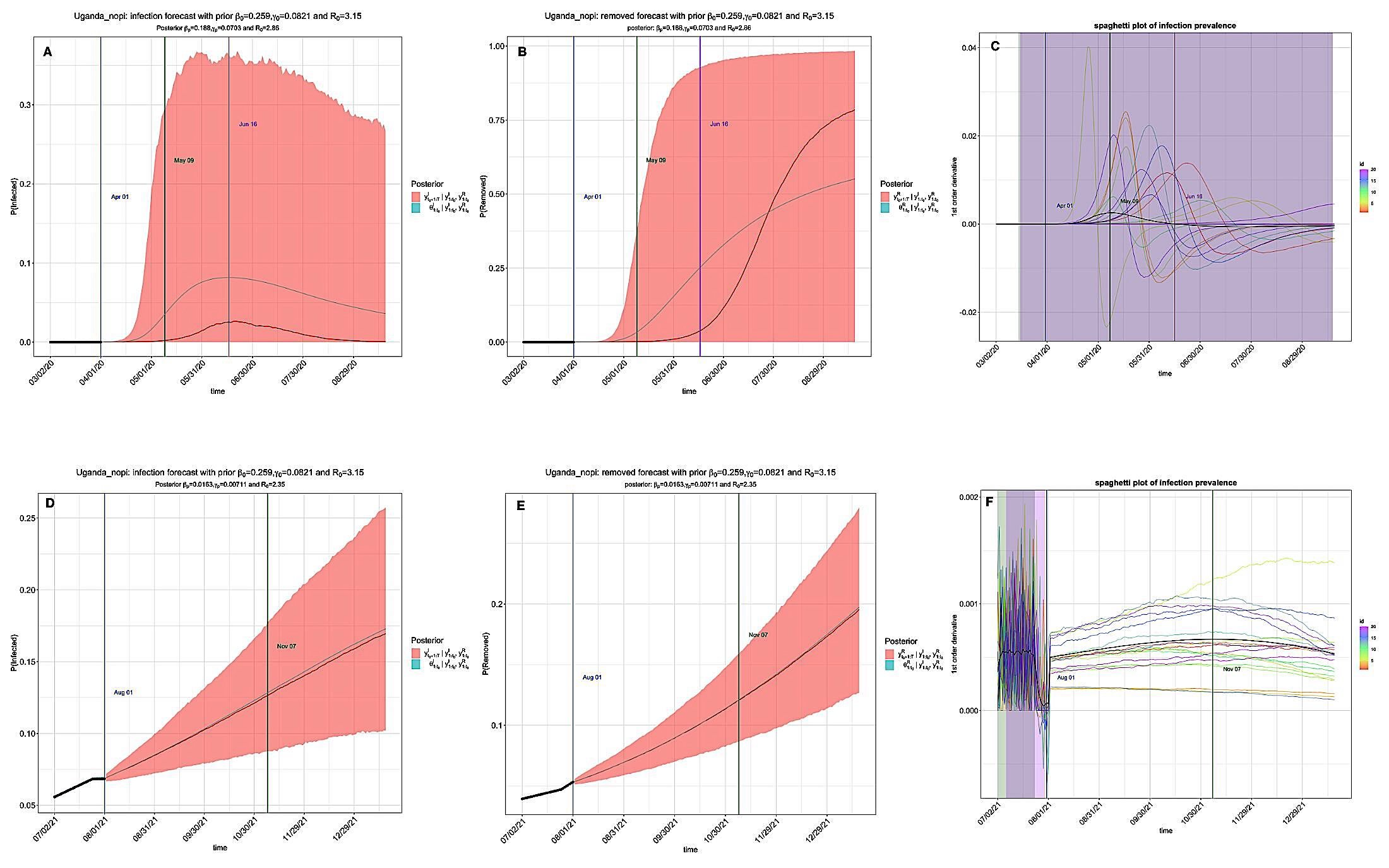
 **Figure S18. COVID-19 trend in Uganda using the standard state-space SIR model without interventions**. The simulated number of cases peaked in May 2020 (Figure S18 A) and August to November 2021 (Figure S18 D). R_0_ decreased from 2.86 in 2020 to 2.35 in 2021. (A, B) Prediction of the infection and removed (recovered and dead) proportions during 2020/2021 window. The first and second turning points occurred on April 01 and May 09 2020; (C) Plot of the first-order derivatives of the posterior prevalence of infection in 2020/2021. (D, E) Prediction of the infection and removed proportions during 2021/2022 window. The first and second turning points occurred on August 01 and November 07 2021; (F) Plot of the first-order derivatives of the posterior prevalence of infection for 2021/2022 time period.
