# Supplementary material for: Using outbreak data to estimate the dynamic COVID-19 landscape in Eastern Africa
Source: BMC Infect Dis. 2022 Jun 9;22:531. doi: 10.1186/s12879-022-07510-3 (PMC9178551; doi:10.1186/s12879-022-07510-3)
Supplement: Supplementary file 3 — Additional file 3. Supplementary figures. Estimation of COVID-19 epidemic trends using a time-varying quarantine model (Figure S19 - S24) and scenario projection of herd immunity and vaccination campaign in Burundi, Ethiopia, Rwanda, South Sudan, Tanzania, and Uganda respectively (Figure S25 - S30). Further estimates of R0 values across using the multinomial-2-parameter SEIR model (Figure S31). [file 12879_2022_7510_MOESM3_ESM.docx]

**Additional file 3: Supporting Figures**

**Using outbreak data to estimate the dynamic COVID-19 landscape in Eastern Africa**

Mark Wamalwa*^1^, Henri E.Z. Tonnang^1^

^1^International Centre of Insect Physiology and Ecology (*icipe*), P.O. Box 30772-00100, Nairobi, Kenya,

*Correspondence to: [mwamalwa@icipe.org](mailto:mwamalwa@icipe.org)


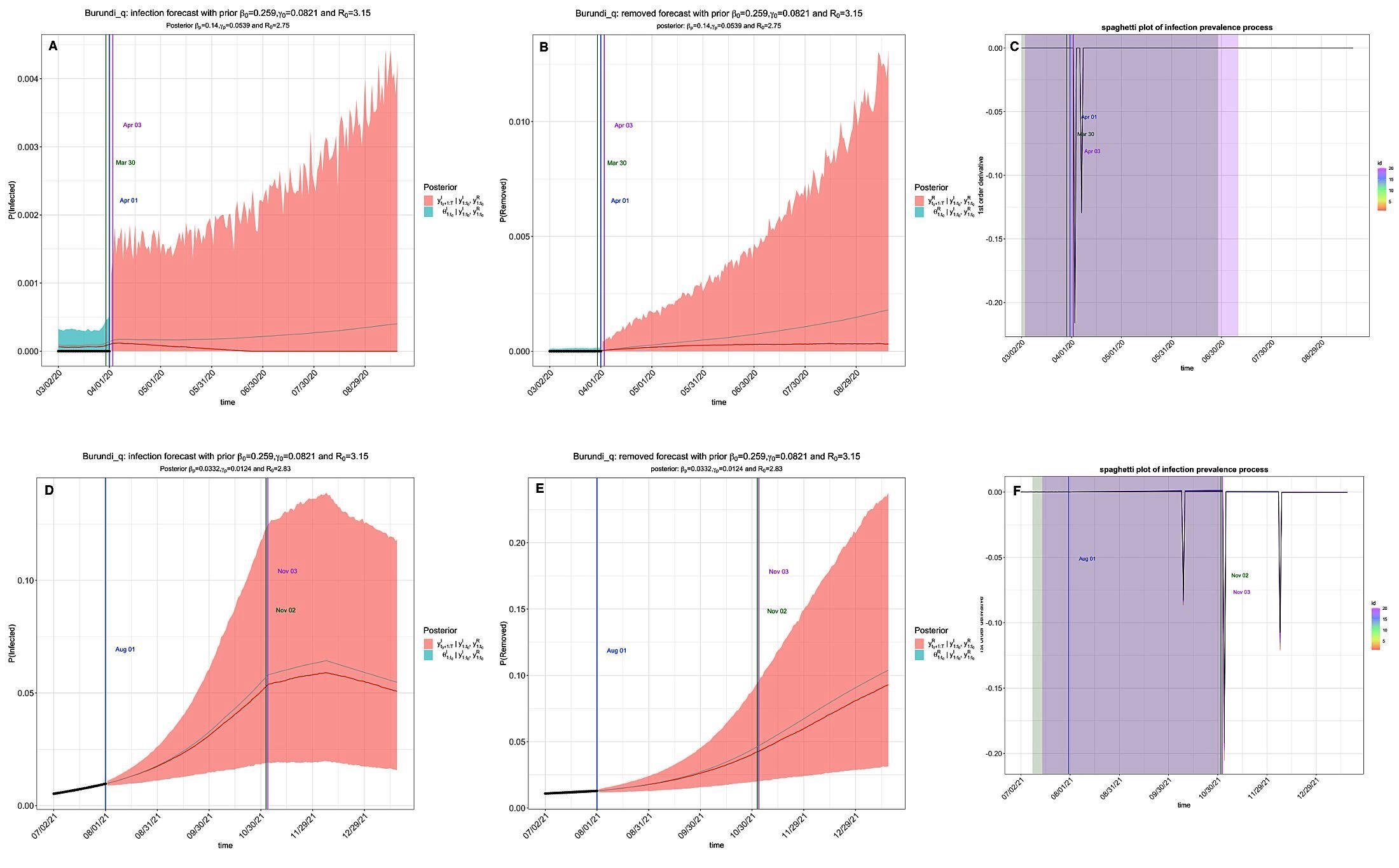
 **Figure S19. Estimation of COVID-19 trend in Burundi using a time-varying quarantine model**. Quarantine restrictions delayed the occurrence of the pandemic peak to late August 2020 (Figure S19 A) and late November 2021 (Figure S19 D). R_0_ increased from 2.75 in 2020 to 2.83 in 2021. (A) Prediction of COVID-19 infection during the 2020/2021 window. The first and second turning points occurred on March 30 and April 01 2020; (B) Prediction of the removed compartment during the 2020/2021 window; (C) Plot of the first-order derivatives of the posterior prevalence of infection in 2020/2021. The colored rectangles represent the 95% CI of the turning points. (D) Prediction of the infection of COVID-19 for 2021/2022. The first and second turning points occurred on August 01 and November 02 2021; (E) Prediction of the removed compartment during 2021/2022 window; (F) Plot of the first-order derivatives of the posterior prevalence of infection during 2021/2022 window.


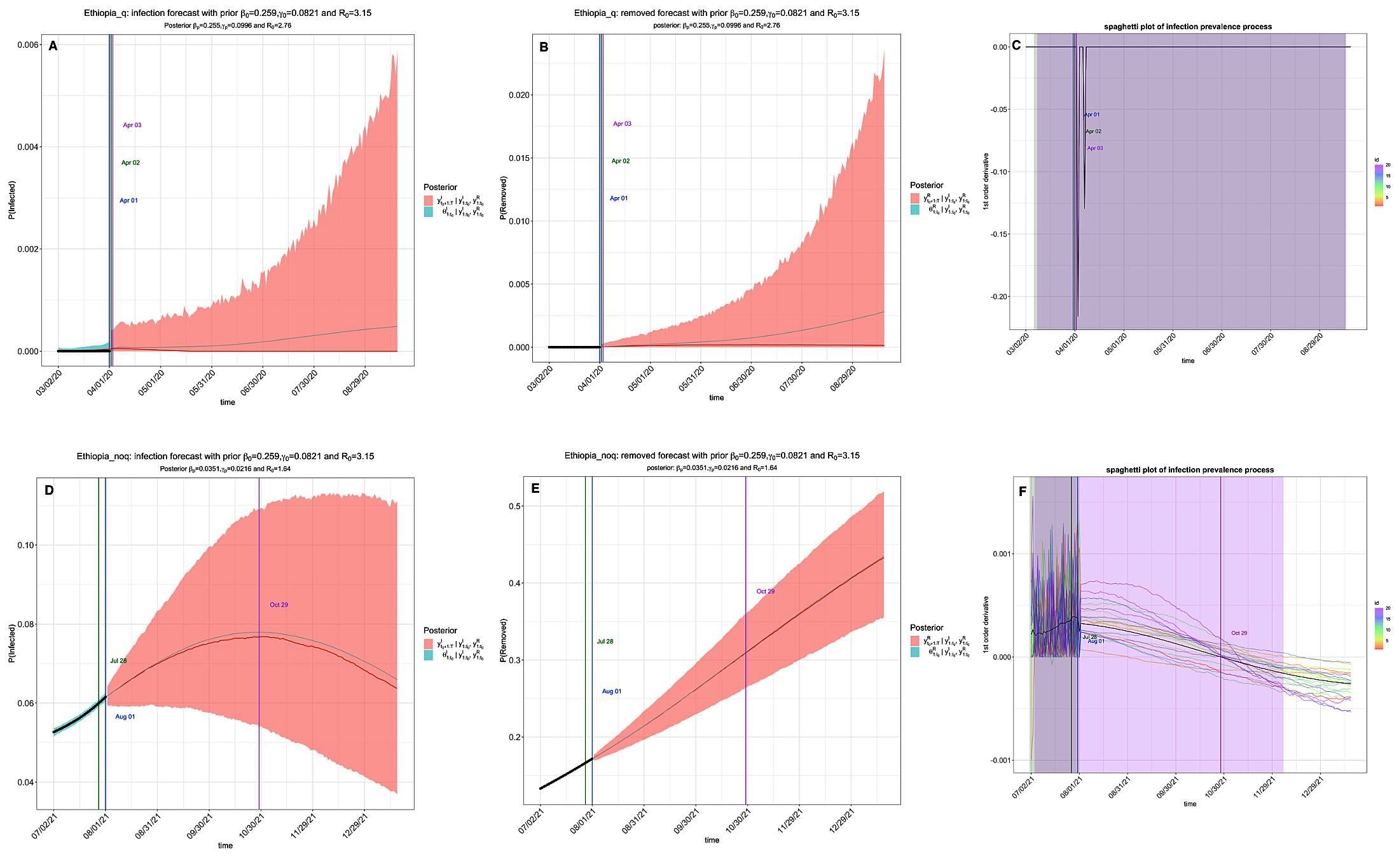
 **Figure S20. Estimation of COVID-19 trend in Ethiopia using a time-varying quarantine model**. Quarantine restrictions delayed the peak of the pandemic to August 29/2020 (Figure S 20 A) and October 30/2021 (Figure S20 D). R_0_ decreased from 2.78 in 2020 to 1.64 in 2021. (A) Prediction of COVID-19 infection during the 2020/2021 window. The first and second turning points occurred on April 01 and April 02 2020; (B) Prediction of the removed compartment during 2020/2021 window; (C) Plot of the first-order derivatives of the posterior prevalence of infection in 2020/2021. The colored rectangles represent the 95% CI of the turning points. (D) Prediction of the infection of COVID-19 for 2021/2022; (E) Prediction of the removed compartment during 2021/2022 window. The first and second turning points occurred on July 28 and August 01 2021; (F) Plot of the first-order derivatives of the posterior prevalence of infection during 2021/2022 window.


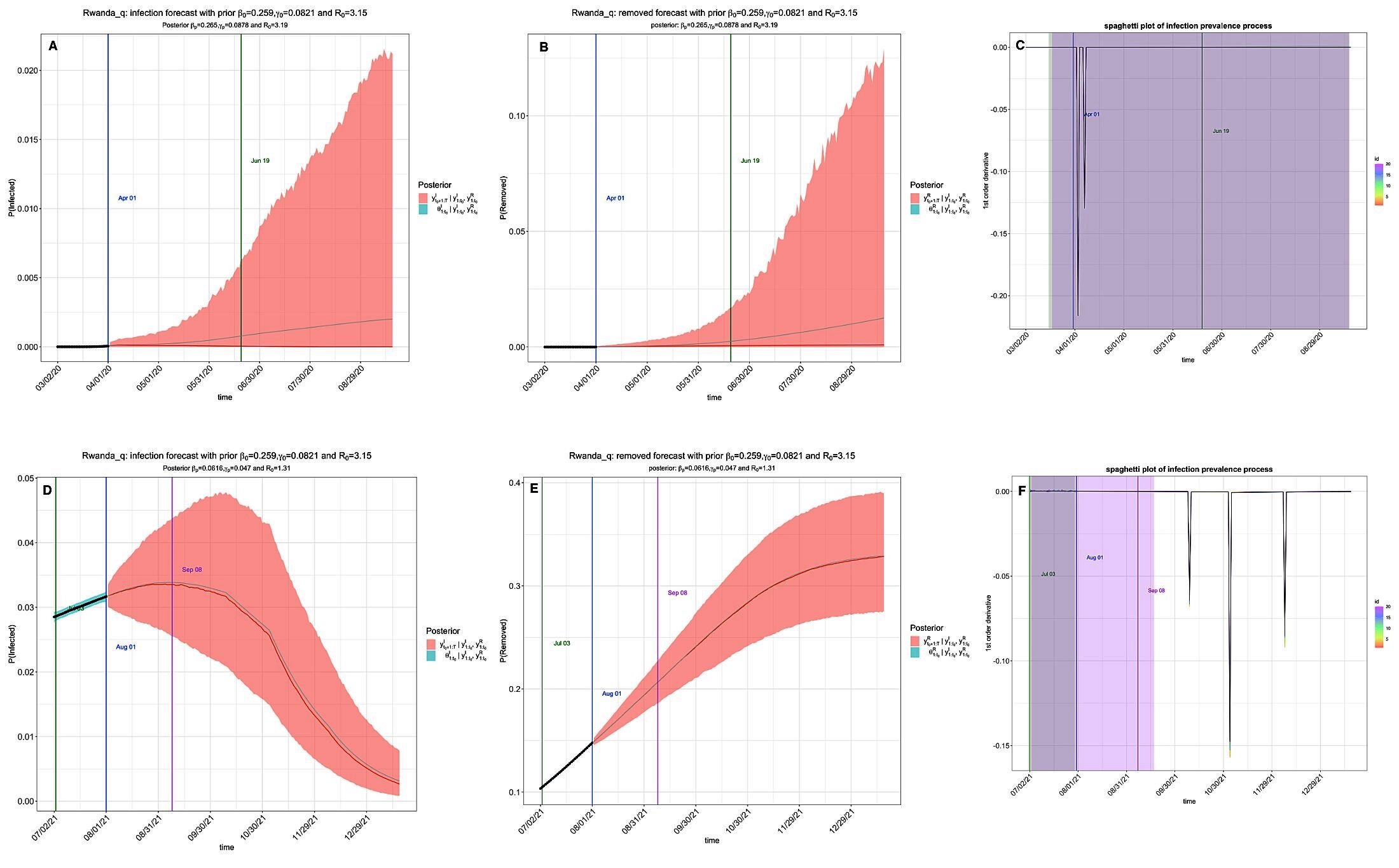
 **Figure S21. Estimation of COVID-19 trend in Rwanda using a time-varying quarantine model**. Quarantine restrictions delayed the peak of the pandemic to August 29/2020 (Figure S21 A) and September 15/2021 (Figure S21 D). R_0_ decreased from 3.19 in 2020 to 1.31 in 2021. (A) Prediction of COVID-19 infection during the 2020/2021 window. The first and second turning points occurred on April 01 and June 19 2020; (B) Prediction of the removed compartment during 2020/2021 window; (C) Plot of the first-order derivatives of the posterior prevalence of infection in 2020/2021. The colored semi-transparent rectangles represent the 95% CI of these turning points. (D) Prediction of the infection of COVID-19 during the 2021/2022 window. The first and second turning points occurred on July 03 and August 01 2021; (E) Prediction of the removed compartment during 2021/2022 window; (F) Plot of the first-order derivatives of the posterior prevalence of infection during the 2021/2022 window.


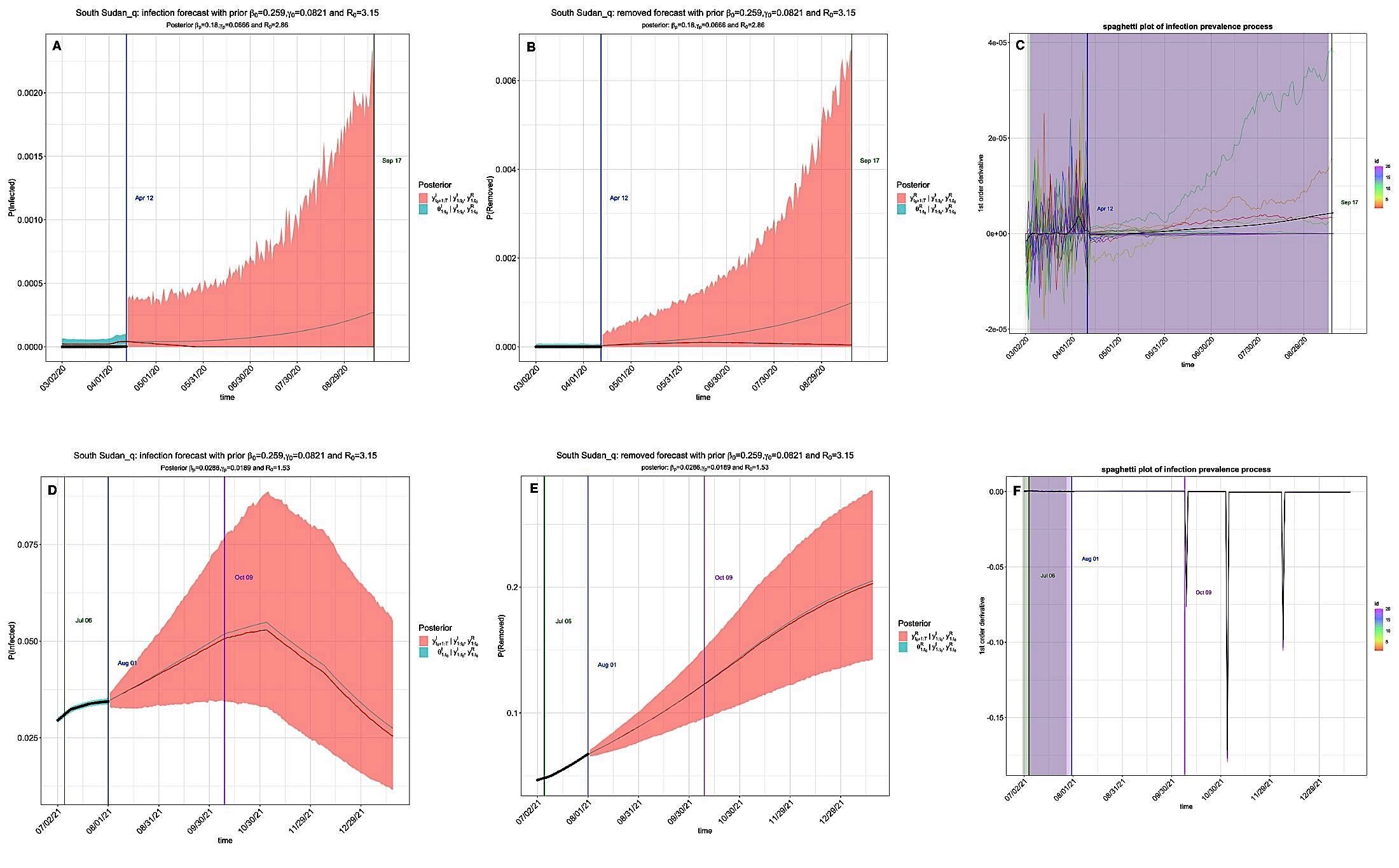
 **Figure S22. Estimation of COVID-19 trend in South Sudan using a time-varying quarantine model**. Quarantine restrictions delayed the peak of the pandemic to September 17/2020 (Figure S22 A) and October 09/2021 (Figure S22 D). R_0_ decreased from 2.86 in 2020 to 1.53 in 2021. (A) Prediction of COVID-19 infection during the 2020/2021 window. The first and second turning points occurred on April 12 and April 13 2020; (B) Prediction of the removed compartment during 2020/2021 window; (C) Plot of the first-order derivatives of the posterior prevalence of infection in 2020/2021. The colored rectangles represent the 95% CI of the turning points. (D) Prediction of the infection of COVID-19 for 2021/2022. The first and second turning points occurred on July 06 and August 01 2021; (E) Prediction of the removed compartment during the 2021/2022 window; (F) Plot of the first-order derivatives of the posterior prevalence of infection during the 2021/2022 window.


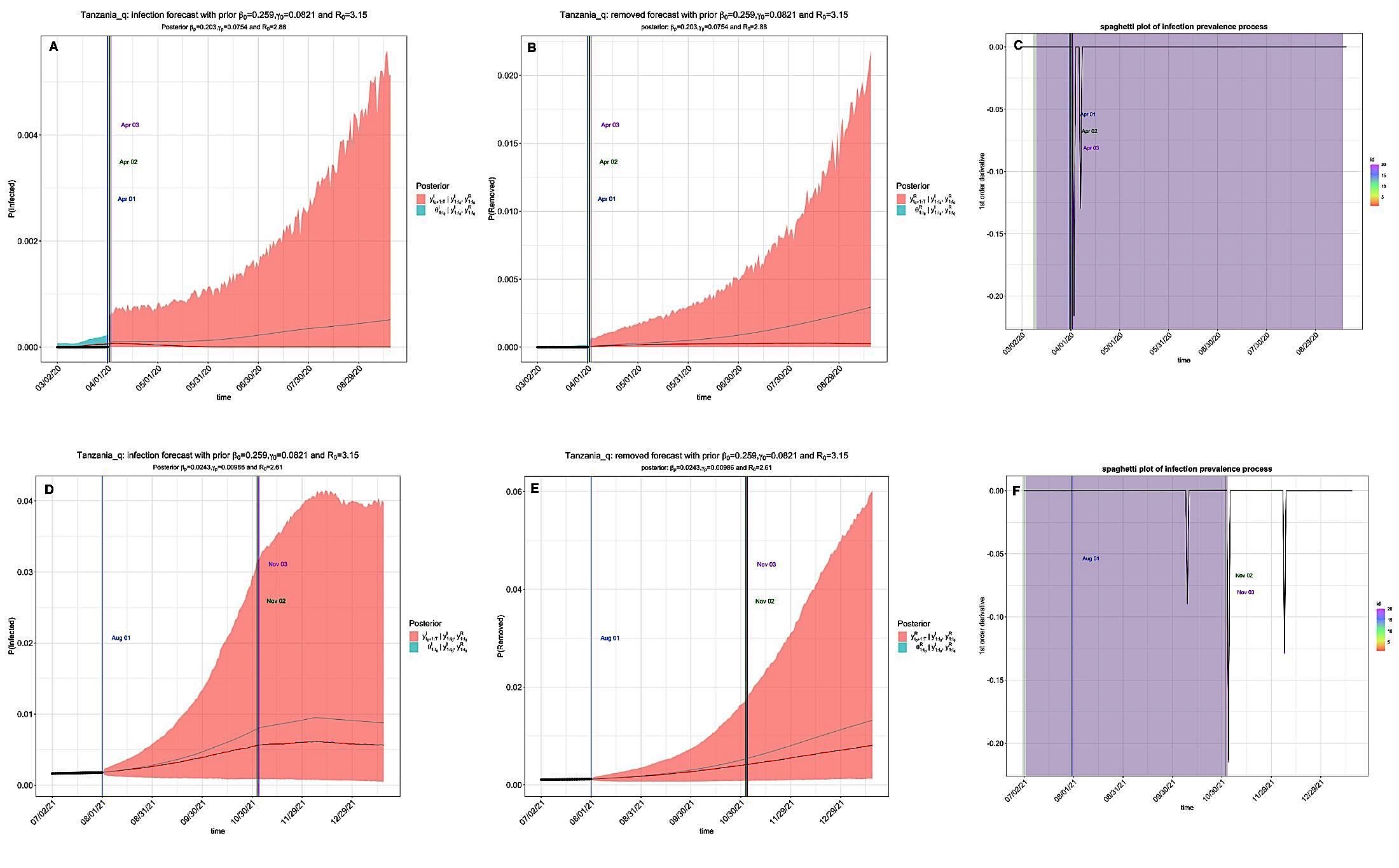
 **Figure S23. Estimation of COVID-19 trend in Tanzania using a time-varying quarantine model**. Quarantine restrictions delayed the peak of the pandemic to early September 2020 (Figure S23 A) and November 29/2021 (Figure S23 D). R_0_ decreased from 2.86 in 2020 to 1.53 in 2021. (A) Prediction of COVID-19 infection during the 2020/2021 window. The first and second turning points occurred on April 01 and April 02 2020; (B) Prediction of the removed compartment during 2020/2021 window; (C) Plot of the first-order derivatives of the posterior prevalence of infection in the 2020/2021 window. The colored rectangles represent the 95% CI of the turning points. (D) Prediction of COVID-19 infection during the 2021/2022 window; (E) Prediction of the removed compartment during the 2021/2022 window. The first and second turning points occurred on August 01 and November 02 2021; (F) Plot of the first-order derivatives of the posterior prevalence of infection during the 2021/2022 window.


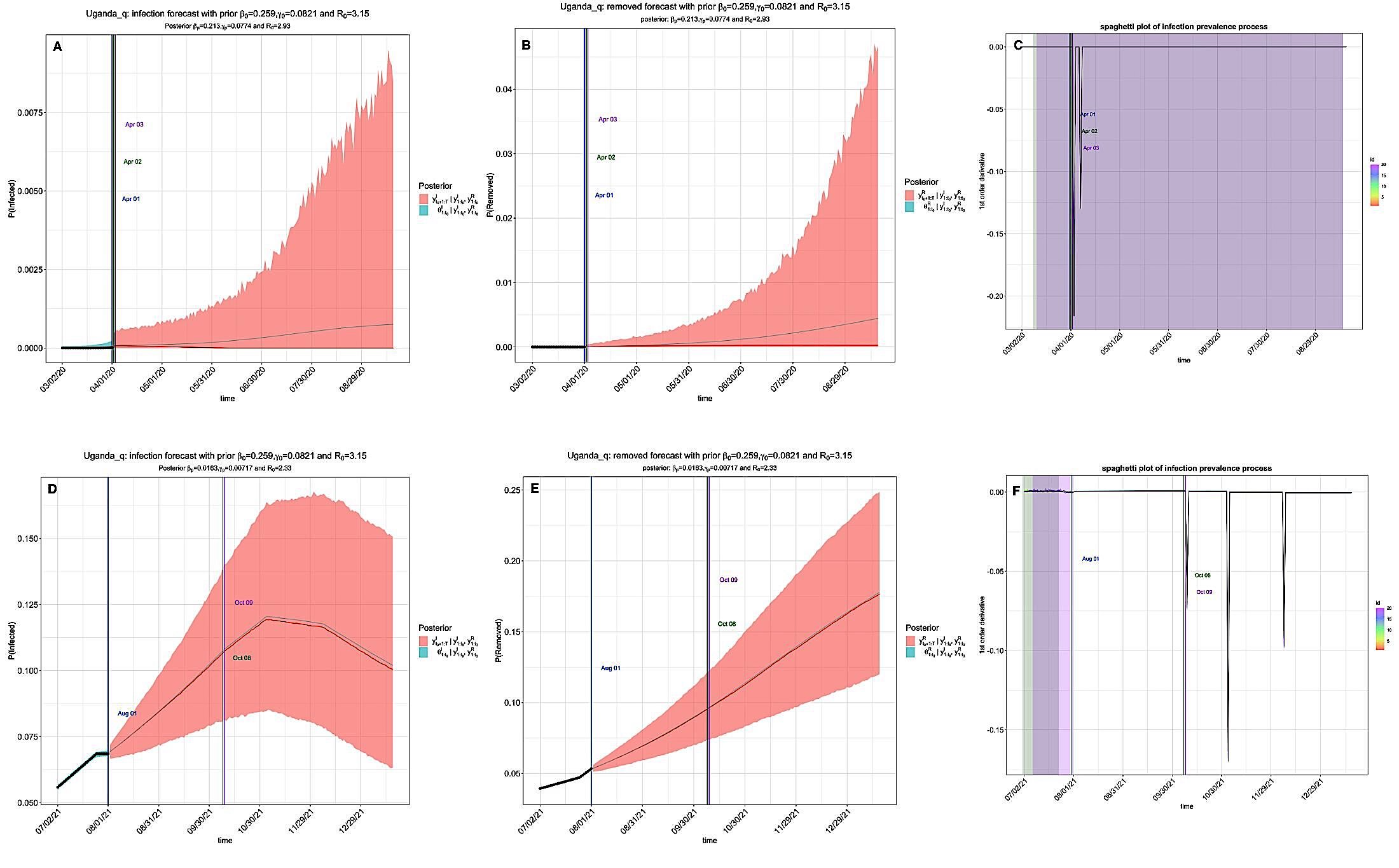
 **Figure S24. Estimation of COVID-19 trend in Uganda using a time-varying quarantine model**. Quarantine restrictions delayed the peak of the pandemic until early September 2020 (Figure S24 A) and October 30/2021 (Figure S24 D). R_0_ decreased from 2.93 in 2020 to 2.33 in 2021. (A) Prediction of COVID-19 infection during the 2020/2021 window. The first and second turning points occurred on April 01 and April 02 2020; (B) Prediction of the removed compartment during the 2020/2021 window; (C) Plot of the first-order derivatives of the posterior prevalence of infection in the 2020/2021. The colored rectangles represent the 95% CI of the turning points. (D) Prediction of COVID-19 infection during 2021/2022 window. The first and second turning points occurred on August 01 and October 08 2021; (E) Prediction of the removed compartment during the 2021/2022 window; (F) Plot of the first-order derivatives of the posterior prevalence of infection during the 2021/2022 window.


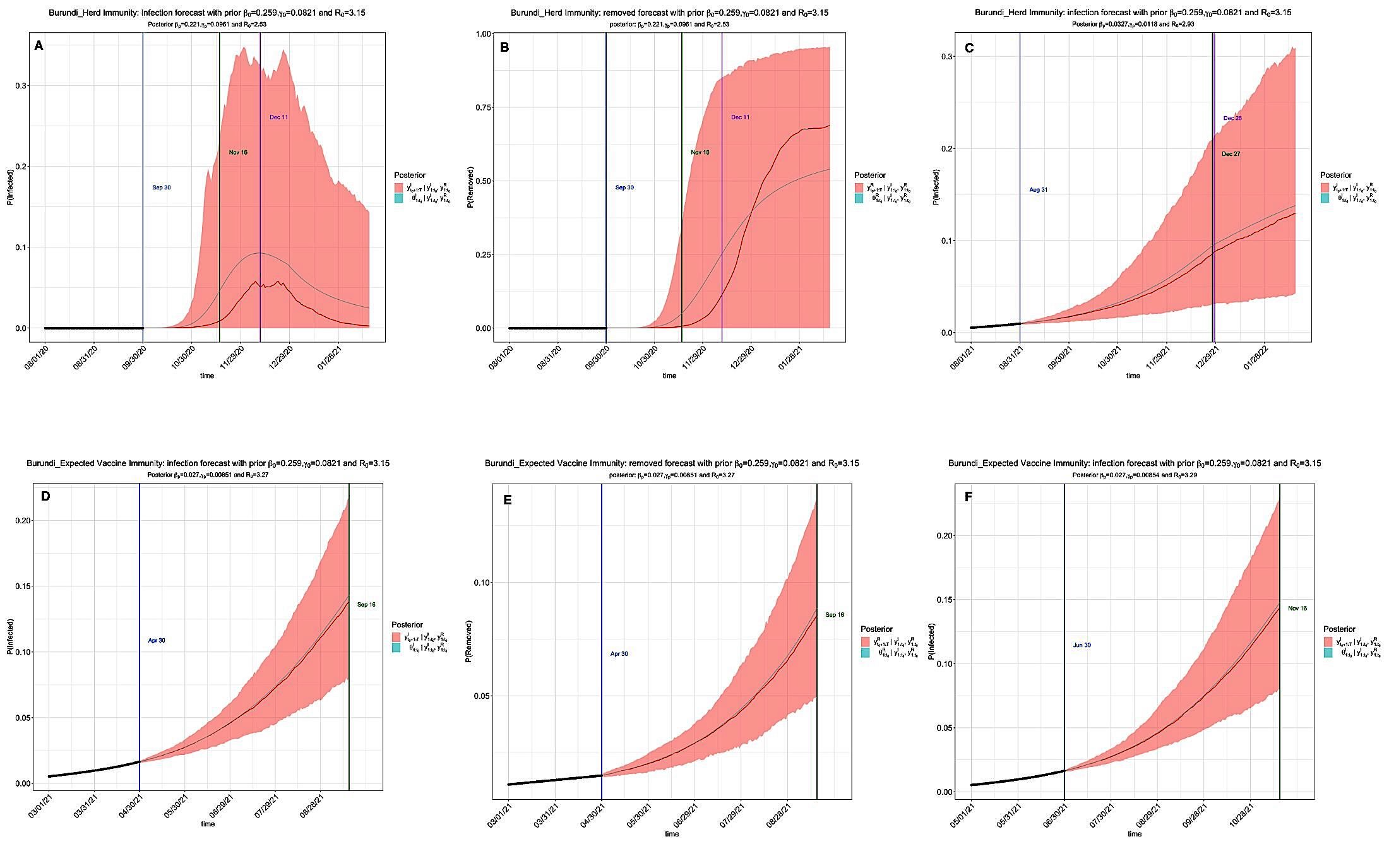
 **Figure S25. Estimation of herd immunity and vaccination campaign in Burundi**. Herd immunity delayed the peak epidemic until November 29/2020 (Figure S25 A) and December 29/2021 (Figure S25 C), assuming that 20% of the population had acquired anti-SARS-COV-2 antibodies. R_0_ increased from 2.53 in 2020 to 2.93 in 2021. Vaccination delayed the peak epidemic to November 16/2021 assuming that 2% of the population was inoculated (Figure S25 F). Similarly, R_0_ increased from 3.27 to 3.29 in 2021. (A) Prediction of COVID-19 infection if 20% of the population had acquired antibodies against SARS-COV-2. The first and second turning points occurred on September 30 and November 18 2020; (B) Prediction of the removed compartment under herd immunity; (C) Prediction of COVID-19 infection during the 2021/2022 window if 20% of the population had acquired antibodies against SARS-COV-2. The first and second turning points occurred on August 31/2021 and December 27/2021; (D) Prediction of COVID-19 infection during the 2020/2021 window if 2% of the population was vaccinated. The first and second turning points occurred on April 30/2021 and September 16/2021; (E) Prediction of the removed compartment if 2% of the population was vaccinated; (F) Prediction of the infection during the 2021/2022 window if 2% of the population was vaccinated. The first and second turning points occurred on June 30/2021 and November 16/2021.


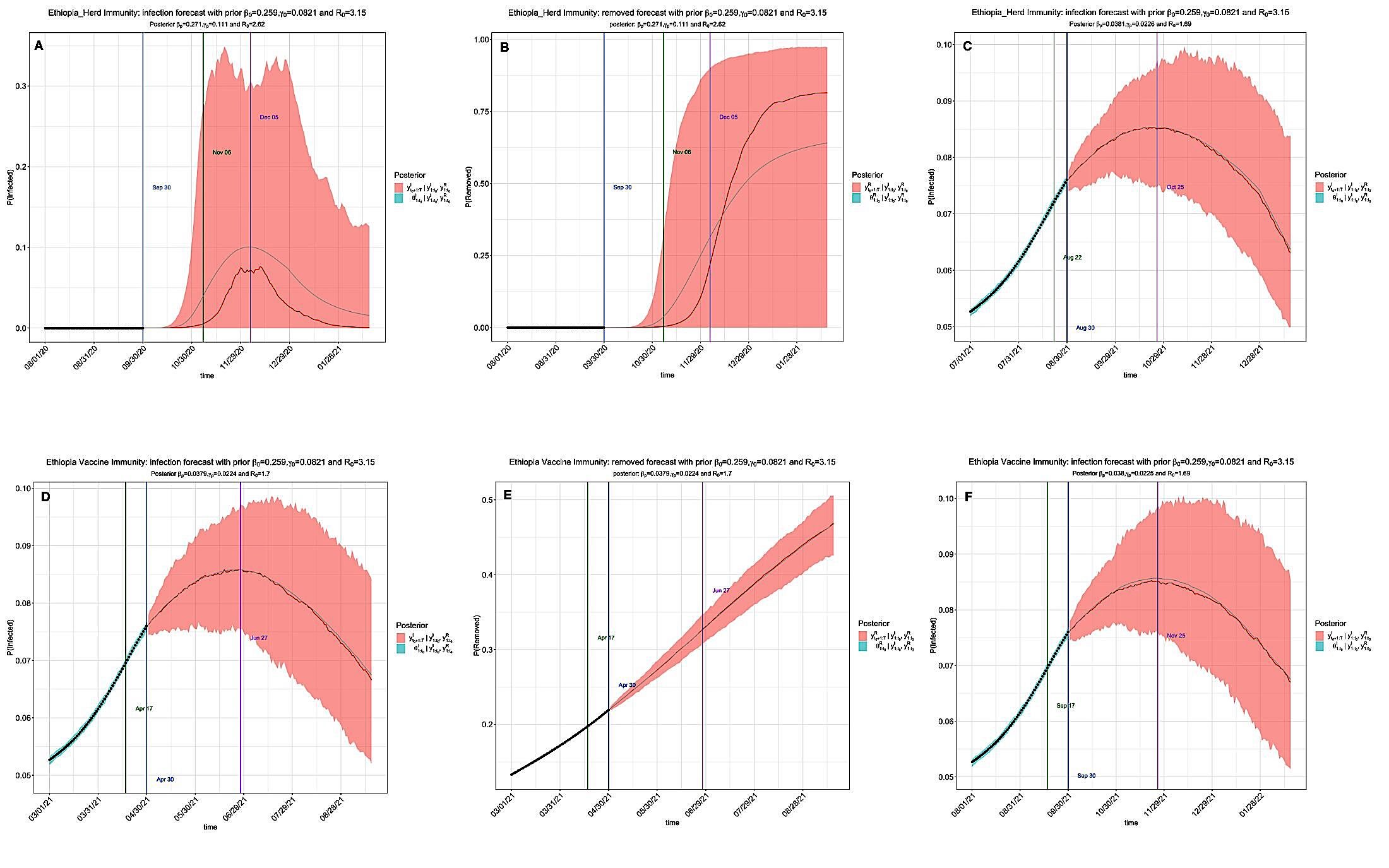
 **Figure S26. Estimation of herd immunity and vaccination campaign in Ethiopia**. Herd immunity delayed the peak epidemic until November 29/2020 (Figure S26 A) and October 29/2021 (Figure S26 C), assuming that 20% of the population was immune to COVID-19. R_0_ decreased from 2.62 in 2020 to 1.69 in 2021. Vaccination delayed the peak epidemic to November 29/2021 (Figure S26 F), assuming that 2% of the population was inoculated. R_0_ decreased from 1.70 to 1.69 in 2021. (A) Prediction of COVID-19 infection during the 2020/2021 window if 20% of the population had acquired antibodies against SARS-COV-2. The first and second turning points occurred on September 30/2020 and November 06/2020; (B) Prediction of the removed compartment under herd immunity; (C) Prediction of COVID-19 infection during the 2021/2022 window if 20% of the population had acquired antibodies against SARS-COV-2. The first and second turning points occurred on August 22/2021 and August 30/2021; (D) Prediction of COVID-19 infection during the 2020/2021 window if 2% of the population was vaccinated. The first and second turning points occurred on April 17/2021 and April 30/2021; (E) Prediction of the removed compartment if 2% of the population was vaccinated; (F) Prediction of COVID-19 infection in the 2021/2022 window if 2% of the population was vaccinated. The first and second turning points occurred on September 17/2021 and September 30/2021.


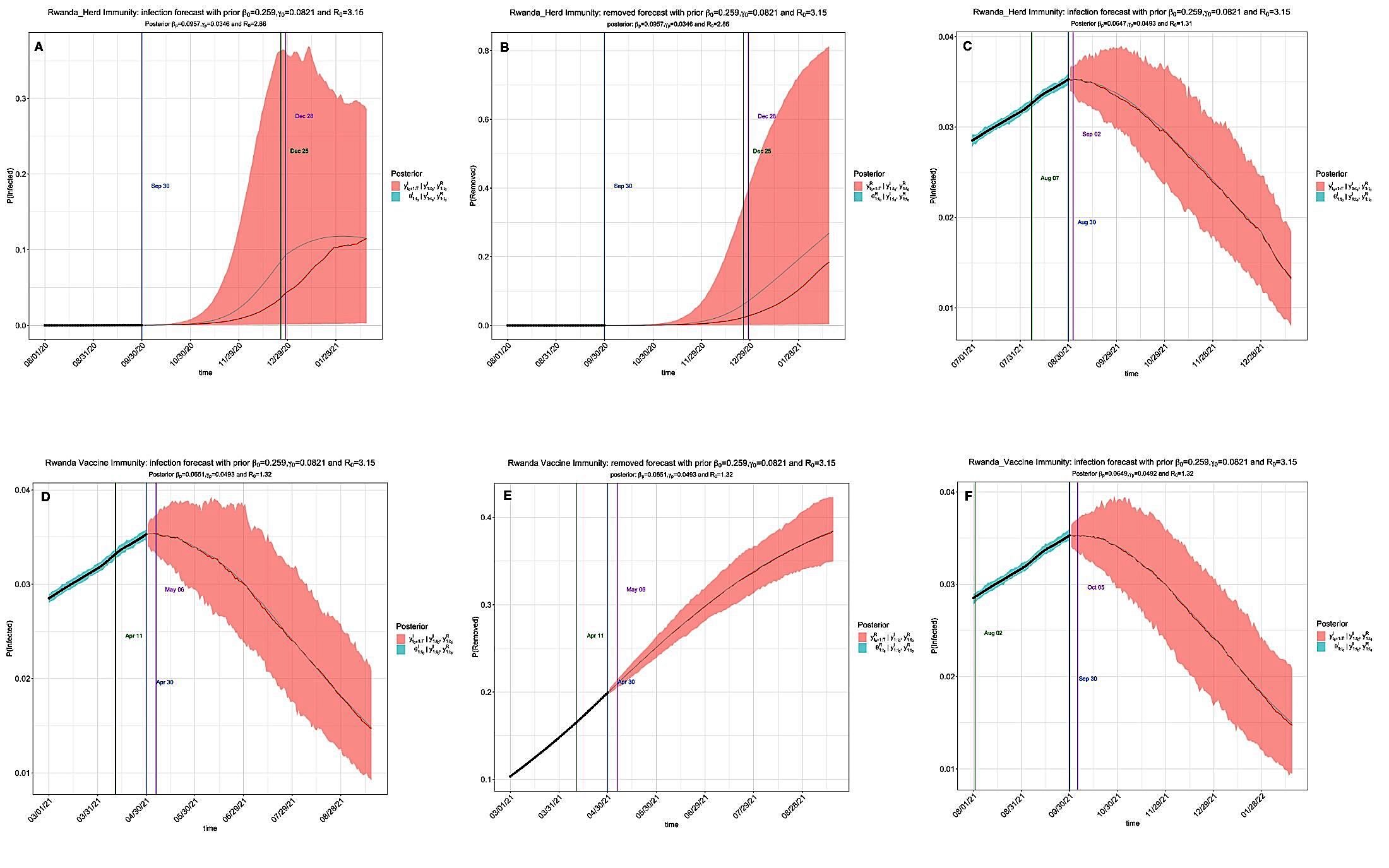
 **Figure S27. Estimation of herd immunity and vaccination campaign in Rwanda**. Herd immunity delayed the peak epidemic to December 25/2020 (Figure S27 A) and September 29/2021 (Figure S27 C), assuming that 20% of the population was immune to COVID-19. R_0_ decreased from 2.86 in 2020 to 1.31 in 2021. Vaccination delayed the peak epidemic until October 30/2021 assuming that 2% of the population was inoculated (Figure S27 F). R_0_ stabilized at 1.32 in 2021. (A) Prediction of COVID-19 infection during the 2020/2021 window if 20% of the population had acquired antibodies against SARS-COV-2. The first and second turning points occurred on September 30/2020 and December 25/2020; (B) Prediction of the removed compartment under herd immunity; (C) Prediction of COVID-19 infection during the 2021/2022 window if 20% of the population had acquired antibodies against SARS-COV-2. The first and second turning points occurred on August 07/2021 and August 30/2021; (D) Prediction of COVID-19 infection during the 2020/2021 window if 2% of the population was vaccinated. The first and second turning points occurred on April 11/2021 and April 30/2021; (E) Prediction of the removed compartment if 2% of the population was vaccinated; (F) Prediction of COVID-19 infection during the 2021/2022 window if 2% of the population was vaccinated. The first and second turning points occurred on August 02/2021 and September 30/2021.


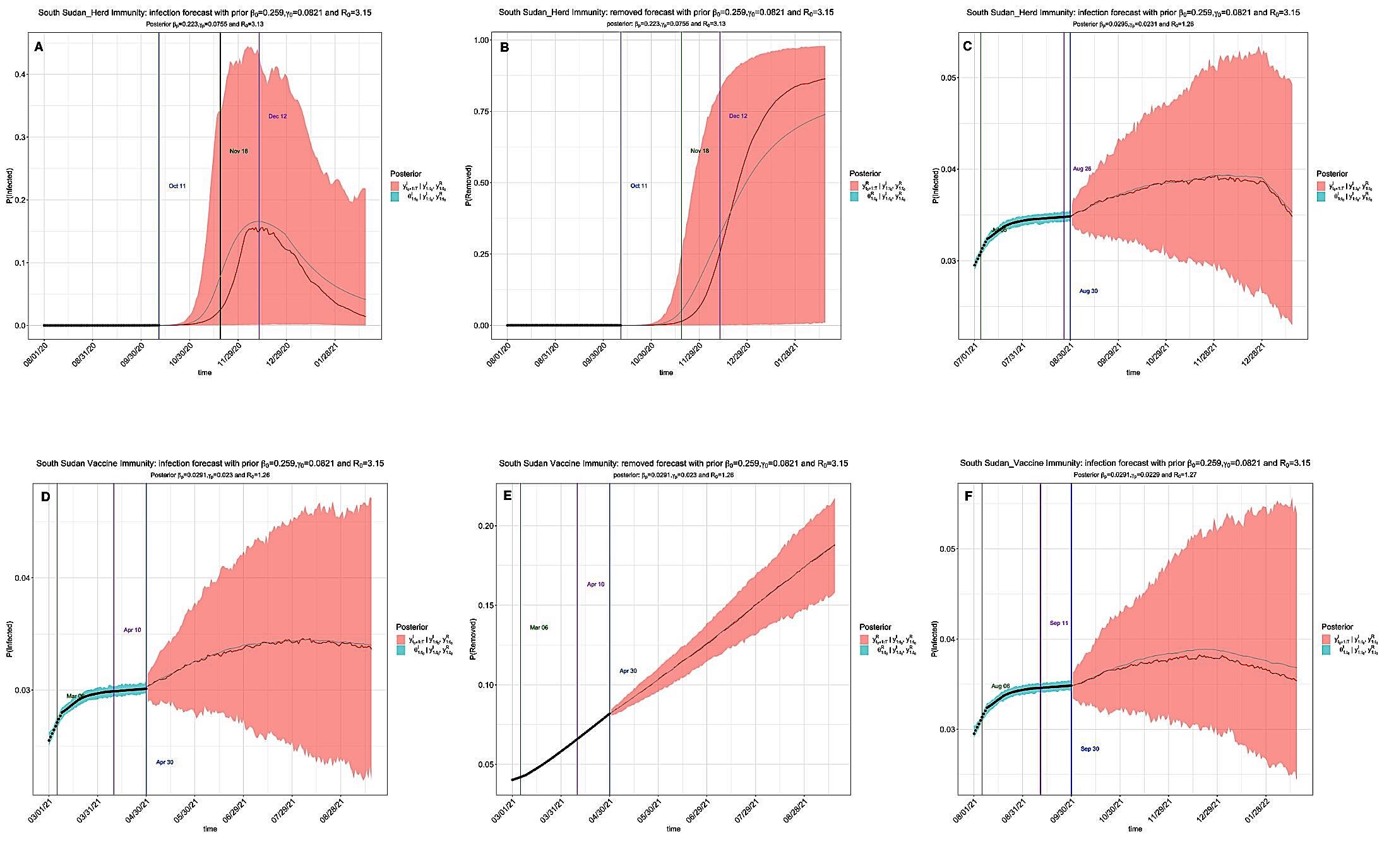
 **Figure S28. Estimation of herd immunity and vaccination campaign in South Sudan**. Herd immunity delayed the simulated peak epidemic to November 30/2020 (Figure S28 A) and December 28/2021 (Figure S28 C), assuming that 20% of the population had acquired anti-SARS-COV-2 antibodies. R_0_ decreased from 3.13 in 2020 to 1.28 in 2021. Vaccination delayed the peak epidemic to July 29/2021 (Figure S28 D) and December 29/2021 (Figure S28 F) if 2% of the population was inoculated. R_0_ marginally increased from 1.26 to 1.27 in 2021. (A) Prediction of COVID-19 infection during the 2020/2021 window if 20% of the population had acquired antibodies against SARS-COV-2. The first and second turning points occurred on October 11/2020 and November 18/2020; (B) Prediction of the removed compartment under herd immunity; (C) Prediction of COVID-19 infection during the 2021/2022 window if 20% of the population had acquired antibodies against SARS-COV-2. The first and second turning points occurred on July 05/2021 and August 25/2021; (D) Prediction of COVID-19 infection during the 2020/2021 window if 2% of the population was vaccinated. The first and second turning points occurred on March 06/2021 and April 10/2021; (E) Prediction of the removed compartment if 2% of the population was vaccinated; (F) Prediction of COVID-19 infection during the 2021/2022 window if 2% of the population was vaccinated. The first and second turning points occurred on August 06/2021 and September 11/2021.


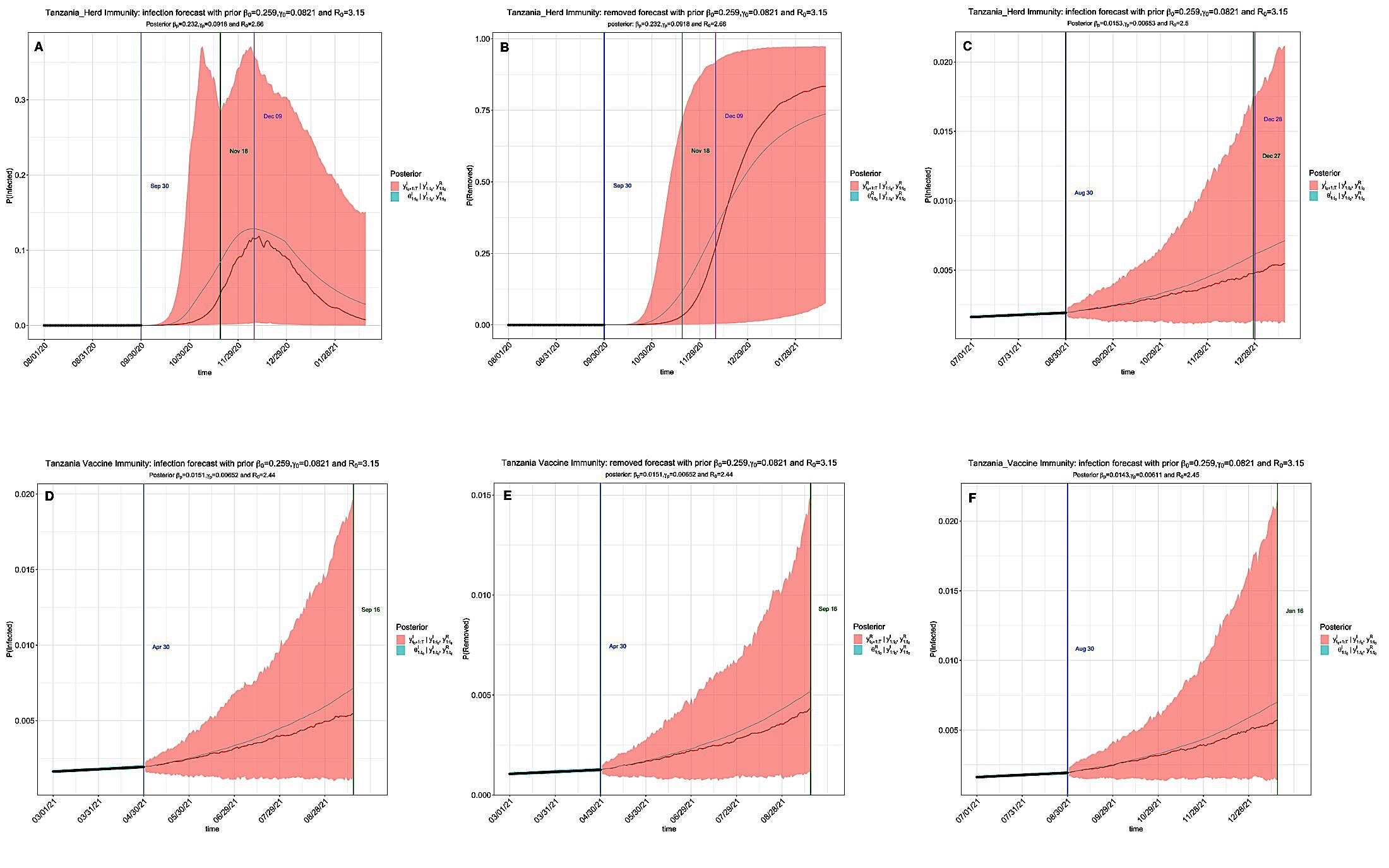
 **Figure S29. Estimation of herd immunity and vaccination campaign in Tanzania**. Herd immunity delayed the simulated peak epidemic until December 09/2020 (Figure S29 A) and December 30/2021 (Figure S29 C), assuming that 20% of the population had acquired anti-SARS-COV-2 antibodies. R_0_ decreased from 2.66 in 2020 to 2.50 in 2021. Vaccination delayed the peak epidemic until September 16/2021 (Figure S29 D) and January 16/2022 (Figure S29 F), assuming that 2% of the population was inoculated. R_0_ marginally increased from 2.44 to 2.45 in 2021. (A) Prediction of COVID-19 infection during 2020/2021 window if 20% of the population had antibodies against SARS-COV-2. The first and second turning points occurred on September 30/2020 and November 18/2020; (B) Prediction of the removed compartment under herd immunity; (C) Prediction of COVID-19 infection during the 2021/2022 window if 20% of the population had acquired antibodies against SARS-COV-2. The first and second turning points occurred on August 30/2021 and December 27/2021; (D) Prediction of COVID-19 infection during the 2020/2021 window if 2% of the population was vaccinated. The first and second turning points occurred on April 30/2021 and September 16/2021; (E) Prediction of the removed compartment if 2% of the population was vaccinated; (F) Prediction of COVID-19 infection during the 2021/2022 window if 2% of the population was vaccinated. The first and second turning points occurred on August 30/2021 and January 16/2021.


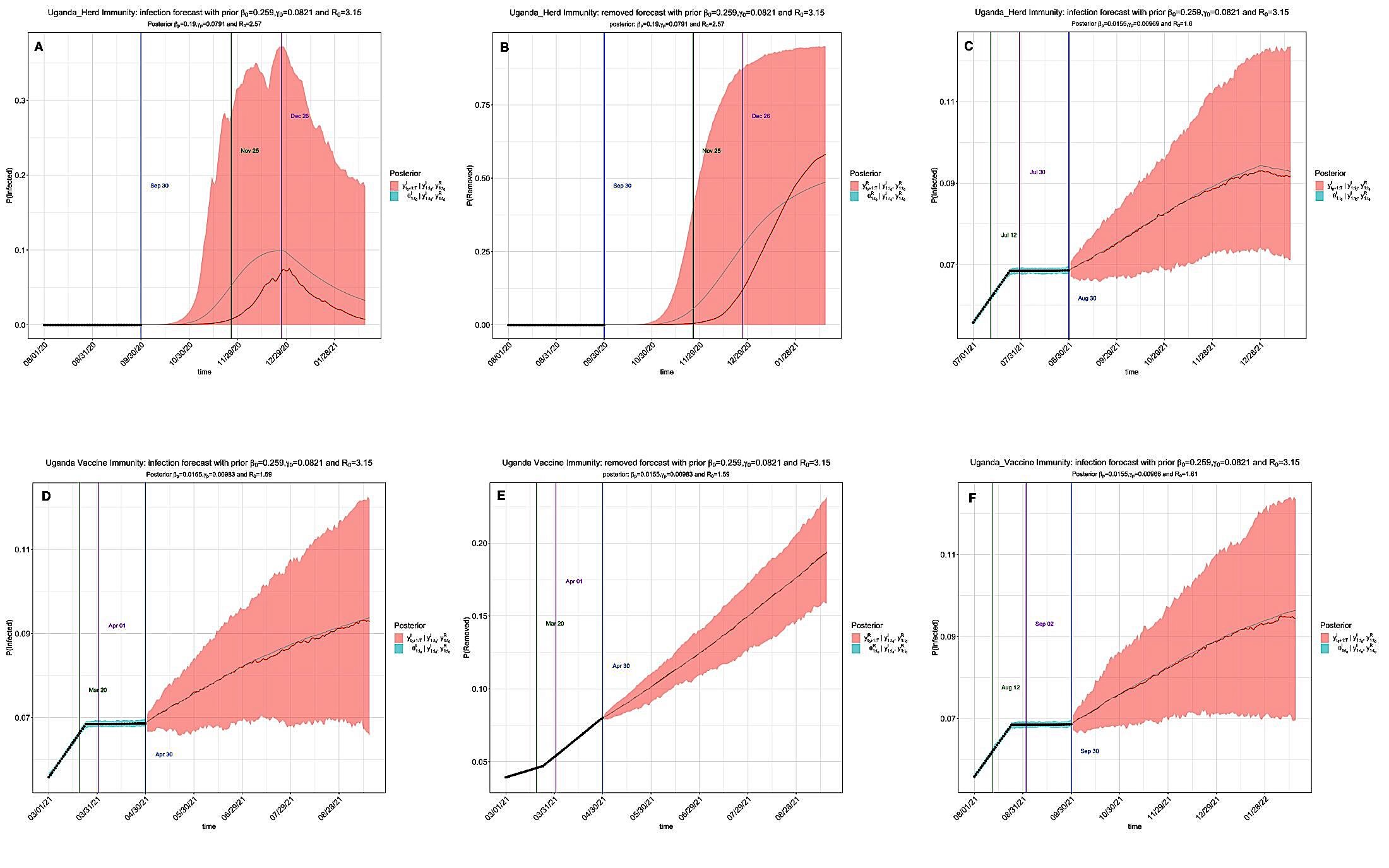
 **Figure S30. Estimation of herd immunity and vaccination campaign in Uganda**. Herd immunity delayed the peak epidemic to December 29/2020 (Figure S30 A) and December 28/2021 (Figure S30 C), assuming that 20% of the population was immune to COVID-19. R_0_ decreased from 2.57 in 2020 to 1.60 in 2021. Vaccination delayed the peak epidemic until August 28/2021 (Figure S30 D) and January 28/2022 (Figure S30 F), assuming that 2% of the population was inoculated. R_0_ marginally increased from 1.59 to 1.61 in 2021. (A) Prediction of COVID-19 infection if 20% of the population had acquired antibodies against SARS-COV-2. The first and second turning points occurred on September 30/2020 and November 25/2020; (B) Prediction of the removed compartment under herd immunity; (C) Prediction of COVID-19 infection during 2021/2022 window if 20% of the population had acquired antibodies against SARS-COV-2. The first and second turning points occurred on July 12/2021 and July 30/2021; (D) Prediction of COVID-19 infection during the 2020/2021 window if 2% of the population was vaccinated. The first and second turning points occurred on March 20/2021 and April 01/2021; (E) Prediction of the removed compartment if 2% of the population was vaccinated; (F) Prediction of COVID-19 infection during the 2021/2022 window if 2% of the population was vaccinated. The first and second turning points occurred on August 12/2021 and September 02/2021.


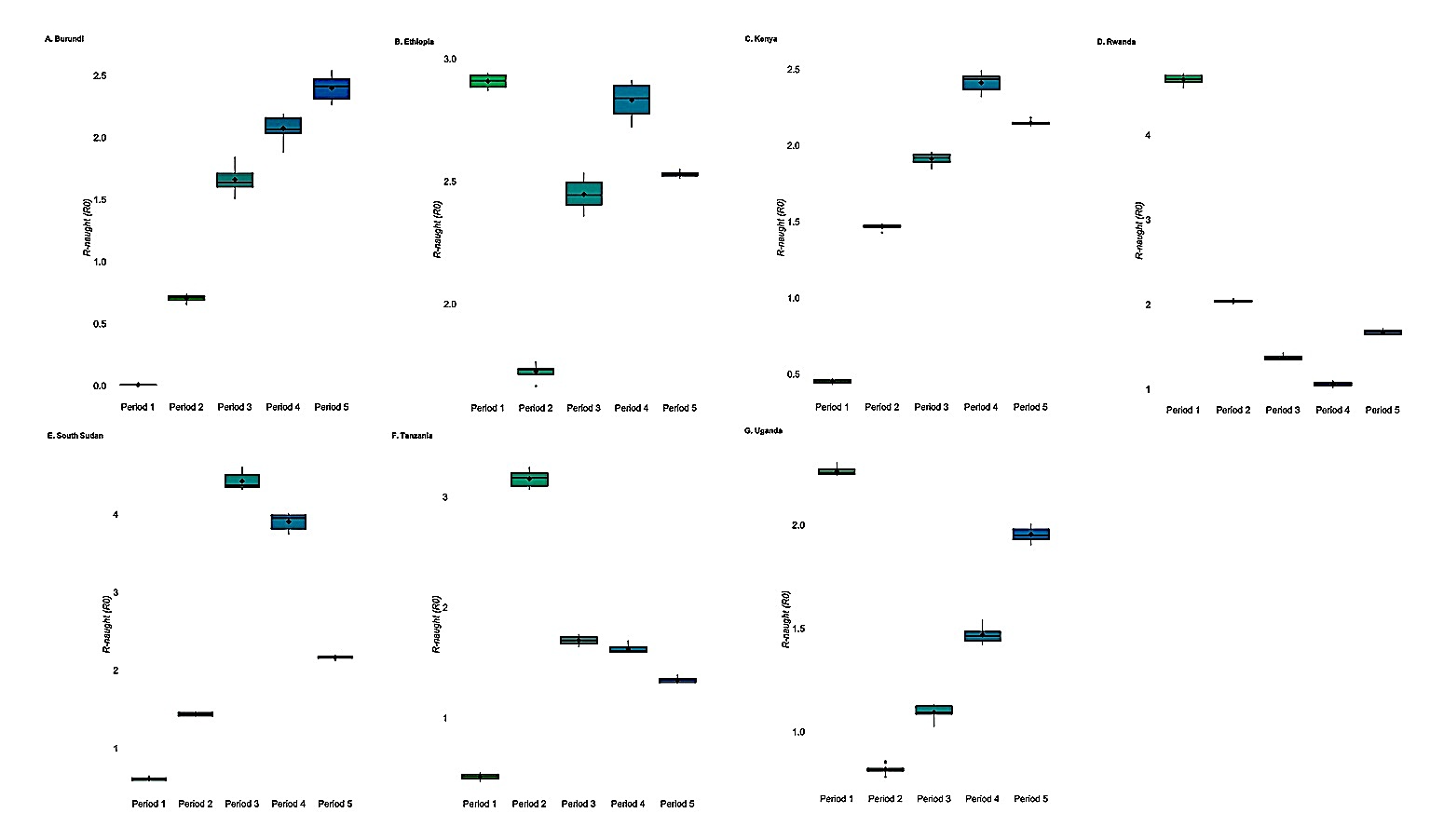
 **Figure S31. Estimates of R_0_ across EACs provided using the multinomial-2-parameter SEIR-fansy model.** R_0_ values were generated for the five time periods starting from the day of onset of the pandemic up to the predicted end-point.
